# Tumor-derived neutrophil extracellular trap–associated DNA impairs treatment efficacy in breast cancer via CCDC25-dependent epithelial-mesenchymal transition

**DOI:** 10.1172/JCI190557

**Published:** 2026-01-02

**Authors:** Heliang Li, Yetong Zhang, Jianghua Lin, Jiayi Zeng, Xinyan Liang, Linxi Xu, Jiang Li, Xiaoming Zhong, Xu Liu, Zhou Liu, Xinyu Yang, Yunyi Zhang, Shun Wang, Erwei Song, Man Nie, Linbin Yang

**Affiliations:** 1 Breast Tumor Center, Guangdong Provincial Key Laboratory of Malignant Tumor Epigenetics and Gene Regulation, Medical Research Center, and; 2 Department of Thyroid Surgery, Sun Yat-Sen Memorial Hospital, Sun Yat-Sen University, Guangzhou, Guangdong, China.; 3 Department of Breast and Thyroid Surgery, Renmin Hospital of Wuhan University, Wuhan, Hubei, China.; 4 Zenith Institute of Medical Sciences, Guangzhou, Guangdong, China.; 5 State Key Laboratory of Proteomics, Beijing, China.; 6 Department of Medical Oncology, State Key Laboratory of Oncology in South China, Guangdong Provincial Clinical Research Center for Cancer, Collaborative Innovation Center for Cancer Medicine, Sun Yat-sen University Cancer Center, Guangzhou, Guangdong, China.

**Keywords:** Immunology, Oncology, Breast cancer, Macrophages, Therapeutics

## Abstract

Neutrophil extracellular traps (NETs) are associated with cancer progression; however, the functional role and clinical importance of NET-DNA in therapeutic resistance remain unclear. Here, we show that chemotherapy and radiotherapy provoke NET-DNA formation in primary tumor and metastatic organs in breast cancer patients and mouse models, and the level of NET-DNA correlates with treatment resistance. Mechanistically, the cathepsin C in tumor debris generated by anticancer therapy is phagocytosed by macrophages and drives CXCL1/2 and complement factor B production via activating the TLR4/NF-κB signaling pathway, subsequently promoting NETosis and impairing therapeutic efficacy. Importantly, we demonstrate that NET-DNA sensor CCDC25 is indispensable in NET-mediated treatment resistance by inducing cancer cell epithelial-mesenchymal transition via pyruvate kinase isoform M2–mediated STAT3 phosphorylation. Clinically, tumoral CCDC25 abundance is closely associated with poor prognosis in patients who underwent chemotherapy. Overall, our data reveal the mechanism of NET formation and elucidate the interaction of NET-CCDC25 in therapy resistance, highlighting CCDC25 as an appealing target for anticancer interventions.

## Abstract 2

Tumor debris-induced neutrophil extracellular trap-associated DNA impairs treatment efficacy of chemotherapy and radiotherapy via CCDC25-dependent endothelial-to-mesenchymal transition in breast cancer

## Graphical abstract

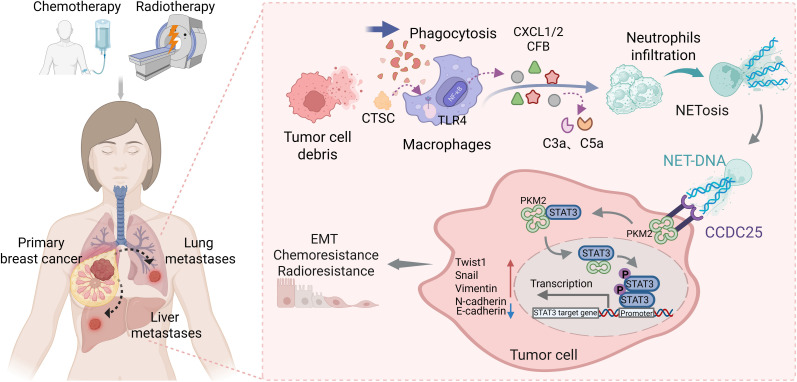

## Introduction

Breast cancer is the leading cause of cancer-related mortalities among women worldwide (1). While surgery is the primary curative treatment for early-stage disease, chemotherapy and radiotherapy are essential for reducing recurrence. In metastatic disease, systemic chemotherapy is the primary therapeutic approach (2). However, the emergence of resistance to chemo/radiotherapy remains a major clinical hurdle, substantially compromising patient survival outcomes (3, 4). These cytotoxic therapies not only directly kill tumor cells but also trigger the release of damage-associated molecular patterns (DAMPs), including nuclear proteins, nucleic acids, and cytokines (5). Although these danger signals can stimulate protective antitumor immunity, thereby enhancing therapeutic efficacy (6), they simultaneously contribute to the formation of an immunosuppressive microenvironment that drives immune dysfunction (7). However, the precise mechanisms by which DAMPs modulate tumor immunity and influence treatment outcomes are not yet fully elucidated.

Extracellular NET-DNA, the DNA component of neutrophil extracellular traps (NETs), is a crucial component of danger signals. It forms a web-like structure composed of decondensed DNA decorated with granular proteins and is released by neutrophils (8, 9). NET-DNA has been initially identified as an antimicrobial defender and implicated in the pathophysiology of infectious disorders and various systemic autoimmune diseases (10). Recently, increasing studies have linked NET-DNA to tumor progression, demonstrating that targeting NET-DNA significantly inhibits cancer metastasis (11, 12). However, its role in chemotherapy and radiotherapy resistance remains largely unclear. Besides, some studies indicate that NET-DNA also contributes to the maturation of dendritic cells and the activation of T cell–mediated immune responses (13, 14). Targeting NET-DNA by DNase I can spare tissue-damaging proteins like histones and proteases, which are known to cause severe tissue or organ injury (15). Therefore, it is necessary to explore a more efficient approach for NET-DNA blockade that minimizes tissue damage while preserving immune response.

In this study, we aim to investigate the role of NET-DNA in conferring resistance to chemotherapy and radiotherapy in breast cancer. Single-cell RNA sequencing (scRNA-Seq) of murine tumors reveals therapy-induced neutrophil infiltration and NET release (NETosis), corroborated in patients with breast cancer receiving neoadjuvant chemotherapy. Mechanistically, macrophage-engulfed cathepsin C (CTSC) from tumor debris activates TLR4/NF-κB signaling, upregulating CXCL1/2 and complement factor B (CFB) to drive neutrophil recruitment and NET formation. Besides, CCDC25, the specific transmembrane NET-DNA receptor (16), correlates with poor clinical treatment response, and its blockade overcomes the NET-dependent chemotherapy and radiotherapy resistance in vivo and in vitro. Furthermore, NET-CCDC25 interaction leads to pyruvate kinase isoform M2 (PKM2)/STAT3–mediated epithelial-mesenchymal transition (EMT) in cancer cells and eventually results in therapeutic resistance.

## Results

### Chemotherapy and radiotherapy promote NET generation in breast cancer.

**Figure 1:**
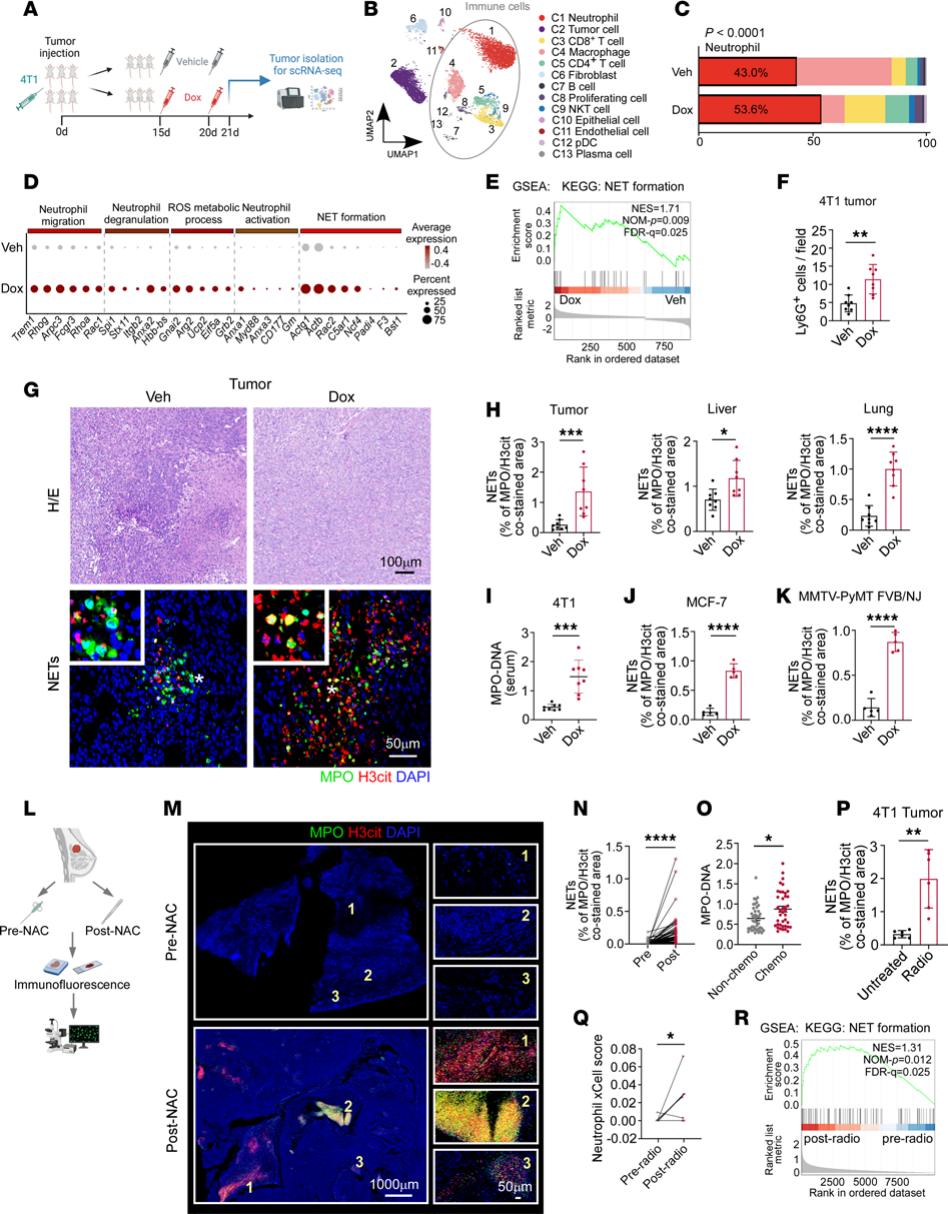
Chemotherapy and radiotherapy promote NET generation in breast cancer. (**A**) Workflow of scRNA-Seq of 4T1 tumors (*n* = 3/group). (**B**) Uniform manifold approximation and projection (UMAP) plot of cell subsets from vehicle (Veh) and Dox groups. The gray circle represents immune cells. (**C**) Stacked bar graph for percentage of immune cells (χ^2^ test). Bar colors represent corresponding subtypes described in **B**. (**D**) Expression levels of differentially expressed genes (DEGs) from GO and KEGG terms in neutrophils (adj. *P* < 0.05). Dot size and color indicate fraction of cells and average expression. (**E**) GSEA revealed enrichment of NET formation pathway in neutrophils from Dox group (permutation test). NOM, normalized. (**F**–**I**) Tumors, metastatic livers, lungs, and serum from 4T1-bearing mice were isolated and analyzed (*n* = 8/group). (**F**) Quantification of tumoral Ly6G^+^ cells (unpaired *t* test). (**G**) Representative H&E images and immunofluorescence images. Scale bars, 50 μm and 100 μm. * indicates areas magnified in the top-left insets. (**H**) Quantification of NET expression. Tumor, Mann-Whitney test. Liver and lung, unpaired *t* test. (**I**) Serum levels of MPO-DNA (Mann-Whitney test). (**J** and **K**) Quantification of NET expression in MCF-7 tumors (**J**) and MMTV-PyMT FVB/NJ mouse tumors (**K**) (*n* = 5/group, unpaired *t* test). (**L**) Schematic illustration. (**M** and **N**) Representative immunofluorescence images (**M**) and quantification (**N**) in paired pre- and post-NAC breast tumors (*n* = 81 pairs, Wilcoxon matched pairs signed-rank test). Scale bars, 50 μm and 1,000 μm. (**O**) Plasma levels of MPO-DNA in patients treated with or without chemotherapy (*n* = 40 and 41, respectively; Mann-Whitney test). (**P**) Quantification of NETs in untreated or radiotherapy-treated (radio) 4T1 tumors (*n* = 6/group, Mann-Whitney test). (**Q**) Quantification of neutrophil xCell score (GSE59733) from paired pre- and post-radio breast tumors (*n* = 9 pairs, paired *t* test) (22). (**R**) GSEA reveals enrichment of NET formation pathway in post-radio tumors (GSE59733) (22). Schematic illustration in **A** and **L** is created with BioRender.com. Data represent mean ± SD (**F**, **H**–**K**, and **P**) and mean ± SEM (**O**). ****P < 0.0001, ***P < 0.001, **P < 0.01, *P < 0.05.

To understand the tumor microenvironment (TME) shaped by chemotherapy at single-cell resolution, we performed a comprehensive scRNA-Seq analysis of mouse primary tumor treated with or without chemotherapy. For this, we used a well-established breast cancer model of inoculating 4T1 tumor cells into the fourth fat pads of syngeneic BALB/c mice, treating with doxorubicin (Dox) or matched vehicle when tumors were palpable, and isolating tumors after 2 dosages of drugs (day 21) (Figure 1A). Cells that passed quality control were divided into 13 independent subtypes based on the most variable genes, including neutrophils, tumor cells, CD8^+^ T cells, macrophages, CD4^+^ T cells, fibroblasts, B cells, proliferating cells, natural killer T (NKT) cells, epithelial cells, endothelial cells, plasmacytoid dendritic cells (pDCs), and plasma cells (Figure 1B and Supplemental Figure 1A; supplemental material available online with this article; https://doi.org/10.1172/JCI190557DS1). ScRNA-Seq analysis indicated that neutrophil was the most prominent immune cell population in both Dox- and vehicle-treated groups and increased significantly after Dox treatment. Besides, the proportions of CD4^+^ and CD8^+^ T cells among immune cells were obviously elevated in the Dox-treated group (Figure 1C). In line with previous reports (17), Gene Ontology (GO) pathways related to antitumor immunity were enriched in CD4^+^ and CD8^+^ T cells from Dox-treated tumors (Supplemental Figure 1B). Also, neutrophil transcriptomic signature was characterized by substantial upregulation of various biologically active signaling pathways in the Dox-treated group, including increased expression of genes involved in neutrophil migration, degranulation, activation, ROS metabolic process, and NET formation (Figure 1D). These data indicated that neutrophils might play an important role in Dox-induced pro-tumor microenvironment, matching prior reports highlighting the crucial contribution of neutrophil infiltration and NETs toward tumor progression (18, 19). Particularly, gene set enrichment analysis (GSEA) showed that the Kyoto Encyclopedia of Genes and Genomes (KEGG) pathway of NET formation was significantly enriched in the neutrophil cluster from the Dox-treated group (Figure 1E).

In the context of breast cancer, we previously observed an upregulation of NETs in metastatic liver and lung tissues, while the primary tumor contained fewer NETs (16). To further verify whether neutrophil infiltration and NET formation were enhanced by chemotherapy, we performed tissue immunofluorescence and ELISA detection of serum myeloperoxidase-DNA (MPO-DNA) complex in 4T1 tumor–bearing mice with Dox or vehicle treatments. As expected, we observed elevated Ly6G^+^ neutrophil infiltration and NET formation (costained for citrullinated histone 3 [H3cit] and MPO) in mouse primary tumors from Dox-treated group (Figure 1, F–H, and Supplemental Figure 1C). Additionally, Dox treatment also increased NET formation in metastatic lung and liver tissues and serum samples (Figure 1, H and I, and Supplemental Figure 1D). To exclude the possibility of subtype-specific effects, we also used a hormone receptor–positive (HR^+^) orthotopic MCF-7 cancer model in NOD/SCID mice and a human epidermal growth factor receptor 2–enriched (HER2-enriched), transgenic, spontaneous mouse mammary tumor virus–polyoma middle T antigen (MMTV-PyMT) mouse model on FVB/NJ background (20, 21). As expected, both tumoral Ly6G^+^ neutrophils (Supplemental Figure 1, E and F) and NET levels (Figure 1, J and K, and Supplemental Figure 1, G and H) were significantly increased by Dox treatment. Besides, we found that NET formation in lung and liver metastasis was also enhanced after Dox treatment in MMTV-PyMT FVB/NJ mice (Supplemental Figure 1, I and J). We also employed paired pretreatment biopsy samples and posttreatment resected tumor samples from 81 patients with breast cancer, who had received neoadjuvant chemotherapy (NAC), and examined NET expression (Figure 1L). In line with the results from mouse models, NET expression in paired patient primary tumor was significantly upregulated after chemotherapy (Figure 1, M and N). This was further verified by the relatively higher levels of plasma NETs in patients who underwent NAC, compared with those without chemotherapy before surgery (Figure 1O).

Also, local radiotherapy conducted in the mouse breast cancer model increased neutrophil accumulation and NET expression in primary tumor (Figure 1P and Supplemental Figure 2, A–C). This phenomenon was further verified in paired tumor RNA-Seq data from patients with breast cancer (22), showing increased neutrophil score and enriched NET formation pathway after radiotherapy (Figure 1, Q and R). As radiotherapy is crucial in lung cancer treatment, we also analyzed public scRNA-Seq data of Lewis’s lung cancer (23) and found that both neutrophil proportion and NETosis pathway were enhanced after radiotherapy (RT04 group) (Supplemental Figure 2, D–F). Collectively, these results suggest that chemotherapy and radiotherapy could promote NET formation in breast cancer.

### Enhanced NET generation impairs the efficacy of chemotherapy and radiotherapy.

**Figure 2:**
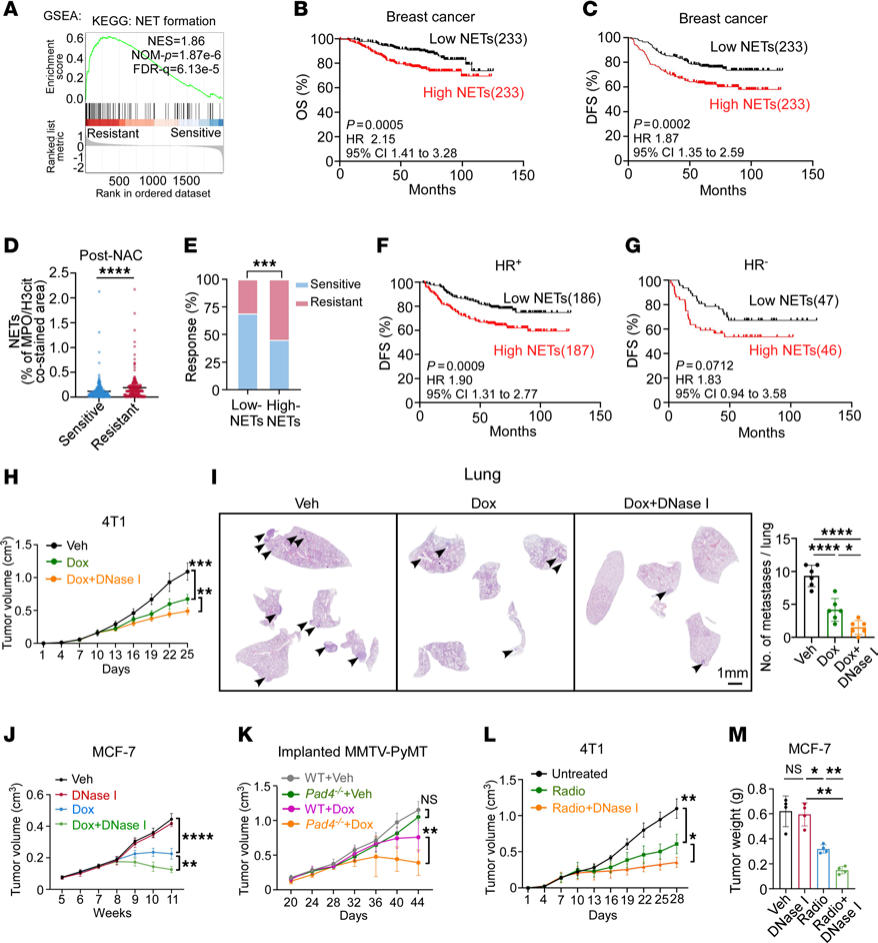
Enhanced NET generation impairs the efficacy of chemotherapy and radiotherapy in breast cancer. (**A**) GSEA reveals NET formation pathway enrichment in resistant breast cancer (GSE18728) (24). (**B** and **C**) OS (**B**) and DFS (**C**) of patients containing low and high NETs (*n* = 233/group, log-rank test). HR, hazard ratio; CI, confidence interval. (**D**) Quantification of NETs in post-NAC breast tumors from patients sensitive or resistant to NAC (*n* = 265 and 201, respectively; Mann-Whitney test). (**E**) Response rates in patients with low or high NETs (Fisher’s exact test). (**F** and **G**) DFS of HR^+^ (**F**) and HR^–^ (**G**) patients containing low (HR^+^: *n* = 186, HR^–^: 47) and high (HR^+^: *n* = 187, HR^–^: 46) NETs (log-rank test). (**H** and **I**) Tumor growth (**H**) and lung H&E staining in 4T1-bearing mice (*n* = 6/group; **H**, 2-way repeated measures ANOVA with Tukey’s test; **I**, 1-way ANOVA with Tukey’s test). (**J**–**L**) Tumor growth in MCF-7–bearing mice (*n* = 5/group) (**J**), MMTV-PyMT-bearing wild-type (WT) or *Pad4*^–/–^ mice (*n* = 7–9/group) (**K**), and 4T1-bearing mice (*n* = 4/group) (**L**) (2-way repeated measures ANOVA with Tukey’s test). (**M**) MCF-7 tumor weight (*n* = 4/group, Welch’s ANOVA with Dunnett’s T3 test). Data represent mean ± SD (**H**–**M**) and mean ± SEM in **D**. ****P < 0.0001, ***P < 0.001, **P < 0.01, *P < 0.05.

To investigate whether chemotherapy-induced NETs contributed to therapeutic resistance, we first screened for the enriched KEGG pathways in resistant post-NAC breast tumor samples from a public dataset (24) (Figure 2A and Supplemental Figure 3A). We observed the NET formation among the upregulated pathways. To further verify this phenomenon, we conducted prognosis analysis in an independent breast cancer patient cohort who had undergone NAC. Using the median value of our cohort as a cutoff, we stratified patients into 2 groups based on tumoral NET expression levels: high (above median) or low (below median). We found that high NET expression in primary tumor was associated with shorter patient survival, demonstrated by both the overall survival (OS) and disease-free survival (DFS) (Figure 2, B and C). We then explored the impact of NET expression level and other baseline clinical variables on DFS and OS in the same patient cohort using the Cox regression model. Significantly, univariate analysis confirmed that high NET level or advanced clinical stage was strongly correlated with worse OS and DFS, while positive HR status was associated with improved OS and DFS. Besides, multivariable analysis, which adjusted for potential confounding factors like HR status and clinical stage, further confirmed that high NET levels were closely associated with shorter OS and DFS, suggesting that NETs were independently associated with poor prognosis in our study (Supplemental Table 1). Besides, NETs were significantly higher in patients who were refractory to NAC, compared with those who were sensitive (Figure 2D). Also, patients with high tumoral NET levels had worse therapeutic response than those with low NET levels (Figure 2E).

To clarify whether the clinical significance varies with the characteristics of patients with breast cancer, we further grouped the patients by pathologic subtypes and evaluated prognosis data in different groups. Interestingly, high NET level was closely associated with worse survival in patients with HR^+^ subtypes, demonstrated by both OS and DFS (Figure 2F and Supplemental Figure 3B). On the other hand, in patients with HR^–^ breast cancer, a trend of shorter survival was also observed in those with high levels of NETs, though the statistical difference was not significant for either DFS or OS (Figure 2G and Supplemental Figure 3B).

To explore the role of NETs in chemotherapy resistance in vivo, we constructed orthotopic 4T1 and MCF-7 tumor models, as well as transgenic MMTV-PyMT FVB/NJ mice, and treated mice with Dox in the presence or absence of DNase I (a nuclease that degrades NET-DNA) (25). In 4T1 tumor models and MMTV-PyMT FVB/NJ mice, we observed that Dox combined with DNase I more significantly reduced both tumor growth and lung or liver metastasis compared with Dox or DNase I monotherapy (Figure 2, H and I, and Supplemental Figure 3, C–E). In the MCF-7 tumor model, which barely metastasizes to lung and liver (26), we also found that tumor volume and weight in mice from the combination of Dox and DNase I group were the lowest (Figure 2J and Supplemental Figure 3F). Additionally, we inoculated fresh tumor cells isolated from spontaneous mammary tumors of MMTV-PyMT mice to wild-type C57BL/6J (WT) or syngeneic peptidylarginine deiminase 4–knockout (*Pad4^–/–^*) mice (the latter showed substantially impaired capacity to generate NETs) (16), then treated them with Dox or vehicle. Consistent with the results in DNase I treatment mouse models, Dox treatment more significantly decreased the tumor volume of *Pad4^–/–^* mice compared with WT mice (Figure 2K). In parallel, tumoral NET expression was remarkably decreased in DNase I–treated or *Pad4*^–*/*–^ mice (Supplemental Figure 4, A–C). Moreover, in 4T1 and MCF-7 tumor models, radiotherapy combined with DNase I treatment more significantly suppressed tumor growth and lung metastasis (4T1 tumor model) than radiation or DNase I treatment alone (Figure 2, L and M, and Supplemental Figure 4D) and degraded radiotherapy-induced tumoral NET formation (Supplemental Figure 4E). Together, these data suggest that targeting NET-DNA induced by chemotherapy or radiotherapy, either through DNase I or in *Pad4*^–*/*–^ mice, could reduce primary breast cancer growth as well as lung and liver metastasis.

### Macrophage-derived CXCL1/2 and CFB promote neutrophil infiltration and NETosis under chemotherapy and radiotherapy.

**Figure 3:**
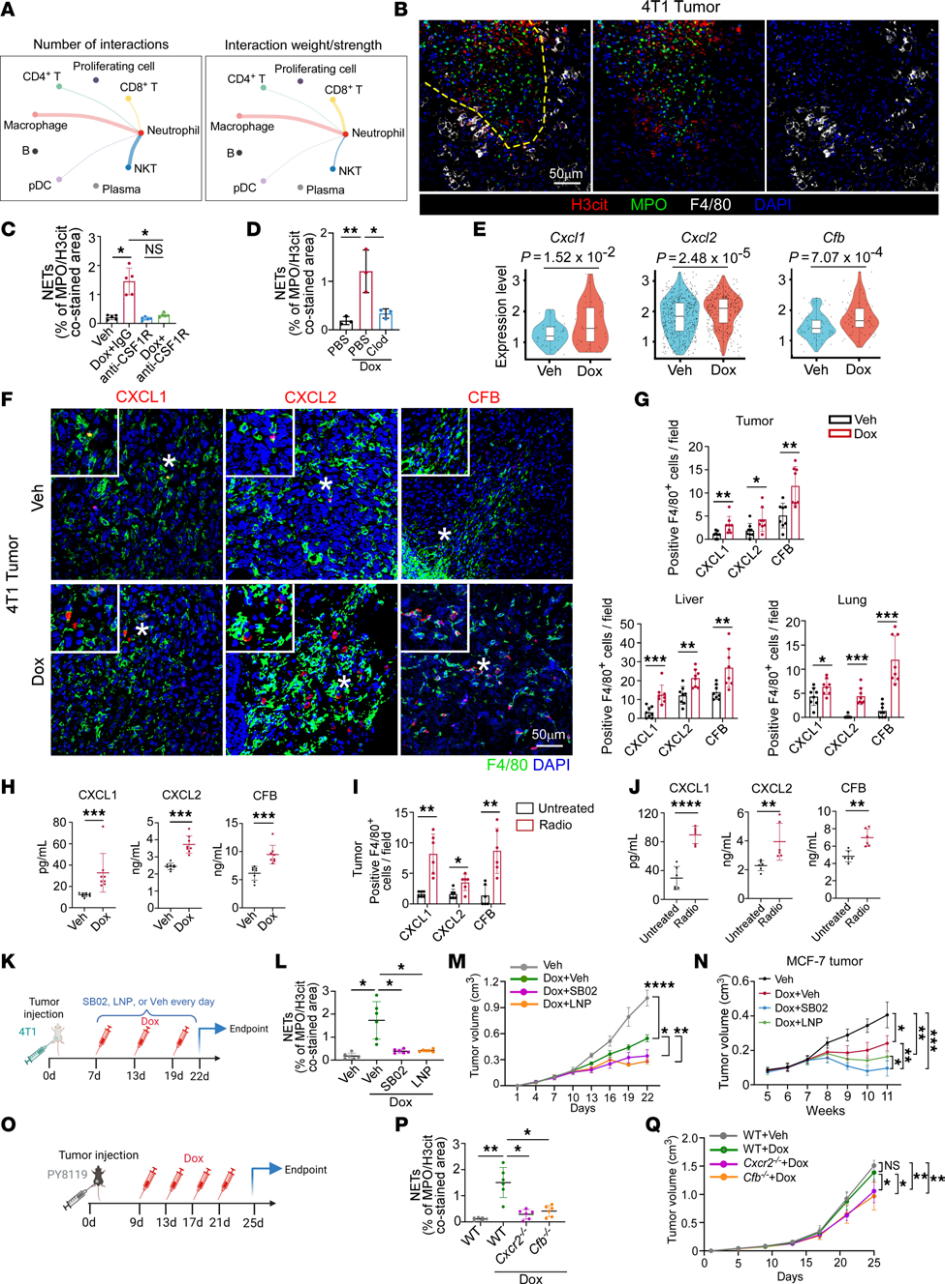
Macrophage-derived CXCL1/2 and CFB promote neutrophil infiltration and NETosis under chemotherapy and radiotherapy. (**A**) Circle plots showing the interaction number (left) and weight/strength (right) between neutrophils and other immune cells in scRNA-Seq. (**B**) Representative immunofluorescence images in Dox-treated tumors. (**C** and **D**) Quantification of tumoral NETs (**C**, *n* = 5/group, Welch’s ANOVA with Dunnett’s T3 test; **D**, *n* = 3/group, 1-way ANOVA with Tukey’s test). Clod, clodronate liposome. (**E**) Violin plots showing *Cxcl1/2* and *Cfb* expression in macrophages (Welch’s *t* test). Dots represent individual cells. Box bounds indicate the 25th and 75th percentiles and middle lines the median. Whiskers represent interquartile range. (**F** and **G**) Representative tumoral immunofluorescence images (**F**) and quantification in tumors, livers, and lungs (*n* = 8/group) (**G**). For tumor CXCL1 and CFB, liver CFB, lung CXCL2 and CFB, Mann-Whitney test. For tumor CXCL2, liver CXCL1/2, and lung CXCL1, unpaired *t* test. (**H**) Serum levels of CXCL1/2 and CFB (*n* = 8/group, Mann-Whitney test). (**I**) Quantification of CXCL1/2^+^F4/80^+^ or CFB^+^F4/80^+^ cells in 4T1 tumors (*n* = 6/group). CXCL1, Mann-Whitney test. CXCL2 and CFB, unpaired *t* test. (**J**) Serum levels of CXCL1/2 and CFB (*n* = 6/group). CXCL1 and CFB, unpaired *t* test. CXCL2, Mann-Whitney test. (**K**–**M**) Experimental schematic (**K**), quantification of NETs (**L**), and tumor growth curves (**M**) (*n* = 6/group; **L**, Welch’s ANOVA with Dunnett’s T3 test; **M**, 2-way repeated measures ANOVA with Tukey’s test). SB02, SB225002. LNP, LNP023. (**N**) MCF-7 tumor growth curves (*n* = 5/group, 2-way repeated measures ANOVA with Tukey’s test). (**O**–**Q**) WT, *Cxcr2*^–/–^, and *Cfb*^–/–^ mice inoculated with PY8119 cells with indicated treatments (*n* = 6/group). (**O**) Experimental schematic. (**P**) Quantification of NETs (Welch’s ANOVA with Dunnett’s T3 test). (**Q**) Tumor growth curves (2-way repeated measures ANOVA with Tukey’s test). Schematic illustration in **K** and **O** is created with BioRender.com. Data represent mean ± SD. Scale bars, 50 μm. ****P < 0.0001, ***P < 0.001, **P < 0.01, *P < 0.05.

Next, we sought to explore how neutrophil migration and NET generation were induced by anticancer therapeutics. Using Cellchat analysis, we investigated the cell-cell interaction in scRNA-Seq of mouse 4T1 tumors and focused on the cell type that exhibited the most proximity with neutrophils. Notably, the macrophage subtype showed stronger interaction with neutrophils, compared with other subtypes (Figure 3A and Supplemental Figure 5A). In primary tumors from patients and mice under chemotherapy, we also observed that neutrophil aggregation and NETs were frequently confined to the macrophage-rich region (Figure 3B and Supplemental Figure 5B). Collectively, these data suggest that macrophages might be a critical contributor to neutrophil migration and NETosis after chemotherapy.

To further evaluate the importance of macrophages in NETosis in vivo, we removed mouse macrophages using anti–colony-stimulating factor 1 receptor (anti-CSF1R) monoclonal antibody (mAb) (27) or clodronate liposomes (28) and detected neutrophil infiltration and NET generation induced by Dox administration (Supplemental Figure 5, C–F). As anticipated, macrophage removal significantly reduced Dox-induced Ly6G^+^ neutrophil infiltration and NET formation, implying macrophage-derived factors induced neutrophil migration and NETosis (Figure 3, C and D, and Supplemental Figure 5, G–K). Previous studies have reported that a series of cytokines and complement activation products C3a and C5a are intensely involved in neutrophil migration and NETosis (18). Therefore, we searched for potential cytokines or complement factors reported to be associated with neutrophil migration and NETosis in scRNA-Seq of 4T1 tumors. Among them, we found that expression of *Cxcl1/2* and *Cfb* was significantly upregulated in macrophages derived from Dox-treated tumors versus those treated with vehicle (Figure 3E and Supplemental Figure 5, L–N). Next, we confirmed that macrophage-derived CXCL1/2 and CFB were upregulated in tumors, liver and lung tissues, as well as serum from mice orthotopically inoculated with 4T1 breast cancer cells after Dox treatment by immunofluorescence staining (Figure 3, F and G, and Supplemental Figure 6A) and ELISAs (Figure 3H). This was also verified by orthotopic MCF-7 cancer model and spontaneous MMTV-PyMT FVB/NJ mouse model, where Dox treatment obviously stimulated the production of CXCL1/2 and CFB from tumoral macrophages (Supplemental Figure 6, B and C). Indeed, macrophage depletion via anti-CSF1R mAb or clodronate liposomes significantly decreased the number of cells expressing CXCL1/2 and CFB in tumor (Supplemental Figure 6, D and E), as well as the levels of C3a and C5a (Supplemental Figure 5K). Moreover, in line with the results of chemotherapy, the expression of CXCL1/2 and CFB in macrophages was significantly increased in response to local radiation, evidenced by data from immunofluorescence (Figure 3I and Supplemental Figure 6F), Western blot (Supplemental Figure 6G), scRNA-Seq (Supplemental Figure 6H), and ELISA (Figure 3J) in mouse cancer models.

To evaluate the importance of CXCL1/2 and CFB in driving NET formation under treatments, we applied Dox in combination with SB225002 or LNP023, the inhibitors of CXCR2 (the co-receptor of CXCL1/2) or CFB, respectively, in orthotopic 4T1 and MCF-7 tumor model (Figure 3K and Supplemental Figure 7A) (29, 30). Our data indicated that blockade of CXCR2 or CFB efficiently prohibited Dox-induced NET generation (Figure 3L and Supplemental Figure 7, B–E) and enhanced chemotherapy effects, leading to markedly decreased tumor growth (Figure 3, M and N). We also constructed *Cxcr2*- and *Cfb*-knockout (*Cxcr2*^–*/*–^ and *Cfb*^–*/*–^) mice and implanted mouse breast cancer cells into the fourth fat pads, followed by Dox administration (Figure 3O). In agreement, Dox also exhibited better therapeutic efficacy in *Cxcr2*^–*/*–^ and *Cfb*^–*/*–^ mice with reduced tumor growth and decreased tumoral NET level (Figure 3, P and Q, and Supplemental Figure 7F). After demonstrating the elevated production of CXCL1/2 and CFB by macrophages in lung and liver metastatic niches (Figure 3G and Supplemental Figure 6A), we next sought to validate their contribution to NET formation in metastatic organs and exacerbation of metastatic progression. To exclude the confounding effects induced by primary tumor, we constructed lung or liver experimental metastasis models by injecting 4T1 cells via tail vein or EO771 cells intrasplenically and applied Dox with or without CXCR2 or CFB inhibition. Consistently, both NET formation and lung or liver metastatic nodules were remarkably reduced when CXCR2 and CFB were blocked or knocked out (Supplemental Figure 7, G–L). Furthermore, in orthotopic EO771 tumor model, we demonstrated that primary tumors from *Cxcr2*^–*/*–^ and *Cfb*^–*/*–^ mice were also smaller after radiotherapy compared with WT mice, with a significant suppression of serum NET level (Supplemental Figure 7, M and N). Together, these data suggest that macrophage-derived CXCL1/2 and CFB serve as key drivers of neutrophil aggregation and NET formation in breast cancer following chemotherapy and radiotherapy.

### Phagocytosis of tumor debris by macrophages contributes to CXCL1/2 and CFB elevation and NETosis.

**Figure 4:**
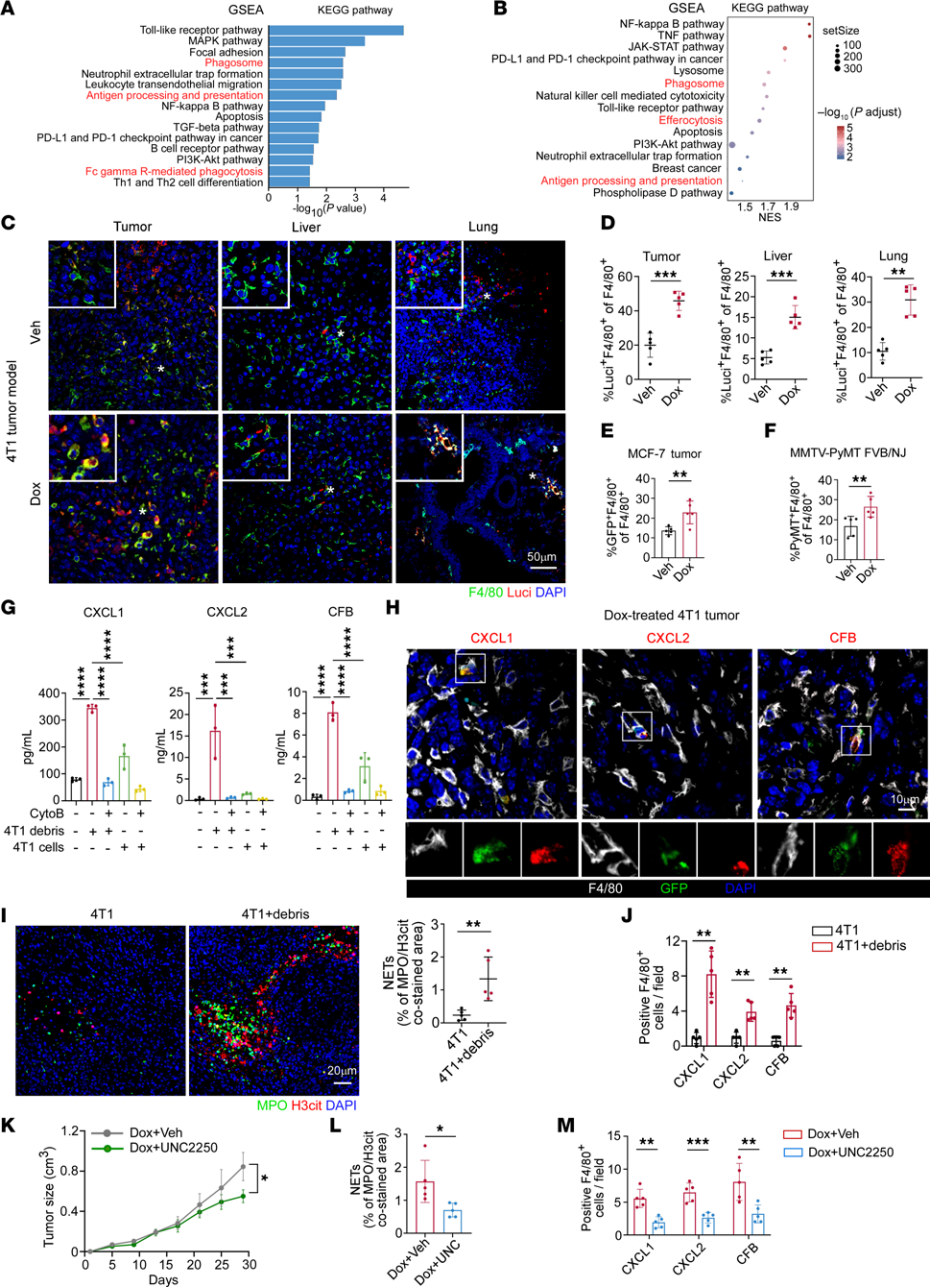
Macrophages promote CXCL1/2 and CFB elevation and NETosis via phagocytosis of treatment-induced tumor debris. (**A**) GSEA showing functional enrichment of KEGG pathways in macrophage subset from scRNA-Seq of 4T1 tumors (permutation test). (**B**) GSEA showing functional enrichment of KEGG pathways in RNA-Seq of patient breast tumors post-chemotherapy versus pretreatment (GSE18728; pre: *n* = 21, post: *n* = 22; permutation test). (**C** and **D**) Representative immunofluorescence images (**C**) and quantification of the proportions of Luci^+^F4/80^+^ cells (**D**) in tumors, livers, and lungs (*n* = 5/group). Luci, luciferase. * indicates areas magnified in the top-left insets. Tumor and liver, unpaired *t* test. Lung, Mann-Whitney test. (**E**) Quantification of the proportions of GFP^+^F4/80^+^ cells in MCF-7 tumors (*n* = 5/ group, unpaired *t* test). (**F**) Quantification of the proportions of tumoral PyMT^+^F4/80^+^ cells in MMTV-PyMT FVB/NJ mice (*n* = 5/group, Mann-Whitney test). (**G**) ELISA levels of CXCL1/2 and CFB in culture medium of BMDMs with indicated treatments (*n* = 3/group, 1-way ANOVA with Tukey’s test). (**H**) Representative immunofluorescence images in 4T1 tumors after chemotherapy. White boxes indicate areas magnified in the next row. Data represent 3 independent experiments. (**I** and **J**) 4T1 cells were injected alone or coinjected with 4T1 debris orthotopically in BALB/c mice (*n* = 5/group). (**I**) Representative images and quantification of tumoral NETs. (**J**) Quantification of tumoral CXCL1^+^F4/80^+^, CXCL2^+^F4/80^+^, and CFB^+^F4/80^+^ cells (Mann-Whitney test). Scale bar, 20 μm. (**K**–**M**) Orthotopic 4T1 tumors treated with Dox together with UNC2250 or vehicle (*n* = 5/group). (**K**) Tumor growth curves. (**L**) Quantification of NETs. (**M**) Quantification of CXCL1^+^F4/80^+^, CXCL2^+^F4/80^+^, and CFB^+^F4/80^+^ cell counts. Data were analyzed by 2-way repeated measures ANOVA with Šídák’s test and compared on day 29 (**K**), or unpaired *t* test (**L** and **M**). Data represent mean ± SD. ****P < 0.0001, ***P < 0.001, **P < 0.01, *P < 0.05.

As we showed that macrophages mainly contributed to NETosis postchemotherapy, we next set out to determine the functional impact of chemotherapy on macrophages. Dramatically, in scRNA-Seq data of 4T1 tumors, GSEA revealed that macrophages from Dox-treated 4T1 tumors exhibited increased expression of genes associated with KEGG pathways, including phagosome, antigen processing and presentation, and Fc gamma R-mediated phagocytosis, predicting their potentially enhanced phagocytic activity (Figure 4A). This result was consistent with a public RNA-Seq dataset of tumors from patients with breast cancer postchemotherapy in comparison with prechemotherapy, where postchemotherapy tumors showed enriched phagocytosis-associated pathways (24) (Figure 4B). We also observed that phagocytosis- and efferocytosis-associated pathways were significantly enriched in macrophages after radiotherapy (Supplemental Figure 8A), which was also confirmed by another external dataset (31) (Supplemental Figure 8B). Previous studies have demonstrated that anticancer therapies could cause increased damage to tumor cells and induce the production of tumor debris, subsequently reprogramming the TME and paradoxically facilitating tumor progression (32, 33). Besides, it is widely accepted that macrophages possess stronger phagocytic capability compared with other cell subtypes (34). Therefore, based on the possibly increased phagocytic ability of macrophages after chemotherapy and radiotherapy, we sought to investigate whether the engulfment of tumor debris was enhanced under treatment. Using luciferase (Luci), green fluorescent protein (GFP), or PyMT as a surrogate for tumor cells, we observed that the proportions of Luci^+^F4/80^+^ cells, GFP^+^F4/80^+^ cells, and PyMT^+^F4/80^+^ cells (which represent macrophages with phagocytosed tumor debris) in total F4/80^+^ macrophages were remarkably upregulated after chemotherapy in tumors and metastatic organs (Figure 4, C–F, and Supplemental Figure 8C). Moreover, local radiotherapy also resulted in obviously increased proportions of macrophages that had engulfed tumor debris in 4T1 tumors (Supplemental Figure 8D).

To further evaluate the impact of macrophage-mediated debris engulfment on the upregulation of CXCL1/2 and CFB, we suppressed phagocytic capability of macrophages in vitro using an inhibitor of actin polymerization, cytochalasin B (CytoB) (35). The results of ELISA and qRT-PCR assay revealed that tumor debris stimulation and engulfment remarkably elevated the expression of CXCL1/2 and CFB in mouse bone marrow–derived macrophages (BMDMs), whereas intact tumor cell treatment exerted less effect on CXCL1/2 or CFB production (Figure 4G and Supplemental Figure 8, E and F). In contrast, CytoB reduced phagocytosis of macrophages cocultured with tumor debris (Supplemental Figure 8, G and H) and decreased the induction of CXCL1/2 and CFB expression in macrophages (Figure 4G and Supplemental Figure 8F). In addition, we employed UNC2250, an inhibitor of tyrosine protein kinase Mer (MerTK), which was one of the most critical phagocytic receptors on macrophages (36). As expected, pretreatment of human peripheral monocyte-derived macrophages with UNC2250 decreased tumor cell debris–induced elevation of *CXCL1/2* and *CFB* (Supplemental Figure 8I), suggesting that increased engulfment of tumor debris promoted CXCL1/2 and CFB production.

To test these findings in vivo, we constructed a tumor xenograft model with GFP^+^ tumor cells, followed by administration of Dox. The expression of CXCL1/2 or CFB was markedly elevated in macrophages that had engulfed GFP^+^ tumor (Figure 4H). To further evaluate the effects of tumor debris, we isolated Dox-induced 4T1 cell debris in vitro and coinjected it with live 4T1 cells into BALB/c mice as previously described (32). Of note, coinjection of 4T1 cells with their debris enhanced tumoral NETosis (Figure 4I) and the expression of CXCL1/2 and CFB (Figure 4J) in comparison with 4T1 cell injection alone. Similar to in vitro experiments, mice were also treated with UNC2250 for 16 consecutive days since chemotherapy began, and then tumors were isolated for examination of phagocytosis efficacy and NET level. As expected, macrophage engulfment of debris, tumor growth, NETosis, and CXCL1/2 and CFB expression were obviously reduced in the presence of UNC2250 (Figure 4, K–M, and Supplemental Figure 8J). These findings suggest that tumor debris induced by anticancer therapies could promote the expression of CXCL1/2 and CFB in macrophages, which induce NETosis.

### The protein CTSC in tumor debris induces CXCL1/2 and CFB expression.

**Figure 5:**
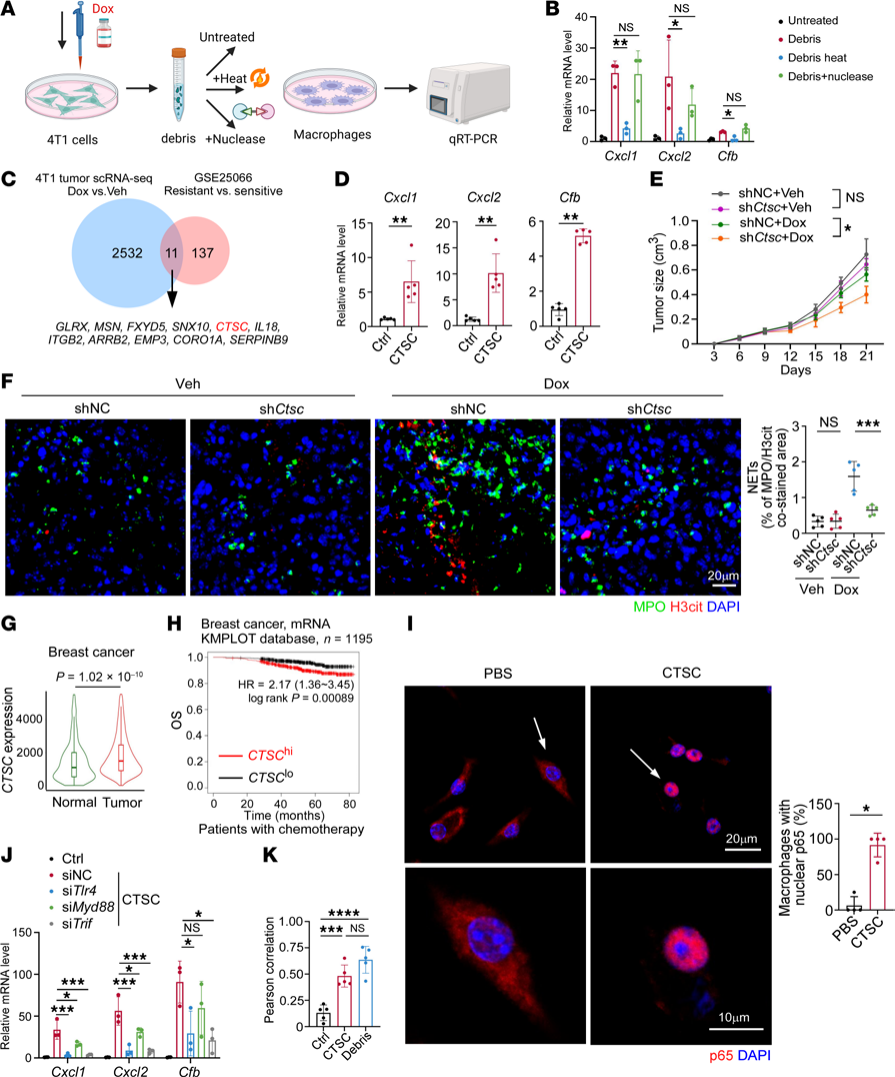
The protein CTSC in tumor debris contributes to NETosis after chemotherapy. (**A**) Schematic showing qRT-PCR analysis of BMDMs stimulated with Dox-induced 4T1 debris with different pretreatments. Created with BioRender.com. (**B**) *Cxcl1/2* and *Cfb* mRNA expression in BMDMs (*n* = 3/group, 1-way ANOVA with Tukey’s test). (**C**) Venn diagram showing the overlapping DEGs upregulated in scRNA-Seq of 4T1 tumors and RNA-Seq (GSE25066). (**D**) *Cxcl1/2* and *Cfb* mRNA expression in BMDMs treated with or without recombinant CTSC protein (*n* = 5/group, Mann-Whitney test). (**E** and **F**) Tumor growth curves (**E**), representative NET images, and quantification (**F**) of 4T1-shNC or 4T1-sh*Ctsc* tumors with indicated treatments (*n* = 5/group). Scale bar, 20 μm. Data were analyzed by 2-way repeated measures ANOVA with Tukey’s test and compared at day 21 (**E**), or 2-way ANOVA with Tukey’s test (**F**). (**G**) Violin plot showing *CTSC* expression in breast tumors and normal breast tissues from the TNMplot database (normal: *n* = 242; tumor: *n* = 7,569; Mann-Whitney test). The lower and upper bounds of boxes indicate the 25th and 75th percentiles, and the middle lines the median values. The whiskers represent the interquartile range. (**H**) Kaplan-Meier curves for OS in substratified patients with breast cancer receiving chemotherapy with high (*n* = 617) and low (*n* = 578) *CTSC* expression from the KMPLOT database (log-rank test). (**I**) BMDMs were treated with PBS or recombinant CTSC protein. Immunostaining of p65 and quantification are shown (*n* = 4/group, Mann-Whitney test). The white arrows indicate the areas that are magnified in the next row. Scale bars, 20 μm and 10 μm. (**J**) *Cxcl1/2* and *Cfb* mRNA expression in BMDMs with indicated treatments (*n* = 3/group, 1-way ANOVA with Tukey’s test). (**K**) Quantification of TLR4/RAB5 colocalization in BMDMs (*n* = 5/group, 1-way ANOVA with Tukey’s test). Data represent mean ± SD. ****P < 0.0001, ***P < 0.001, **P < 0.01, *P < 0.05.

To investigate the specific component within tumor debris that played a main role in eliciting macrophage expression of CXCL1/2 and CFB, heat inactivation or nuclease treatment was performed to diminish protein or nucleic acid components, respectively, in vitro (Figure 5A). Of note, heat denaturation, rather than nuclease application, more significantly reduced the production of *Cxcl1/2* and *Cfb* mRNA induced by tumor debris, indicating that proteins from tumor debris were more critically involved (Figure 5B).

After showing the close association between NET formation and chemotherapy resistance in breast cancer, we analyzed the transcriptionally upregulated genes of chemoresistant tumors versus chemosensitive tumors in RNA-Seq data from a public dataset (37) (Figure 5C). We also searched for the upregulated genes in the tumor cell subcluster from our scRNA-Seq of 4T1 tumors with Dox or vehicle treatments (Figure 5C). Overlapping analysis revealed 11 genes that were consistently upregulated in the 2 datasets, among which *CTSC* was specifically noticed for its reported association with NETosis (38) (Figure 5C). To exclude the possibility that other proteins may function in debris-induced cytokine expression, we employed siRNA-mediated knockdown of several proteins, including *Ctsc*, high mobility group box 1 (*Hmgb1*), S100 calcium binding protein A9 (*S100a9*), translationally controlled tumor protein (*Tctp*), and heat shock protein70 (*Hsp70*), which had been reported to induce CXCL1/2 or CFB production (39–43). As expected, the expression of debris-induced CXCL1/2 and CFB was most obviously decreased after targeting *Ctsc*, compared with other targeted proteins (Supplemental Figure 9A). Besides, stimulating macrophages with recombinant CTSC protein in vitro increased the expression of *Cxcl1/2* and *Cfb* (Figure 5D).

To further validate the function of CTSC in vivo, we transfected 4T1 cells with lentiviruses knocking down *Ctsc* or the negative control (NC) and orthotopically injected them into mice (Supplemental Figure 9B). After chemotherapy application, we found 4T1 tumors with CTSC knockdown (sh*Ctsc*) grew much slower than those transfected with NC lentiviruses (shNC) (Figure 5E), and tumoral NET expression was inhibited in accordance (Figure 5F), suggesting CTSC in tumor debris worked as an important contributor to NETosis in postchemotherapy breast tumor. Using the TNMplot database (44), we observed that tumoral *CTSC* expression was significantly higher than adjacent normal tissues in patients with breast and colon cancer (Figure 5G and Supplemental Figure 9C). We next utilized the Kaplan-Meier plotter website to confirm the impact of *CTSC* expression on patient prognosis (45). Obviously, patients with higher *CTSC* level exhibited significantly shorter survival, validated by 2 independent cohorts of patients with breast and colon cancer who had received chemotherapy (Figure 5H and Supplemental Figure 9D). Collectively, these data demonstrate that CTSC promotes NETosis in tumor, impairs chemotherapy efficacy, and predicts poor patient outcomes.

We next sought to investigate the underlying mechanism of CTSC in inducing CXCL1/2 and CFB expression. We found that inhibitors targeting enzyme activity of cathepsins (38, 46) exerted negligible influence on *Cxcl1/2* and *Cfb* levels (Supplemental Figure 9E), suggesting that CXCL1/2 and CFB upregulation was not attributed to enzyme activity of CTSC. To investigate the underlying mechanism, we performed RNA-Seq on mouse macrophages treated with PBS control or tumor debris (Supplemental Figure 9F). Particularly, we noticed that a series of GO pathways associated with TLR activation were enriched in debris-treated macrophages in our RNA-Seq data (Supplemental Figure 9G). As reported by numerous studies, ligands binding to TLRs often activated downstream NF-κB through myeloid differentiation factor 88 (MyD88) or TIR domain–containing adaptor inducing interferon-β (TRIF) adaptors (47, 48). As NF-κB was the main transcriptional factor responsible for the expression of CXCL1/2 and CFB (49–51), we treated macrophages with recombinant CTSC and detected the nuclear translocation of p65 via immunofluorescence. As expected, CTSC coculturing elicited increased levels of intranuclear p65 (Figure 5I), suggesting the activation of NF-κB signaling. In parallel, we observed that blockade of the NF-κB pathway with inhibitors JSH-23 or Sc-3060 decreased *Cxcl1/2* and *Cfb* expression induced by recombinant CTSC in vitro (Supplemental Figure 9H). The cathepsins were reported to bind and activate TLR4-mediated pathways (52, 53). Thus, we used siRNA-dependent silencing of *Tlr4*, *Myd88*, and *Trif* in macrophages to assess whether TLR4 was the receptor of CTSC. Significantly, the CTSC-induced *Cxcl1/2* and *Cfb* were more obviously decreased after silencing *Tlr4* or *Trif* in macrophages, while *Myd88* knockdown elicited relatively less impact (Figure 5J). Besides, with the addition of recombinant CTSC or tumor debris, TLR4 was colocalized with RAB5 (the indicator of early endosome) in macrophages (Figure 5K and Supplemental Figure 9I), consistent with the point that TRIF adaptor was preferentially activated in endosomes (54). Collectively, our findings suggest that CTSC promotes CXCL1/2 and CFB expression in macrophages via activating the TLR4/TRIF/NF-κB pathway.

### NETs promote therapeutic resistance of breast cancer via CCDC25.

**Figure 6:**
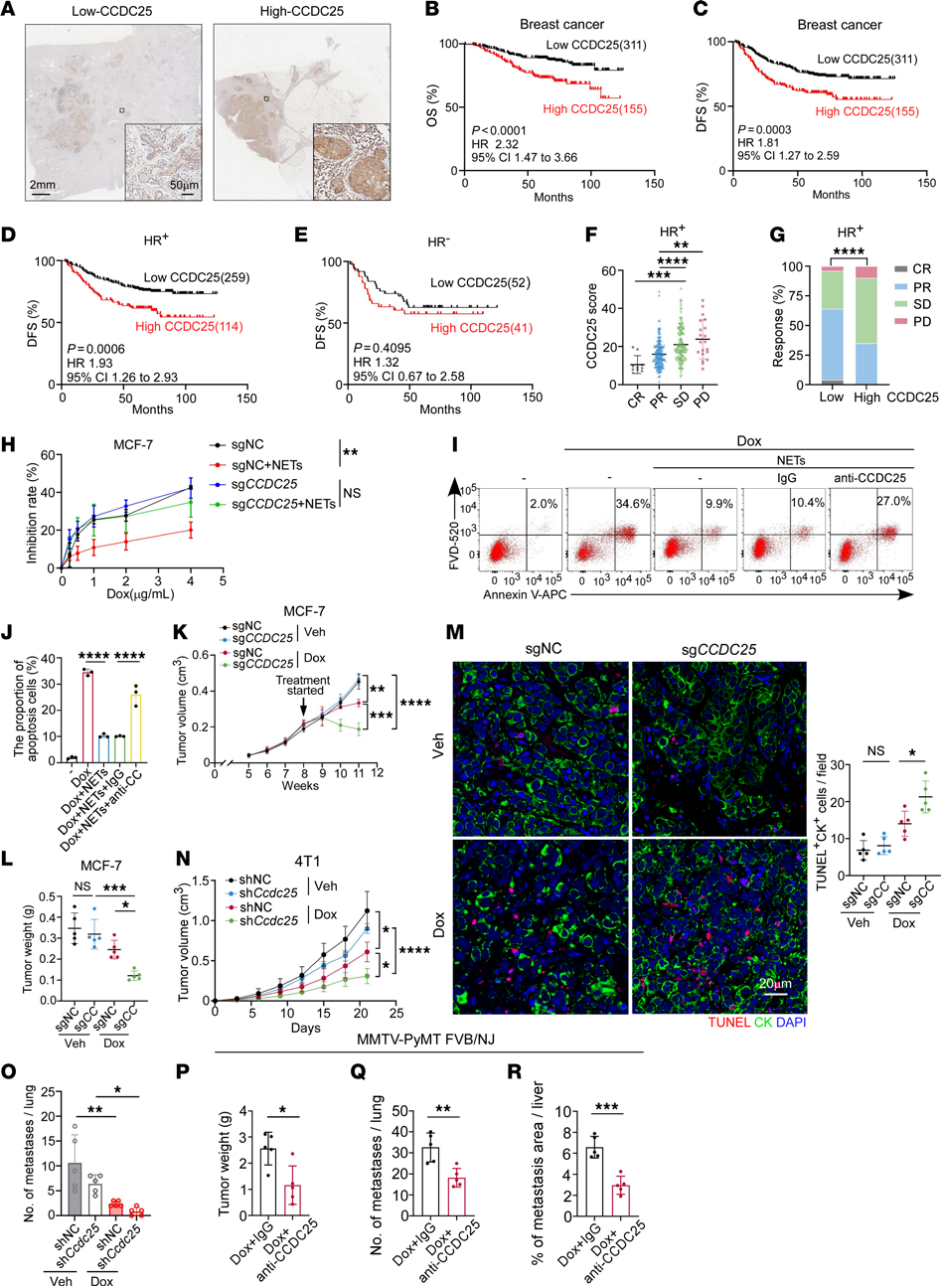
NETs promote therapeutic resistance of breast cancer via CCDC25. (**A**) Representative images showing CCDC25 expression in breast cancer (*n* = 466). Black boxes indicate areas that are magnified in bottom-right corner insets. Scale bars, 2 mm and 50 μm. (**B** and **C**) OS (**B**) and DFS (**C**) of patients with low and high CCDC25 (*n* = 311 or 155, respectively; log-rank test). (**D** and **E**) Kaplan-Meier curves revealing DFS for HR^+^ (**D**) and HR^–^ (**E**) patients with low (HR^+^: *n* = 259, HR^–^: 52) and high (HR^+^: *n* = 114, HR^–^: 41) CCDC25 (log-rank test). (**F**) Quantification of CCDC25 expression in HR^+^ tumors post-NAC with different therapeutic responses (Kruskal-Wallis test followed by Dunn’s test). CR, complete response (*n* = 10); PR, partial response (*n* = 196); SD, stable disease (*n* = 147); PD, progressive disease (*n* = 20). (**G**) Response rates of NAC in HR^+^ breast cancer with high or low CCDC25 (χ^2^ test). (**H**) Growth inhibition rates of Dox on sgNC or sgCCDC25 MCF-7 cells (*n* = 3/group, 2-way ANOVA with Šídák’s test). sg, single-guide. (**I** and **J**) The proportions of annexin V^+^FVD^–^ (early apoptosis) and annexin V^+^FVD^+^ (late apoptosis) cells (**I**) and quantification (**J**) (*n* = 3/group, 1-way ANOVA with Tukey’s test). (**K**–**M**) Tumor growth curves (**K**), tumor weight (**L**), and representative images and quantification of TUNEL^+^CK^+^ cells (**M**) in MCF-7–bearing mice (*n* = 5/group; **K**, 2-way repeated measures ANOVA with Tukey’s test; **L** and **M**, 2-way ANOVA with Tukey’s test). (**N** and **O**) Tumor growth (**N**) and quantification of lung metastases (**O**) in 4T1-bearing mice (*n* = 5/group; **N**, 2-way repeated measures ANOVA with Tukey’s test; **O**, 2-way ANOVA with Tukey’s test). Scale bar, 20 μm. (**P**–**R**) Tumor weight (**P**), lung metastases (**Q**), and liver metastasis (**R**) in MMTV-PyMT FVB/NJ mice (*n* = 5/group, unpaired *t* test). Data represent mean ± SEM (**F**) and mean ± SD (**H** and **J**–**R**). ****P < 0.0001, ***P < 0.001, **P < 0.01, *P < 0.05.

Having recognized the detrimental effect of NETs on chemotherapy response and tumor progression, we aimed to explore the underlying mechanism and identify a clinically druggable target. We recently identified CCDC25 as a NET-DNA sensor that is overexpressed on the cancer cell membrane and promotes cancer metastasis (16). Thus, we investigated whether CCDC25 played a role in NET-mediated chemoresistance. Clinically, in our cohort of patients with breast cancer receiving NAC, high tumoral expression of CCDC25 was correlated with reduced long-term survival (Figure 6, A–C), and this was corroborated by 2 online databases (45, 55) (Supplemental Figure 10, A and B). In addition, we observed that high CCDC25 level was significantly associated with shorter DFS and OS in the patients with HR^+^ subtypes but not in the patients with HR^–^ breast cancer (Figure 6, D and E, and Supplemental Figure 10, C and D). To further ascertain whether CCDC25 correlates with chemotherapy response in HR^+^ patients, we stratified patients depending on their response to chemotherapy and analyzed the tumoral intensity of CCDC25 in different groups. Consistently, CCDC25 expression was inversely associated with chemotherapeutic efficacy in HR^+^ patients (Figure 6F). Additionally, we found HR^+^ patients with low CCDC25 levels had better therapeutic outcomes than those with high CCDC25 levels after neoadjuvant treatment (Figure 6G).

We next investigated the role of CCDC25 in NET-mediated chemoresistance in vitro. In line with the clinical data, NET treatment in vitro impaired the cytotoxic efficacy of anthracycline drugs, which was efficiently reduced by *CCDC25* knockout in HR^+^ breast cancer cells, including MCF-7 cells and T47D cells (Figure 6H and Supplemental Figure 10, E–H). In contrast, NETs did not compromise the efficacy of chemotherapy in mesenchymal HR^–^ breast cancer cells like MDA-MB-231 cells (Supplemental Figure 10I). Besides, pretreatment with neutralizing antibody against CCDC25, but not isotype control, significantly attenuated the NET-induced chemoresistance in HR^+^ breast cancer cells (Figure 6, I and J). In vivo, knocking out *CCDC25* in MCF-7 cells that were inoculated into NOD/SCID mice significantly improved the efficacy of chemotherapy, demonstrated by the reduced tumor growth (Figure 6, K and L) and increased proportion of CK^+^TUNEL^+^ tumor cells (Figure 6M). To exclude the possibility that better response was due to less NET generation, we analyzed NET level in 4 groups and found that NET expression was similar in chemotherapy groups with or without *CCDC25* knockout (Supplemental Figure 10J). Similarly, targeting *Ccdc25* in 4T1 tumor cells also inhibited primary tumor growth, increased the number of CK^+^TUNEL^+^ tumor cells, and reduced lung metastasis (Figure 6, N and O, and Supplemental Figure 10, K–M). Moreover, in 4T1 tumors treated by radiotherapy, *Ccdc25* knockdown promoted tumor regression and significantly increased the number of TUNEL^+^ tumor cells (Supplemental Figure 10, N and O). To further clarify whether NET-DNA-CCDC25 axis also leads to chemoresistance in HER2-enriched breast cancer, we applied an anti-CCDC25 neutralizing mAb combined with chemotherapy in the spontaneous MMTV-PyMT FVB/NJ mouse model, which is a widely used genetically engineered mouse model to investigate EMT-related mechanisms (56). Consistently, the combination of anti-CCDC25 neutralizing mAb and chemotherapy significantly reduced tumor growth (Figure 6P) and lung and liver metastasis (Figure 6, Q and R), while NET levels were comparable in the presence or absence of anti-CCDC25 neutralizing antibody (Supplemental Figure 10, P and Q). Collectively, we demonstrate that NET-CCDC25 interaction triggers chemotherapy resistance in breast cancer.

### NET-CCDC25 interaction promotes tumor cell chemoresistance via STAT3 activation and EMT.

**Figure 7:**
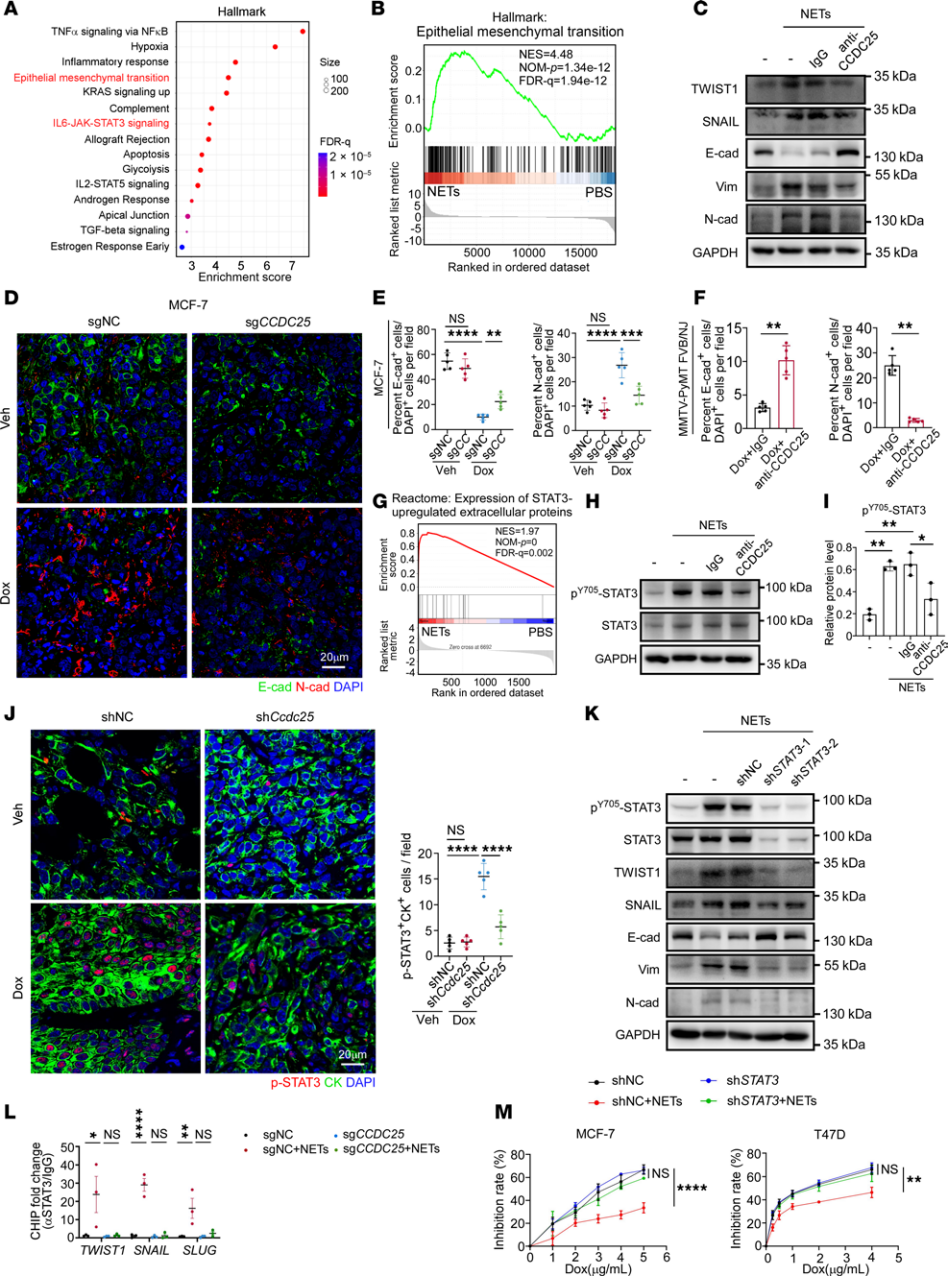
NET-CCDC25 interaction promotes tumor cell chemoresistance via STAT3 activation and EMT. (**A** and **B**) Enrichment analysis showing top 15 upregulated Hallmark pathways (**A**) and GSEA of the enriched epithelial mesenchymal transition pathway (**B**) in NET-treated MCF-7 cells from RNA-Seq. *P* values for GSEA were determined by permutation test. (**C**) Representative immunoblot of TWIST1, SNAIL, E-cadherin (E-cad), Vimentin (Vim), and N-cadherin (N-cad) in MCF-7 cells with indicated treatments. (**D** and **E**) Representative images staining for E-cad and N-cad (**D**) and quantification (**E**) in MCF-7 tumors with indicated treatments (*n* = 5/group, 2-way ANOVA with Šídák’s test). Scale bar, 20 μm. (**F**) Quantification of the percentage of E-cad^+^ and N-cad^+^ cells in tumors from MMTV-PyMT FVB/NJ mice (*n* = 5/group, Mann-Whitney test). (**G**) GSEA showing the enriched Reactome pathway in NET-treated MCF-7 cells (permutation test). (**H** and **I**) Representative immunoblot of p^Y705^-STAT3 and STAT3 in indicated groups (**H**) and quantification of relative protein levels using ImageJ software (*n* = 3/group, 1-way ANOVA with Tukey’s test) (**I**). (**J**) Representative images staining for p^Y705^-STAT3 and CK and quantification of p^Y705^-STAT3^+^ CK^+^ cells in 4T1 tumors from indicated groups (*n* = 5/group, 2-way ANOVA with Tukey’s test). Scale bar, 20 μm. (**K**) Representative immunoblot of p^Y705^-STAT3, STAT3, TWIST1, SNAIL, E-cad, Vim, and N-cad in MCF-7 cells with indicated treatments. (**L**) Localizations of STAT3 to the promoters of *TWIST1*, *SNAIL*, and *SLUG* genes in MCF-7 cells from indicated groups were analyzed by ChIP assay using anti-STAT3 antibody or control IgG (*n* = 3/group, 2-way ANOVA with Šídák’s test). (**M**) The growth inhibition rates of Dox on MCF-7 cells and T47D cells in indicated groups (*n* = 3/group). Data were compared at 5 or 4 μg/mL Dox, respectively, and analyzed by 2-way ANOVA with Šídák’s test. Data represent mean ± SD. Data represent 3 independent experiments in (**C** and **K**). ****P < 0.0001, ***P < 0.001, **P < 0.01, *P < 0.05.

To investigate the mechanism of NET-induced chemoresistance, we performed transcriptome analysis via RNA-Seq on MCF-7 cells stimulated with or without NETs in vitro. Principal component analysis segregated NET-treated cells from PBS-treated cells, indicating that NETs induced obvious transcriptional variance (Supplemental Figure 11A). Next, we employed enrichment analysis of the Hallmark gene set depending on DEGs in 2 groups. Among the top 15 enriched terms observed after NET treatment, we identified the presence of epithelial-mesenchymal transition pathway (Figure 7A), which was further confirmed by GSEA (Figure 7B).

EMT program represents a potential mechanism through which malignant cells acquire aggressive traits, such as invasion, migration, and therapeutic resistance (57, 58). It was recently shown that NETs could induce EMT in breast cancer cells and promote distant metastasis (59), but the mechanism remained obscure. Therefore, we evaluated whether the NET-induced EMT was associated with CCDC25. As anticipated, NET treatment led to decrease of epithelial marker E-cadherin; increase of mesenchymal markers Vimentin and N-cadherin and of EMT-related transcriptional factors (EMT-TFs) TWIST1, SNAIL, and SLUG (57); and the conversion from a rounded shape to an elongated one, as evidenced by Western blotting (Figure 7C), immunofluorescence (Supplemental Figure 11B), and qRT-PCR (Supplemental Figure 11C). By comparison, pretreatment with neutralizing antibody against CCDC25 or its knockout substantially reversed the mesenchymal state elicited by NETs (Figure 7C and Supplemental Figure 11, B and C). Additionally, in agreement with findings in vitro, chemotherapy- or radiotherapy-treated breast tumor also upregulated the EMT markers, while silencing of *CCDC25* in tumor cells or application of anti-CCDC25 neutralizing mAb decreased this effect (Figure 7, D–F, and Supplemental Figure 11, D–G), suggesting that NET-CCDC25 interaction played an indispensable role in EMT. Previous investigations have reported that the deficiency of HR in triple-negative breast cancer drives the shift toward the mesenchymal phenotype (60, 61). Thus, this may explain the limited induction of chemoresistance in mesenchymal MDA-MB-231 cells treated by NETs that have undergone EMT. These findings further support our observation that NETs and CCDC25 have greater prognostic value in patients with HR^+^ rather than HR^–^ breast cancer.

To unravel the mechanism of CCDC25 in EMT promotion, we focused on STAT3-related pathway and TGF-β signaling, which were significantly enriched under NET stimulation in RNA-Seq of MCF-7 cells (Figure 7, A and G, and Supplemental Figure 11H). To validate this finding and clarify which pathway is mainly responsible for CCDC25-mediated changes, we treated MCF-7 cells with NETs in the presence or absence of CCDC25 blockade and found that NETs induced higher level of STAT3 phosphorylation and nuclear translocation of STAT3 via CCDC25 (Figure 7, H and I, and Supplemental Figure 11I), which was also confirmed by the 4T1 and MMTV-PyMT FVB/NJ mouse model with chemotherapy (Figure 7J and Supplemental Figure 11J). In contrast, though NET treatment slightly promoted activation of TGF-β signaling, shown by the elevated phosphorylation of SMAD3, this could not be reversed by CCDC25 blockade (Supplemental Figure 11K), suggesting that TGF-β activation was independent of CCDC25-mediated EMT.

Thus, we asked whether NET/CCDC25/STAT3 pathway could induce EMT and chemoresistance. Indeed, Western blot and immunofluorescence assay confirmed that knocking down *STAT3* or applying STAT3 phosphorylation inhibitor Stattic markedly decreased NET-induced STAT3 phosphorylation and interrupted the conversion of the EMT process (Figure 7K and Supplemental Figure 11, L and M). Furthermore, we performed ChIP assay with an anti-STAT3 antibody to detect whether NET treatment could promote enhanced binding of STAT3 to the promoter region of EMT-TFs. In agreement, enhanced binding of STAT3 toward EMT-TF promoters was observed was observed after NET treatment, which was decreased by *CCDC25* knockout (Figure 7L). Furthermore, we demonstrated that silencing *STAT3* largely reversed NET-induced chemoresistance (Figure 7M). Therefore, these data suggest that NET-CCDC25 interaction enhances phosphorylation of STAT3, thus promoting EMT and chemoresistance.

To further determine the downstream effector protein that binds to CCDC25 and induces STAT3 phosphorylation, we performed a pull-down assay using the lysate of MCF-7 cells transfected with His-tagged *CCDC25*. Based on the results of silver staining (Supplemental Figure 12A), we isolated the proteins that showed increased expression and fell within a molecular weight range of 40–70 kDa for subsequent mass spectrometry (MS) analysis. We further identified PKM2, which exhibited increased interaction with CCDC25 in the cells stimulated with NETs (Supplemental Figure 12, B and C). PKM2, a well-known rate-limiting enzyme that catalyzes the final step of glycolysis, also functions as an active protein kinase capable of phosphorylating STAT3 at Tyr705 (hereafter abbreviated as Y705), thereby activating gene transcription within the nucleus (62, 63). The binding of PKM2 with CCDC25 was further confirmed by an immunoprecipitation assay (Supplemental Figure 12D). Next, we transfected MCF-7 cells with *PKM2* shRNAs to verify whether PKM2 plays an indispensable role in NET-DNA/CCDC25–induced STAT3 phosphorylation. As expected, targeting *PKM2* largely inhibited NET-mediated STAT3 phosphorylation (Supplemental Figure 12E). As reported previously, PKM2 nuclear translocation activates its protein kinase function while decreasing pyruvate kinase activity (62). Thus, we applied Shikonin, a small molecular inhibitor known to block PKM2 nuclear translocation (64). Consistently, Shikonin markedly suppressed NET-induced STAT3 phosphorylation at Y705 (Supplemental Figure 12F). In accordance, NET-induced PKM2 nuclear translocation in MCF-7 cells was also inhibited by both high and low levels of Shikonin (Supplemental Figure 12G). Furthermore, to verify whether PKM2 acts downstream of NET-induced EMT in cancer cells, we stimulated control or *PKM2*-knockdown MCF-7 cells with or without NETs and detected EMT via immunofluorescence staining. In accordance, NET-mediated EMT was largely decreased after *PKM2* knockdown using shRNA (Supplemental Figure 12H), further demonstrating that PKM2 is a downstream effector of the NET-DNA/CCDC25 pathway, which induces STAT3 phosphorylation and EMT.

## Discussion

Over the past decades, neutrophil infiltration and NET generation have been prominently linked to tumor progression and poor prognosis in human cancers, as they modulate tumor cells (65) or the surrounding niche (66). Although primary breast tumor shows relatively low NET level (16, 38), we demonstrate that chemotherapy or radiotherapy substantially increases NET formation in primary tumor and metastatic organs. Elevated NETs not only reduce therapeutic efficacy but also predict poor prognosis in breast cancer. This finding is consistent with previous research showing that radiation can induce NET formation in bladder and breast cancer, reducing tumor-inhibitory effect (25, 67). More importantly, we identify NET-DNA/CCDC25 interaction as a key driver of therapeutic resistance, suggesting an observable and targetable pathway to improve therapy sensitivity in breast cancer.

Nucleic acids play a dual role in cancer, activating tumor immune surveillance while driving tumor progression and metastasis (68, 69). Therefore, it is crucial to restrain their pro-tumor effects while preserving beneficial antitumor immunity. During chemotherapy and radiotherapy, dying cells release cell-free nucleic acids, particularly DNA, causing secondary inflammation and toxicities (70). To mitigate this, DNase I and similar agents have been combined with chemotherapy to reduce systemic toxic side effects and improve survival. However, although DNase I has been widely utilized with great efficacy and safety, it also dampens cyclic GMP-AMP synthase/stimulator of interferon genes (STING) signaling and weakens antitumor immunity (71, 72). Here we identify the membrane NET-DNA sensor CCDC25 as a pro-tumor mediator and promising therapeutic target because of its increased accessibility and maximum maintenance of immune activation. Fortunately, strategies targeting CCDC25 are emerging and becoming more realistic for future use (11, 73, 74). Additionally, to avoid nonspecific DNA cleavage by DNase I and PAD4’s roles outside of NETosis (75, 76), we further inhibited NET formation through blockade of CXCR2, CFB, or macrophage phagocytosis, as well as *Cxcr2* or *Cfb* knockout. These strategies substantially reduced primary tumor growth and metastasis, indicating the critical role of NET-DNA in therapeutic resistance.

Luminal breast cancer (HR^+^), the most prevalent subtype (about two-thirds) (77), derives limited benefit from NAC (78), showing substantially lower pCR rates than HER2-enriched and triple-negative breast cancers (79). Therefore, it remains key to stratify patients with different therapeutic responses and improve chemotherapeutic outcome. However, recent studies exploring the mechanism of luminal breast cancer chemoresistance focus mostly on inherent variation of cancer cells (80, 81). Here we demonstrate that the NET-DNA/CCDC25 axis promotes chemoresistance of luminal breast cancer via inducing EMT in vitro and in vivo, linking TME–cancer cell interactions to chemoresistance. In accordance, higher NET-DNA and CCDC25 levels correlate with worse prognosis in patients with HR^+^ breast cancer. In contrast, as HR deficiency has been reported to induce mesenchymal transformation in breast cancer, NETs show limited influence on chemosensitivity of HR^–^ cells like MDA-MB-231 (82). Interestingly, cathepsin G in NETs was reported to kill *PIK3CA*-mutant colorectal cancer cells after being internalized via cell surface protein RAGE (83). Therefore, the discrepancy in intrinsic biological traits of cancer cells may explain why NETs play different or even contradictory roles in different cancer subtypes. Together, our study underscores the translational value of CCDC25-targeted therapy for alleviating chemoresistance for most luminal breast cancer.

In the TME, macrophages and neutrophils have been reported to interact extensively and promote tumor progression (84). We demonstrate that after chemotherapy and radiotherapy, macrophages could promote neutrophil infiltration, NETosis, and breast cancer progression. Recently, macrophage-mediated phagocytosis or efferocytosis triggered by therapies has been closely linked to augmented immunosuppression and impaired antitumor responses (85, 86). Consequently, small molecular inhibitors have been developed to block tumor debris removal, such as the MerTK inhibitor UNC2250 (36). Consistently, our study shows that inhibiting macrophage engulfment effectively decreases tumoral NETs and improves chemotherapy response.

However, directly blocking macrophage clearance of dead tumor cells may cause excessive inflammation and secondary necrosis, ultimately driving tumor progression (87). Thus, targeting downstream effectors of macrophage engulfment, including immuno-inhibitory molecules and NET-inducing factors, is more rational. Our study shows that inhibiting CXCR2 or CFB in combination with chemotherapy or radiotherapy reduces neutrophil mobilization and NET generation. Notably, several CXCR2 inhibitors and mAbs have shown therapeutic potential, such as SB225002 (88), AZD5069 (89), and mAbs (90). Similarly, the CFB antagonist iptacopan (LNP023) has demonstrated efficacy and good tolerability in an ongoing phase III trial for IgA nephropathy, suggesting promising application in breast cancer (30). However, proposed strategies to prevent neutrophil accumulation or NET formation or degrade existing NETs may compromise host defense against invading pathogens mediated by antimicrobial proteins in NETs, like LL-37, elastase, and MPO (91, 92). Therefore, directly targeting CCDC25 represents a more translatable choice to improve treatment efficacy while preserving protective NET guarding functions.

Overactivation of STAT3 signaling has been associated with breast cancer progression, metastasis, and therapeutic resistance (93). While constitutive STAT3 activation is common in advanced breast cancer, the underlying mechanisms vary with the complex microenvironment. Our study reveals that NET-DNA promotes STAT3 phosphorylation via CCDC25, thereby facilitating cancer cell EMT and chemoresistance, consistent with the fact that cancer cell EMT reduces chemotherapeutic sensitivity (94).

In summary, chemotherapy- and radiotherapy-induced NETs, driven by macrophage-derived CXCL1/2 and CFB, promote therapeutic resistance. Elevated NETs hinder treatment efficacy by binding cancer cell CCDC25, leading to STAT3 activation and inducing EMT. Our study offers preclinical evidence supporting that combining CCDC25 inhibition with chemotherapy or radiotherapy may improve breast cancer outcomes.

## Methods

### Sex as a biological variable.

All mouse and patient samples used in this study were from females because breast cancer is primarily relevant in females.

### Statistics.

Data are shown as mean ± SD or mean ± SEM from at least 3 independent biological experiments. All the statistical analysis in this study was conducted using R, GraphPad Prism 9, and ImageJ (NIH). Statistical tests included 2-tailed paired or unpaired Student’s *t* test, Welch’s *t* test, Mann-Whitney *U* test, or Wilcoxon’s signed-rank test for comparisons between 2 groups. One-way ANOVA with Tukey’s test, Welch’s ANOVA with Dunnett’s T3 test, or Kruskal-Wallis test with Dunn’s test were used for comparisons among multiple groups. For dose-dependent assays, growth curves and experiments between 2 genotypes, statistical analysis was conducted using 2-way ANOVA or 2-way repeated measures ANOVA with Tukey’s or Šídák’s test. Kaplan-Meier curves with log-rank test and Cox’s regression analyses were used for survival analysis. The χ^2^ test or Fisher’s exact test was used to analyze cell proportions and patients’ response rates. Permutation test was used to calculate *P* values in GSEA. *P* < 0.05 was considered statistically significant.

### Study approval.

All animal experiments were approved by the Institutional Animal Care and Use Committee at the Medical School of Sun Yat-Sen University. Only female mice were used to avoid the sex bias. Clinical samples were collected with written informed consent, and all related procedures were performed with the approval of the internal review and ethics boards of the Sun Yat-Sen Memorial Hospital.

### Data availability.

Values for all data points in graphs are reported in the Supporting Data Values file. The raw sequencing data of mice have been deposited in the Genome Sequence Archive (GSA) database under accession code CRA028580, while human data were deposited in GSA-Human under accession code HRA012635. The raw data of MS have been deposited in integrated proteome resources (iProX) database under accession code PXD067184. Additional methods are available in Supplemental Methods.

## Author contributions

HL, Yetong Zhang, J Lin, and JZ share a specific co–first author position. The order of authorship was determined based on authors’ overall contributions to the project. LY, MN, ES, and HL designed and directed the study. HL, Yetong Zhang, J Lin, and JZ performed the cell and animal studies. HL, Yetong Zhang, JZ, X Liang, and SW analyzed the data and prepared the figures. X Liu, X Liang, ZL, XZ, Yunyi Zhang, XY, J Li, and XY performed Western blot and immunofluorescence staining experiments. HL, JZ, X Liang, and SW wrote the manuscript. LX, LY, MN, and Yetong Zhang provided feedback on the manuscript. All authors discussed the results and commented on the manuscript.

## Funding support

- Natural Science Foundation of China (82488101, 82222055, 32270971, 82573214, 82330056, 92159303, and 82002786).
- Noncommunicable Chronic Diseases-National Science and Technology Major Project (2024ZD0519800).
- China Postdoctoral Science Foundation (2024M763778).
- Youth S&T Talent Support Programme of Guangdong Provincial Association for Science and Technology (SKXRC2025132).
- Natural Science Foundation of Guangdong Province (2024A1515011150, 2021A1515010230).
- Guangzhou Basic and Applied Basic Research Scheme (2023A04J1765).
- National Key Research and Development Program of China (2021YFA1300602).
- Science and Technology Planning Project of Guangdong Province (2023B1212060013, 2023B110005).
- Science and Technology Program of Guangzhou (2024B01J1154).
- Bureau of Science and Technology of Guangzhou (20212200003).
- Program for Guangdong Introducing Innovative and Entrepreneurial Teams (2019BT02Y198).
- High-tech, Major and Characteristic Technology Projects in Guangzhou Area (2023-2025) (2023P-ZD14).

## Supplementary Material

Supplemental data

Unedited blot and gel images

Supporting data values

## Version 1 01/02/2026

Electronic publication
