# Supplementary material for: Tumor-derived neutrophil extracellular trap–associated DNA impairs treatment efficacy in breast cancer via CCDC25-dependent epithelial-mesenchymal transition
Source: J Clin Invest. 2026 Jan 2;136(1):e190557. doi: 10.1172/JCI190557 (PMC12721893; doi:10.1172/JCI190557)
Supplement: Supplemental data [file jci-136-190557-s038.pdf]

Supplemental Materials For

Tumor-derived neutrophil extracellular trap-associated DNA impairs treatment efficacy in breast cancer via CCDC25-dependent epithelial-mesenchymal transition

Heliang Li[1,2,*], Yetong Zhang[1,*] , Jianghua Lin[1,*], Jiayi Zeng[1,*], Xinyan Liang[1], Linxi Xu[1], Jiang Li[1], Xiaoming Zhong[1], Xu Liu[1], Zhou Liu[1,3], Xinyu Yang[1], Yunyi Zhang[1], Shun Wang[1], Erwei Song[1,4,5,†], Man Nie[6,†], Linbin Yang[1,4,†].

[1]Breast Tumor Center, Guangdong Provincial Key Laboratory of Malignant Tumor Epigenetics and Gene Regulation, Medical Research Center, Sun Yat-sen Memorial Hospital, Sun Yat-sen University, Guangzhou, Guangdong, China.

[2]Department of Thyroid Surgery, Sun Yat-Sen Memorial Hospital, Sun Yat-Sen University, Guangzhou, Guangdong, China.

[3]Department of Breast and Thyroid Surgery, Renmin Hospital of Wuhan University, Wuhan, Hubei, China.

[4]Zenith Institute of Medical Sciences, Guangzhou, Guangdong, China.

[5]State Key Laboratory of Proteomics, Beijing 102206, China.

[6]Department of Medical Oncology, State Key Laboratory of Oncology in South China, Guangdong Provincial Clinical Research Center for Cancer, Collaborative Innovation Center for Cancer Medicine, Sun Yat-sen University Cancer Center, Guangzhou, Guangdong, China.

[*]The authors contributed equally to this work.

[†]Shared corresponding authors.

The PDF file includes:

Supplemental Methods

Supplemental Figures 1-12

Supplemental Tables 1-3

Supplemental Reference

Supplemental Methods

Tumor cell lines

Human breast cancer cell lines MDA-MB-231, MCF-7 and T47D, mouse breast cancer cell lines 4T1 and PY8119 were obtained from American Type Culture Collection. Mouse breast cancer cell line EO771 was obtained from CH3 Biosystems. All the cells were tested negative for mycoplasma and cultured in DMEM containing 10% fetal bovine serum (FBS) according to standard protocols.

Patients and tissue samples

Paraffin-embedded breast cancer samples for CCDC25 and NET quantification were obtained from 466 female patients who were all diagnosed with invasive breast carcinoma (stage I-III) between 2008 and 2019 and underwent neoadjuvant chemotherapy at the Sun Yat-Sen Memorial Hospital, Sun Yat-Sen University (Guangzhou, China). Among them, pre-NAC biopsy samples were available for 81 patients. Paired biopsy samples (pre-NAC) and surgically resected samples (post-NAC) were collected from the same patients. Blood samples were obtained from 81 patients treated with or without NAC.

Therapeutic efficacy was assessed according to the standard of Response Evaluation Criteria in Solid Tumors (RECIST). Complete Response (CR) refers to disappearance of all lesions in both primary tumor and lymph nodes; Partial Response (PR) refers to at least a 30% reduction in the sum of the longest diameter of target lesions; Progressive

Disease (PD) refers to at least a 20% increase in the sum of the longest diameter of target lesions; and Stable Disease (SD) refers to neither sufficient shrinkage to qualify as PR nor sufficient increase to qualify as PD. Patients with CR or PR were classified as sensitive, and those with SD or PD were classified as resistant to neoadjuvant therapy.

Animal experiments

All of *Pad4*$^{-/-}$, *Cxcr2*$^{-/-}$ and *Cfb*$^{-/-}$ mice (C57BL/6J background) were generated by Shanghai Model Organisms Center using a CRISPR-Cas 9-mediated genome editing system as previously described (1, 2). Other wildtype C57BL/6J mice, BALB/c mice and NOD/SCID mice were purchased from GemPharmatech. All mice were maintained and experiments were conducted in specific pathogen free (SPF) animal house under a 12-h light/12-h dark cycle, with ambient temperature controlled between 20 and 26 °C and relative humidity maintained at 40-60% according to a protocol approved by the Institutional Animal Care and Use Committee at the Medical School of Sun Yat-Sen University and laboratory animal facility has been accredited by AAALAC (Association for Assessment and Accreditation of Laboratory Animal Care International) and the IACUC (Institutional Animal Care and Use Committee) of Guangdong Laboratory Animal.

For orthotopic tumor models, 4T1 (2×10^5), PY8119 (2×10^5), EO771 (2×10^6) and MMTV-PyMT (C57BL/6J background) (5×10^5) cells were implanted into the fat pads of 6-8-week-old female BALB/c or C57BL/6J mice. After the xenografts became

palpable (around 100-200 mm^3), the mice were administered with 5 mg/kg Dox (Cat# HY-15142A, MCE) or equal volume of sterile saline solution every 4-7 days via intraperitoneal (i.p.) injection. Volume of tumors was measured every 3-5 days using caliper and calculated using the formula: volume = length × $width^2/2$. To remove macrophages in 4T1 tumors pharmacologically, mice were either treated with 200 μL/20 g PBS- or Clodronate-containing liposomes (Cat# CP-005-005, Liposoma) i.p. weekly, or 400 μg anti-CSF1R mAb (Cat# BE0213, Bio X Cell) i.p. twice weekly or equal volume of rat IgG2a isotype control (Cat# BE0089, Bio X Cell). For the blockade of CXCR2 or CFB, 10 mg/kg SB225002 (Cat# HY-16711, MCE) was injected i.p. or 30 mg/kg LNP023 (Cat# HY-127105, MCE) was administrated intragastrically (i.g.) every day since chemotherapeutic treatment started. For the degradation of NETs in mouse tumors after chemotherapy and radiotherapy, we injected 5 mg/kg DNase I (Cat# D806930, Macklin) i.p. daily. For the inhibition of phagocytosis in vivo, inhibitor of MerTK, UNC2250 (Cat# HY-15797, MCE), was used at the dosage of 10 mg/kg daily via oral gavage (3) since one day before Dox treatment in 4T1 tumor-bearing mice. For focal tumor radiotherapy, 8 Gy was given to 4T1 tumor- or EO771 tumor-bearing mice every time using X Ray Irradiator (Rad Source Technologies, Inc, RS2000 Pro) as previously described (4). For co-injection experiments, 4T1 (1×10^5) cells alone or mixed with equivalent amount of 4T1 cell debris suspended in 100 μL PBS were injected into the mammary fat-pads as previously described (5).

Three days prior to the inoculation of MCF-7 cells in NOD/SCID mice, a 1.7 mg 17β-

estradiol 60-day release pellet (Cat# SE-121, Innovative Research of America, Sarasota, FL, USA) was subcutaneously implanted in order to support the estrogen-dependent tumor growth (6). Then 5×10^6 MCF-7 cells were injected into the fat-pads of female six-week-old NOD/SCID mice and 2 mg/kg Dox was administrated i.p. when tumor volume reached 150-200 mm^3. For focal tumor radiotherapy in MCF-7 tumor-bearing mice, one dose of 5 Gy was given using X Ray Irradiator (Rad Source Technologies, Inc, RS2000 Pro). For the degradation of NETs in MCF-7 tumor after chemotherapy and radiotherapy, we injected 5 mg/kg DNase I i.p. every two days. For the blockade of CXCR2 or CFB in MCF-7 tumors, 10 mg/kg SB225002 was injected i.p. or 30 mg/kg LNP023 was administrated i.g. twice weekly since treatment started.

The spontaneous, transgenic MMTV-PyMT breast cancer models (on FVB/NJGpt or C57BL/6J background) were purchased from GemPharmatech. Only female mice with the same date of birth were used. For MMTV-PyMT FVB/NJ mouse model, when the first tumor became palpable, mice were randomly assigned to different treatment groups and tumor volume was measured. Mice were sacrificed at the endpoint (week 12 or 13 after birth, or when the biggest tumor reached 20 mm on longest diameter or ulcerated, or the mice showed any sign of distress or weight loss). For the digestion of NETs, 5 mg/kg DNase I (Cat# D806930, Macklin) was injected i.p. daily when tumor volume reached 100-150 mm^3. For the inhibition of CCDC25, 5 mg/kg monoclonal, neutralizing anti-CCDC25 antibody (Sobour, Guangzhou, Biopharmaceutical Co., Ltd.) or equal volume of mouse IgG1 isotype control (Cat# BE0083, Bio X Cell) was

administrated i.p. twice weekly. For chemotherapy, 5 mg/kg Dox was applied i.p. when tumor volume reached 300-400 mm^3 on week 7, 8 and 9.

For experimental liver metastasis mouse model, 2×10^5 EO771 breast cancer cells were suspended in 50 μL PBS and injected intrasplenically as previously described (1). Then 5 mg/kg Dox was injected i.p. every 10 days.

For experimental lung metastasis model, 1×10^5 4T1 cells in 100 μL PBS were injected via tail vein and 5 mg/kg Dox with or without treatments of vehicle, SB225002 or LNP023 were applied as demonstrated above.

Mice were sacrificed at the endpoint or when tumor volume reached 1.5 cm^3. Then tumor volume or weight was compared among groups and tumors (and metastatic livers and lungs) were collected for further analysis and experiments.

Primary human peripheral blood immune cell isolation

Primary peripheral blood mononuclear cells were obtained as previously described (7). Briefly, primary peripheral blood mononuclear cells were isolated from whole blood by Ficoll-Paque PLUS (Cat# LTS1077, TBD science) through density gradient centrifugation (37°C, 450 × g, 20 min). Afterwards, the mononuclear cell layer was carefully transferred to a clean 50 mL conical tube and washed thoroughly for four times with PBS to reduce platelet contamination. Macrophages were obtained by

culturing monocytes in DMEM supplemented with 10 ng/mL M-CSF (Cat# 300-25, PeproTech) and 10% FBS at 37°C with 5% CO2 for 7 days. And the purification of neutrophils and NETs was conducted as previously described (1). Briefly, human neutrophils were isolated using CD66b^{+} microbeads (Cat# 130-104-913, Miltenyi Biotec). After stimulating neutrophils with 500 nM PMA (Cat# HY-18739, MCE) for 4 hr, the culture medium was removed gently and NETs adhered at the bottom of plates were pipetted with 1 mL cold PBS, followed by centrifugation at 4°C (1000 × g, 10 min). The DNA concentration was then detected by spectrophotometry and NETs were saved at -80°C for further experiments.

Mouse bone-marrow-derived macrophages (BMDMs) isolation

The cavities of femurs and tibias were flushed with 1-mL sterile syringes to obtain bone marrow cell suspensions, which were filtered through 70-μm cell strainers and washed with PBS twice. To isolate mouse macrophages, harvested cells were cultured in DMEM supplemented with 10% FBS, 1% penicillin/streptomycin and 10 ng/mL mouse M-CSF (Cat# 315-02, PeproTech) at 37°C with 5% CO_2 for 7 days. For the blockade of enzyme activity of cathepsins, AZD7986 (Cat# HY-101056, MCE) and E-64 (Cat# HY-15282, MCE) were used. For the blockade of NF-κB pathway, inhibitors JSH-23 (Cat# HY-13982, MCE) and Sc-3060 (Cat# sc-3060, Santa Cruz) were used.

shRNA-mediated silencing or CRISPER-mediated gene knockout

For transduction of tumor cells, cells were grown to 30-40% confluence in 6-well plates

and treated with lentiviral particles (multiplicity of infection (MOI) of 20 for MCF-7 and T47D cells; MOI of 10 for MDA-MB-231, EO771 and 4T1 cells) and 8 μg/mL Polybrene (Cat# TR-1003-G, Sigma-Aldrich) at 37℃ with 5% CO_2 overnight. Next, medium with lentiviral particles was discarded and replaced with fresh complete DMEM. After 48-hr incubation, 2.5 μg/mL puromycin (Cat# A1113803, Gibco) was added into the complete DMEM medium to screen successfully transduced cells for consecutive 14 days. The knockdown or knockout efficiency was verified by qRT-PCR or western blot.

Lentiviral shRNAs targeting *Ccdc25*, *Ctsc*, *STAT3* and *PKM2* were obtained from GENECHEM Inc (Shanghai, China). The Cas9 lentivirus and gRNA lentivirus targeting *CCDC25* were obtained from Beijing Tsingke Biotech Co., Ltd. The targeting sequences of each shRNA and sgRNA are listed as follows:

Ccdc25 shRNA-1 5’-GAAGGCCAATAGCATTCAAGG-3’

Ccdc25 shRNA-2 5’-GCAGAGAAAGAATGCAGAGAT-3’

Ctsc shRNA 5’-CGACATTAACTGCTCGGTGAT-3’

STAT3 shRNA-1 5’-CAGCTGAACAACATGTCATTT-3’

STAT3 shRNA-2 5’-GTGGTACAACATGCTGACCAA-3’

PKM2 shRNA-1 5’-GCTGTGGCTCTAGACACTAAA-3’

PKM2 shRNA-2 5’-GTTCGGAGGTTTGATGAAATC-3’

NC shRNA 5’-TTCTCCGAACGTGTCACGT-3’

CCDC25 gRNA-1 5'-GTCTGGGAACCGCTCGACTT-3'

CCDC25 gRNA-2 5'-GAATGCTATTGGCCTTCACA-3'

NC gRNA 5'-GGTTCTCCGAACGTGTCACGT-3'

siRNA-mediated silencing

siRNAs targeting *Ctsc*, *Hmgb1*, *S100a9*, *Tctp*, *Hsp70*, *Tlr4*, *Myd88* and *Trif* were obtained from Beijing Tsingke Biotech Co., Ltd. The targeting sequences of each siRNA are listed in Supplemental Table 2.

Matured macrophages were seeded into 24-well plates at a confluency of approximately 60-70% with 900 μL fresh complete culture medium. siRNA powder was reconstituted in nuclease-free water at a concentration of 100 mM. Transfection solution was prepared by adding 100 μL serum-free Opti-MEM medium to a nuclease-free EP tube, followed by adding 20-50 nM of siRNA as required. The mixture was aspirated and expelled 10 times using a pipette tip, then 2 μL INTERFERin® transfection reagent (Cat# 101000028, Polyplus) was added, mixed by pipetting 10 times, and left undisturbed at room temperature (RT) for 10 minutes. The solution was then added to the cells in 24-well plates and cultured at 37°C with 5% CO_2 for 2-3 days before subsequent experiments. The knockdown efficiency of siRNA was verified by qRT-PCR.

Enzyme-linked immunosorbent assay (ELISA) and MPO-DNA detection

For the isolation of mouse serum, whole blood samples obtained from mouse were collected in clean 1.5-mL EP tubes and left undisturbed at RT for 1 hr to clot. After clotting, blood samples were centrifuged for 20 min (2000 × g, 4°C). For the isolation of human plasma, peripheral blood was collected with tubes containing 25 μL 0.5 M EDTA, followed by centrifugation for 10 min (2000 × g, 4°C). Then the top layer was carefully collected without disturbing the separated layers, and transferred into new tubes for storage at -80°C.

For the detection of CXCL1, CXCL2 and CFB, ELISA kits of CXCL1 (Cat# E-EL-M0018, Elabscience), CXCL2 (Cat# E-EL-M0019, Elabscience) and CFB (Cat# E-EL-M0334, Elabscience) were used, and the assay was carried out following the manufacturer's instruction. If the concentration exceeds the specified range, the samples will be diluted accordingly.

The quantification of MPO-DNA levels was conducted as previously described (1). Briefly, 96-well microtiter plates were coated with 5 μg/mL anti-MPO monoclonal antibody (Cat# 0400-0002, ABD Serotec) overnight at 4°C. After blocking with 1% BSA, samples along with peroxidase-labelled anti-DNA monoclonal antibody (component No.2 of the Cell Death Detection ELISA kit, Cat# 11774425001, Roche) were added and incubated for 2 hr at RT, followed by washing for three times with PBS. Next, the peroxidase substrate (Cat# 11774425001, Roche) was added for incubation at 37°C for 40 min. The optical density was measured at 405 nm using Infinite F500

(Tecan).

Chemotherapy-induced tumor cell debris generation and isolation

Dox was used to prepare tumor cell debris for in vitro and in vivo assays as previously described (5, 8). Briefly, 4T1 cells were cultured with complete medium until reaching 50-70% confluence in T-75 flasks, and then treated with 2 μg/mL Dox for 48 hr. For the staining of Deep Red CellTracker Dye (Cat# 34565, Thermo Scientific™), 4T1 cells were pre-dyed in sterile PBS at 37°C for 15 min. Dead cell debris in the culture medium was collected, counted by hemocytometer and centrifuged at 1500 rpm for 10 min. Afterwards, the pelleted debris was resuspended in sterile PBS and used for further experiments.

Immunohistochemistry (IHC) and immunofluorescence (IF)

Paraffin-embedded samples were sectioned at 4-μm thickness, deparaffinized and rehydrated. Heat-mediated antigen-retrieval was performed using Tris-EDTA buffer (pH 8.0 or 9.0, Asegene) for 4 min in a pressure cooker. For IHC, the sections were incubated with endogenous peroxidase blocking buffer (Cat# C5216, bioss) for 10 min at RT, and subsequently blocked with 5% BSA solution for 20 min at RT. The tissue sections were incubated with anti-CCDC25 antibody (1:100, Cat# 21209-1-AP, ProteinTech) at 4°C overnight. The next day, samples were washed in PBS for three times and stained with IHC secondary antibody (Cat# PV-6000, ZSGB-BIO) for 1 hr at RT following the manufacture's instruction. Images were obtained by KFBIO Digital

Pathological Slide Scanner KF-PRO-120-H1 (KFBIO). The expression levels of CCDC25 were scored semiquantitatively by combining the proportion of positively stained cells with their staining intensity determined by Image J software as previously described (9). Staining intensity was defined as: 0 = negative; 1 = weak; 2 = moderate; 3 = strong. And the IHC score was calculated using the following formula: [0 × (% cells negative) + 1 × (% cells weak) + 2 × (% cells moderate) + 3 × (% cells strong)]. We then used the X-tile software to determine the best cut-off point and defined CCDC25-high and CCDC25-low in our breast cancer cohort for the following analysis.

For tissue immunofluorescence, samples were treated as described above, and directly blocked with 5% BSA for 20 min at RT. After blockade, samples were incubated with primary antibodies specific for: MPO (1:50, Cat# AF3667, R&D), H3cit (1:100, Cat# ab281584, Abcam), Ly6G (1:100, Cat# ab238132, Abcam), F4/80 (1:100, Cat# Sc-52664, Santa Cruz), CD68 (1:100, Cat# Ab201340, Abcam), Luciferase (1:100, Cat# NB100-1677, Novus), CXCL1 (1:50, Cat# AF5403, Affinity), CXCL2 (1:50, Cat# P19875, Bioss), CFB (1:100, Cat# DF6567, Affinity), GFP (1:100, Cat# T0006, Affinity), PyMT (1:100, Cat# NB100-2749, Novus), CK (1:100, Cat# Ab7753, Abcam), N-cadherin (1:100, Cat# Ab207608, Abcam), Vimentin (1:100, Cat# ab92547, Abcam), E-cadherin (1:100, Cat# ab40772, Abcam), P^{Tyr705}-STAT3 (1:50, Cat# Ab76315, Abcam) overnight at 4°C. After washing in PBS for three times, the slides were incubated with fluorescence-conjugated secondary antibodies for 1 hr at RT, and DAPI solution (Cat# P005906, Asegene) was then applied for counterstaining. Images were obtained by laser

scanning confocal microscopy (LSM800, Zeiss) or KFBIO Digital Pathological Slide Scanner KF-PRO-120-H1 (KFBIO). NET levels were determined as the percentage of the positive signal co-stained for MPO and H3cit in each field of view using HALO software (Indica Labs). For the quantification of NET expression, the number of Ly6G^{+}, CXCL1^{+}, CXCL2^{+}, CFB^{+}, F4/80^{+} Luci^{+}/GFP^{+}/PyMT^{+}, p-STAT3^{+}/TUNEL^{+} CK^{+}, E-cad^{+}, N-cad^{+} and Vim^{+} cells in tissue, 5 representative IF images were randomly selected from each tissue section. The accuracy of automated measurements was confirmed by two independent investigators, who were unaware of the patients' clinical information or mouse group information.

For TUNEL assay, the staining of slides was carried out with One Step TUNEL Apoptosis Assay Kit (Cat# C1089, Beyotime) following the manufacture's instruction. Briefly, the samples were permeabilized with proteinase K solution for 15 min at 37°C, and then incubated with the TUNEL solution for 1 hr at RT, followed by couterstaining with DAPI. Images were obtained by 3DHistech Slide Scanner.

For IF staining of cells cultured in vitro, cells growing on the chamber slides (Cat# 154453, Thermo Scientific™ Nunc™ Lab-Tek™ II Chamber Slide™) were fixed with 4% paraformaldehyde for 15 min at RT, washed in PBS and permeabilized with 0.1% Triton X-100 in PBS for 5 min. 5% BSA was used to block the non-specific site for 15 min at RT. Afterwards, cells were incubated with primary antibodies against p65 (1:100, Cat# 8242, CST), TLR4 (1:100, Cat# AF7017, Affinity), RAB5A (1:100, Cat# 46449S,

CST), CK (1:100, Cat# Ab7753, Abcam), STAT3 (1:100, Cat# Ab68153, Abcam), N-cadherin (1:100, Cat# Ab207608, Abcam), Vimentin (1:100, Cat# ab92547, Abcam), E-cadherin (1:100, Cat# ab40772, Abcam), TWIST1 (1:100, Cat# Sc-81417, Santa Cruz), SNAIL (1:100, Cat#13099-1-AP, ProteinTech), PKM2 (1:2000, Cat# 4053, CST), overnight at 4°C, followed by incubation with Alexa Flour secondary antibodies for 1 hr at RT. Then cells were counterstained with DAPI and images were acquired with LSM 800 confocal microscope.

Flow cytometry

Mouse monocyte-derived macrophages were isolated and cultured as described above. After co-culturing with CellTracker^{TM} Deep red^{+} (Cat# C34565, Thermo Scientific™)4T1 cell debris for 2 hr, adherent macrophages were digested using 1× TrypLE (Cat# 12604021, Gibco) and collected (350 × g, 5 min) for flow cytometry. After washing in phosphate buffered saline (PBS) for two times, cells were resuspended in PBS containing 1% FBS, followed by blockade with CD16/32 antibody (Cat# 101319, BioLegend) for 15 min at 4 °C. Then cells were stained with FITC anti-mouse CD11b (Cat# 101205, BioLegend), Pacific blue anti-mouse F4/80 (Cat# 123124, BioLegend) and 1 μg/mL Fixable Viability Dye eFlour^{TM} 780 (Cat# 65-0865-14, Thermo Scientific™) for 30 min at 4°C.

For Apoptosis Assay, MCF-7 or T47D cells were seeded in 24-well plates and cultured with DMEM containing 10% FBS in the presence or absence of NETs (5 μg/mL) at 37°C

for at least 3 days. After treatment with Dox (4 μg/mL) for 24 hr, cells were trypsinized and harvested by centrifugation (350 × g, 5 min). Then, cells were incubated with 100 μL binding buffer (Cat# 422201, BioLegend) containing 5 μL APC-Annexin V antibody (Cat# 640920, BioLegend) and 1 μg/mL Fixable Viability Dye eFlour^{TM} 780 (Cat# 65-0865-14, Thermo Scientific™) or Fixable Viability Dye eFlour^{TM}520 (Cat# 65-0867-14, Thermo Scientific™) for 15 min at RT. Afterwards, cells were washed and resuspended in 100 μL binding buffer and examined immediately by Cytoflex S Flow Cytometer (Beckman Coulter). All data were analyzed using FlowJo V10 software (Tree star, Ashand, OR).

Quantitative reverse transcription PCR (qRT-PCR)

Total RNA from cells and tissues was extracted using TRIzol (Cat# 15596026CN, Thermo Scientific™) as described previously (10). Later, the extracted RNA was reversely transcribed into cDNA with PrimeScript RT reagent Kit (Cat# RR037B, Takara), and qRT-PCR was conducted with TB Green Advantage qPCR premixes (Cat# 639676, Takara). Data were collected and analyzed with a LightCycler 480 instrument (Roche). The primer sequences were listed in Supplemental Table 3.

Co-immunoprecipitation (co-IP) and mass spectrometry (MS)

A cDNA encoding full-length human *CCDC25* was subcloned into the pcDNA3.4 vector with a C-terminal 6X His tag. MCF-7 cells were then transfected with the indicated plasmid and treated with or without NETs. Whole-cell lysates from a sub-

confluent 10 cm dish of MCF-7 cells were prepared in IP lysis buffer with protease inhibitor cocktail (Cat# 78446, Thermo Scientific) and used for immunoprecipitation with antibodies against 6X His (Cat# Ab18184, Abcam) as previously described (1). Briefly, 5 μg anti-His antibody or matched IgG antibody (Cat# Ab18469, Abcam) was incubated with 1 mL cell lysate for 1 hr at RT, followed by an additional 50 μL of Protein A/G coupled magnetic beads (Cat# 88803, Thermo Scientific) overnight at 4°C to form and capture the immune complex. The protein complex bound on the beads was pelleted with a magnetic separation rack and washed with PBS buffer for five times. Immunoprecipitated proteins were then eluted in 1X loading buffer by boiling for 5 min, separated by SDS-PAGE and detected by western blotting using PKM2 (Cat# 4053, CST) and His (Cat# Ab18184, Abcam) antibodies. For liquid chromatography coupled with MS/MS (LC-MS/MS), proteins were separated on SDS-PAGE gels and visualized using Fast Silver Stain Kit (Cat# P0017S, Beyotime) following the manufacturer's protocol. Then targeted protein bands were excised and sent to BGI Tech Solutions Co., Ltd (Shenzhen, China) for analysis by LC-MS/MS.

Western Blot

Proteins were extracted from cells and tissues with RIPA buffer (Cat# P0013E, Beyotime) containing protease and phosphatase inhibitor cocktail (Cat# P1045, Beyotime), and then quantified using Pierce™ BCA Protein Assay Kits (Cat# 23225, Thermo Scientific™) according to standard protocols. After boiling in 1× loading buffer (Cat# AM8547, Thermo Scientific™), equivalent amount of protein was

resolved by SDS-PAGE gels and transferred to PVDF membranes. Primary antibodies specific for MPO (1:2000, Cat# AF3667, R&D), H3cit (1:1000, Cat# ab281584, Abcam), C3a (1:2000, Cat# HM1072, Hycult Biotech), C5a (1:1000, Cat# MAB21501, R&D system), C3b/iC3b/C3c (1:2000, Cat# HM1065, Hycult Biotech), CCDC25 (1:1000, Cat# 21209-1-AP, ProteinTech), STAT3 (1:3000, Cat# Ab68153, Abcam), p^{Tyr705}-stat3 (1:2000, Cat# Ab76315, Abcam), SMAD2/3 (1:1000, Cat# 8685, CST), Phosphor SMAD2(Ser465/467)/SMAD3(Ser423/425) (1:1000, Cat# 8828, CST), E-cadherin (1:1000, Cat# 3195, CST), N-cadherin (1:2000, Cat# Ab207608, Abcam), Vimentin (1:2000, Cat# Ab92547, Abcam), TWIST1 (1:2000, Cat# Sc-81417, Santa Cruz), SNAIL (1:1000, Cat# 3879, CST), CTSC (1:1000, Cat# sc-74590, Santa Cruz), PKM2 (1:2000, Cat# 4053, CST), 6X His (1:2000, Cat# Ab18184, Abcam), Vinculin (1:3000, Cat# 18799, CST), GAPDH (1:3000, Cat# HRP-60004, ProteinTech) and β-actin (1:3000, Cat# HRP-60008, ProteinTech) were diluted in TBST containing 5% BSA and incubated with membranes overnight at 4°C. The next day, peroxidase-conjugated secondary antibodies (1:3000, Cat# 7076, 7077, 7074, CST; Cat# SA00001-4, ProteinTech) were used, and the antigen-antibody reactions were visualized with enhanced chemiluminescence assays (ECL, Thermo Scientific™).

Cell Counting Kit-8 (CCK-8) assay

2×10^3 MCF-7 or T47D cells were seeded in the 96-well plates and incubated overnight at 37°C. After treatment with NETs (5 μg/mL) and/or dox (0.25, 0.5, 1, 2, 3, 4, 5 μg/mL), CCK8 kit (Cat# C0038, Beyotime) was added to individual wells according to the

manufacture's instruction, and the plates were incubated for 1 hr at 37°C. The absorbance was then measured at 450 nm by Infinite F500 (Tecan). Six replicate wells were included in each analysis and at least three independent experiments were carried out.

Chromatin immunoprecipitation (CHIP) assay

The CHIP assay was performed with a CHIP Assay kit (Millipore) based on the manufacturer's instructions. Briefly, 5 × 10^6 MCF-7 cells were fixed in 1% formaldehyde for 10 min at RT. Then cells were harvested, lysed and sonicated for 10 cycles of 10 s on/20 s off and 50% AMPL with Sonics VCX130 (Sonics & Materials, Inc, Newtown). Antibodies against STAT3 (Cat# 9139, CST) and rabbit IgG (Cat# A7016, Beyotime) (5 µL per 1mg total protein) were employed. The precipitated DNA was subjected to PCR amplification. Related primer sequences were listed as follows:

TWIST1-forw 5'-CCAGGTCGTTTTTGAATGGT-3'

TWIST1-rev 5'-ACGTGAGGAGGAGGGACTTT-3'

SNAIL-forw 5'-TCACTAAACGAGGCTGAGCA-3'

SNAIL-rev 5'-GCTCTTCCTCCTCTCCCCTA-3'

SLUG-forw 5'-CCTCCAGCACCTGTTAGAAAC-3'

SLUG-rev 5'-AAAGCATCTCTGTCCATTGC-3'

RNA sequencing (RNA-seq)

Tumor cells and macrophages with indicated treatments in vitro were homogenized directly in TRIzol reagent kit (Thermo Scientific™) using a TissueLyzer (Lu Ka). RNA quality was assessed on an Agilent 2100 Bioanalyzer (Agilent Technologies, Palo Alto) and checked using RNase free agarose gel electrophoresis. Then mRNAs were enriched by Oligo (dT) beads and fragmented and reversely transcribed into cDNA. The purified double-stranded cDNA fragments were end repaired, A base added and ligated to Illumina sequencing adapters. The ligation reaction was purified and PCR amplified. The resulting cDNA library was sequenced using Illumina Novaseq 6000 by Gene Denovo Biotechnology Co. (Guangzhou, China). Then 150 bp paired-end reads were generated and mapped to the reference genome using HISAT2 v2.1.4. The mapped reads were assembled using StringTie c1.3.1 and fragments per kilobase per million mapped reads (FPKM) were calculated to estimate gene expression in each sample. Genes with P-value ≤ 0.05 and $|\log2\_ratio| \geq 1$ are identified as DEGs. GSEA and bubble plots were generated by using R packages or GENI website.

ScRNA-seq and bioinformatics analysis

Sample Dissociation

Freshly isolated subcutaneous 4T1 tumors were dissected away from any surrounding fat, fibrous, necrotic areas and minced into small pieces in DMEM medium containing 10% FBS. Then tumor samples were minced into small pieces (1-2 mm in diameter) and digested in enzyme cocktail (including collagenase type I (Cat# LS004196, Worthington), collagenase type IV (Cat# LS004188, Worthington), DNase I (Cat#

D106200, Aladdin) for 30 min at 37°C under constant shaking to make a single cell suspension. Afterwards, cell suspensions were sequentially filtered through 100 and 70 μm cell strainers and washed twice with PBS. Filtered cells were incubated with red blood cell lysis buffer (Cat# R1010, Solarbio) to remove red blood cells.

Single-cell Library Preparation and Sequencing

Single cells were suspended in Dulbecco's PBS + 0.04% BSA and retained on ice. Cells were counted and assessed for viability with Trypan blue using a Countess II automated counter (Thermo Scientific™), and then resuspended at 1×10^5-2×10^5 cells/mL with a final viability of >90% as determined using the Countess. Single cells were encapsulated into emulsion droplets using Chromium Controller (10 × GENOMICS). scRNA-seq libraries were constructed using Chromium Single Cell 30 Reagent Kits v3 (10 × GENOMICS) according to the manufacturer's protocol. Cells were mixed with barcoded primer-linked gel beads. Each cell was uniquely barcoded with an index, and every transcript within one cell was uniquely barcoded with a unique molecular identifier (UMI). The cDNAs were pooled, truncated, and amplified to generate cDNA libraries that were sequenced by using an Illumina NovaSeq 6000 system.

scRNA-seq Data Processing and Downstream Analysis

FASTQ sequence data were processed to obtain a UMI count in each cell by using 10 × Genomics CellRanger (v3.0.2) pipeline. A mouse reference genome (mm10) was used for gene alignment. Subsequent single cell RNA-seq analysis was performed in R

package “Seurat” (v5.0.1). The gene-barcode matrix was passed through the R (v4.3.0) software package Seurat (v5.0.1) for downstream analysis. In the phase of quality control, cells with low quality (<200 or >6000 genes per cell and >10% mitochondrial genes in the cell) were filtered out. Data were normalized and scaled within each sample, and canonical correlation analysis was performed to integrate data across samples. Cell population deconvolution was performed by the dimensional reduction and shared nearest neighbor (SNN) modularity clustering algorithm. We defined the cell clusters based on a set of highly expressed differential/conservative/cell marker genes. DEGs in each cluster across different conditions were identified by using the “FindMarkers” function in Seurat. Within each cluster, DEGs between two groups of cells were identified by using a Wilcoxon rank sum test. Adjusted p-values were calculated on the basis of the Bonferroni correction. Genes with a p_val_adj < 0.05 were considered as DEGs. KEGG and GO analysis was performed by using an R package “ClusterProfiler”. GSEA analysis was performed by using an R package “GseaVis”.

Online dataset analysis

Kaplan-Meier analysis of the breast cancer and colon cancer samples receiving chemotherapy were performed by the KM Plotter website. Search entries: *CTSC* as the gene symbol (Affymetrix ID: 231234_at), *CCDC25* (Affymetrix ID: 218125_s_at), the auto-select best cut-off for “split patients by”. Both the survival plots and statistical analysis were generated by the website.

For database analysis, we downloaded high-through transcriptional data of patient breast cancer tissues from the Gene Expression Omnibus (GEO) database and single-cell analysis of mouse lung cancer tissues from the Genome Sequence Archive (GSA) database. GSEA analysis was visualized using the R package ClusterProfiler and Enrichment plot.

Supplemental Figures

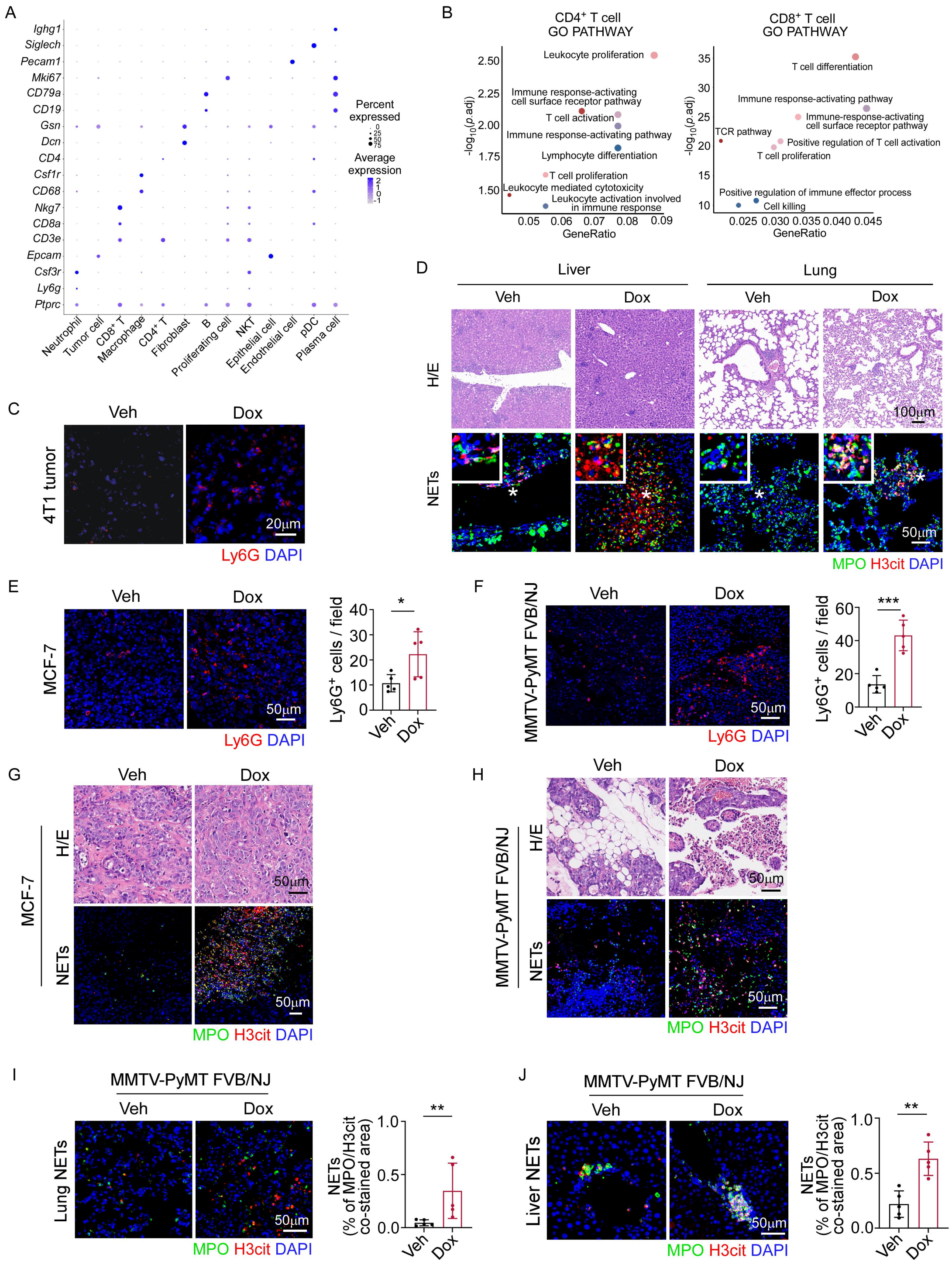

Supplemental Figure 1. Chemotherapy induces neutrophil infiltration and NETosis in breast cancer.

(**A**) Dot plot showing average expression and frequency of selected marker genes to define indicated clusters.

(**B**) GO enrichment analysis showing the upregulated pathways in $CD4^{+}$ and $CD8^{+}$ T cell subsets from the Dox-treated group in comparison with Veh-treated group (hypergeometric test).

(**C**) Representative IF images of tumoral Ly6G$^+$ cells in 4T1 tumors from indicated groups (n=8/group). Scale bar, 20 μm.
(**D**) Representative H&E images and immunofluorescent images staining for MPO and H3cit in livers and lungs from 4T1 tumor models (n=8/group). Scale bars, 50 μm and 100 μm. * indicates the areas that are magnified in the insets in the top-left corners.
(**E**) Representative IF images and quantification of Ly6G$^+$ neutrophil numbers per field in MCF-7 tumors from indicated groups (n=5/group). Scale bar, 50 μm. Data were analyzed by unpaired *t*-test.
(**F**) Representative IF images and quantification of tumoral Ly6G$^+$ neutrophil numbers per field in MMTV-PyMT FVB/NJ mice from indicated groups (n=5/group). Scale bar, 50 μm. Data were analyzed by unpaired *t*-test.
(**G**) Representative H&E images and IF images staining for MPO and H3cit in MCF-7 tumors from indicated groups (n=5/group). Scale bars, 50 μm.
(**H**) Representative H&E images and IF images staining for MPO and H3cit in MMTV-PyMT FVB/NJ tumors from indicated groups (n=5/group). Scale bars, 50 μm.
(**I** and **J**) Representative IF images staining for MPO and H3cit and quantification of NET expression in lung (**I**) and liver (**J**) metastasis of MMTV-PyMT FVB/NJ mice from indicated groups (n=5/group). Scale bars, 50 μm. **I**, Mann-Whitney test. **J**, unpaired *t*-test.
Data are mean ± SD. ***$p < 0.001$, **$p < 0.01$, *$p < 0.05$.

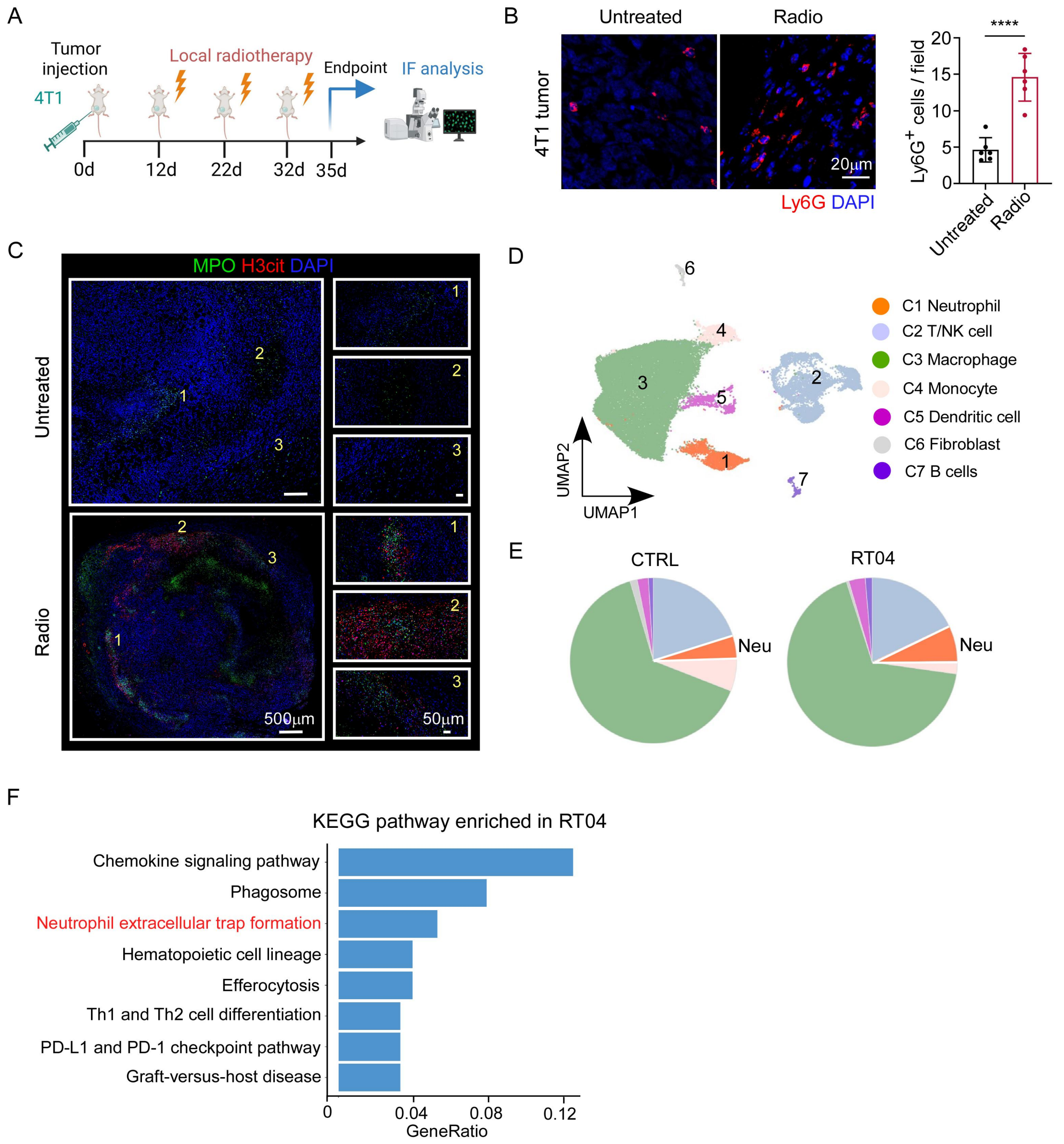

Supplemental Figure 2. Radiotherapy induces neutrophil infiltration and NETosis in breast and lung cancer.

(**A**) Schematic of the local radiotherapy in 4T1 tumor-bearing mice and subsequent IF analysis. Created with BioRender.com.

(**B**) Representative IF images staining for Ly6G and quantification of $Ly6G^+$ cells (n=6/group) in 4T1 tumors from untreated and radiotherapy-treated (radio) groups. Scale bar, 20 μm. Data are mean ± SD. Significance was determined using unpaired *t*-test. ****$p < 0.0001$.

(**C**) Representative IF images staining for MPO and H3cit in 4T1 tumors from untreated and radio groups (n=6/group). Scale bars, 50 μm and 500 μm.

(**D-F**) (**D**) UMAP displays immune cells extracted from CTRL and RT04 groups in public scRNA-seq data (GSA: CRA011692) of Lewis's lung tumors. (**E**) The proportions of immune cell subsets in CTRL and RT04 groups. The red part indicates neutrophil subset. (**F**) KEGG terms associated with upregulated genes in neutrophils isolated from RT04 tumors.

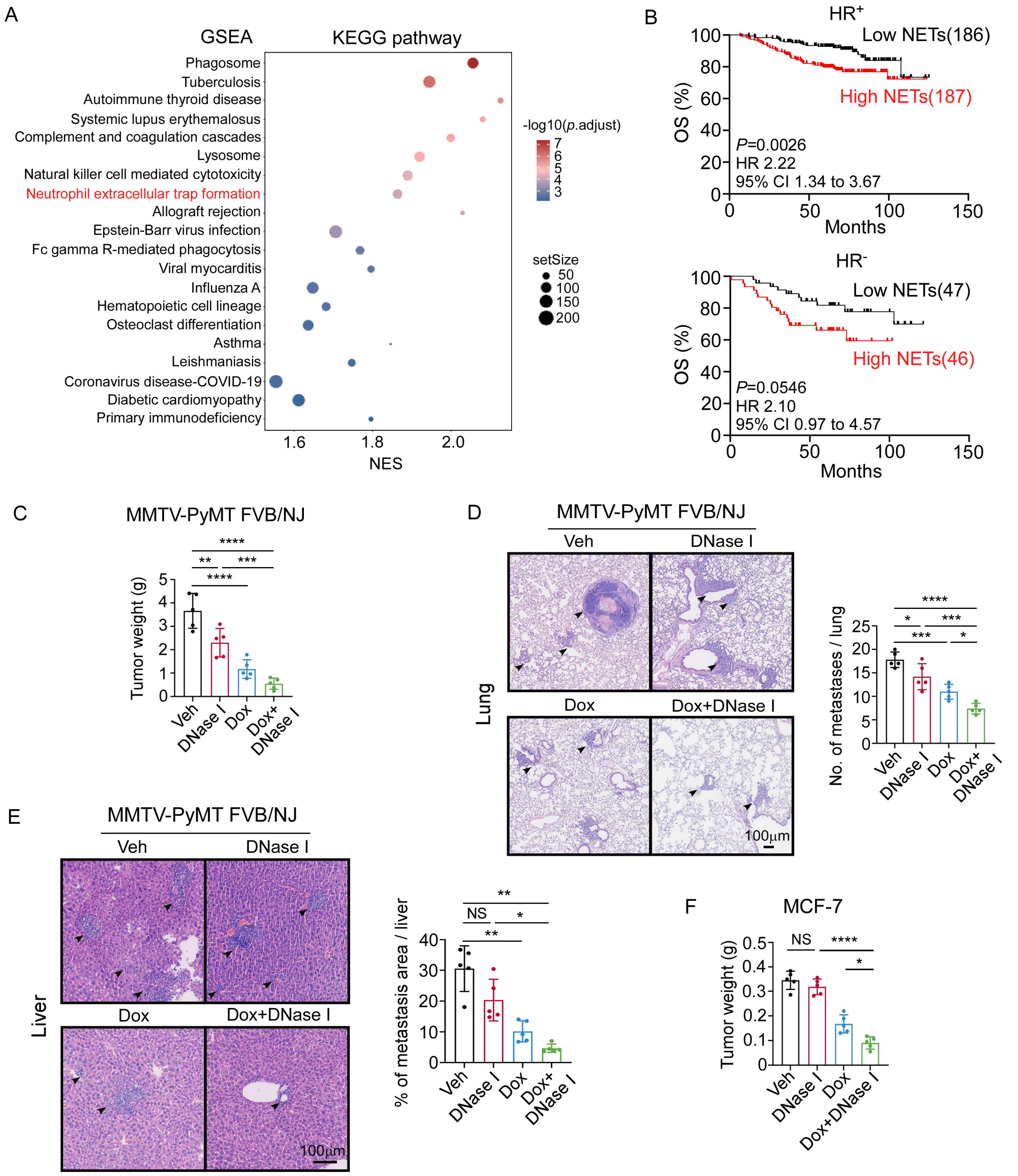

Supplemental Figure 3. NETs correlate with a worse prognosis in breast cancer and compromise the response to chemotherapy in vivo.

(**A**) Bubble heatmap of top 20 GSEA-enriched KEGG terms in breast cancer samples post-chemotherapy in resistant group (GSE18728). Significance was determined by permutation test.

(**B**) Kaplan-Meier survival curves for HR^+ and HR^- breast cancer patients with low (HR^+: n=186, HR^-: n=47) and high (HR^+: n=187, HR^-: n=46) NET levels in primary tumor. Significance was determined using log-rank test.

(**C**) Total tumor weight of transgenic MMTV-PyMT FVB/NJ mice from indicated groups (week 12, n=5/group). Data were analyzed by one-way ANOVA with Tukey's test.

(**D** and **E**) Representative H&E images and quantification of lung metastases (**D**) and

liver metastases (**E**) in MMTV-PyMT FBV/NJ mice with indicated treatments (n=5/group). Scale bars, 100 µm. (**D**), one-way ANOVA with Tukey's test. (**E**), Welch's ANOVA with Dunnett's T3 test.

(**F**) Weight of MCF-7 tumors from indicated groups (n=5/group). Data were analyzed by one-way ANOVA with Tukey's test.

Data are mean ± SD. ****$p < 0.0001$, ***$p < 0.001$, **$p < 0.01$, *$p < 0.05$, NS, not significant.

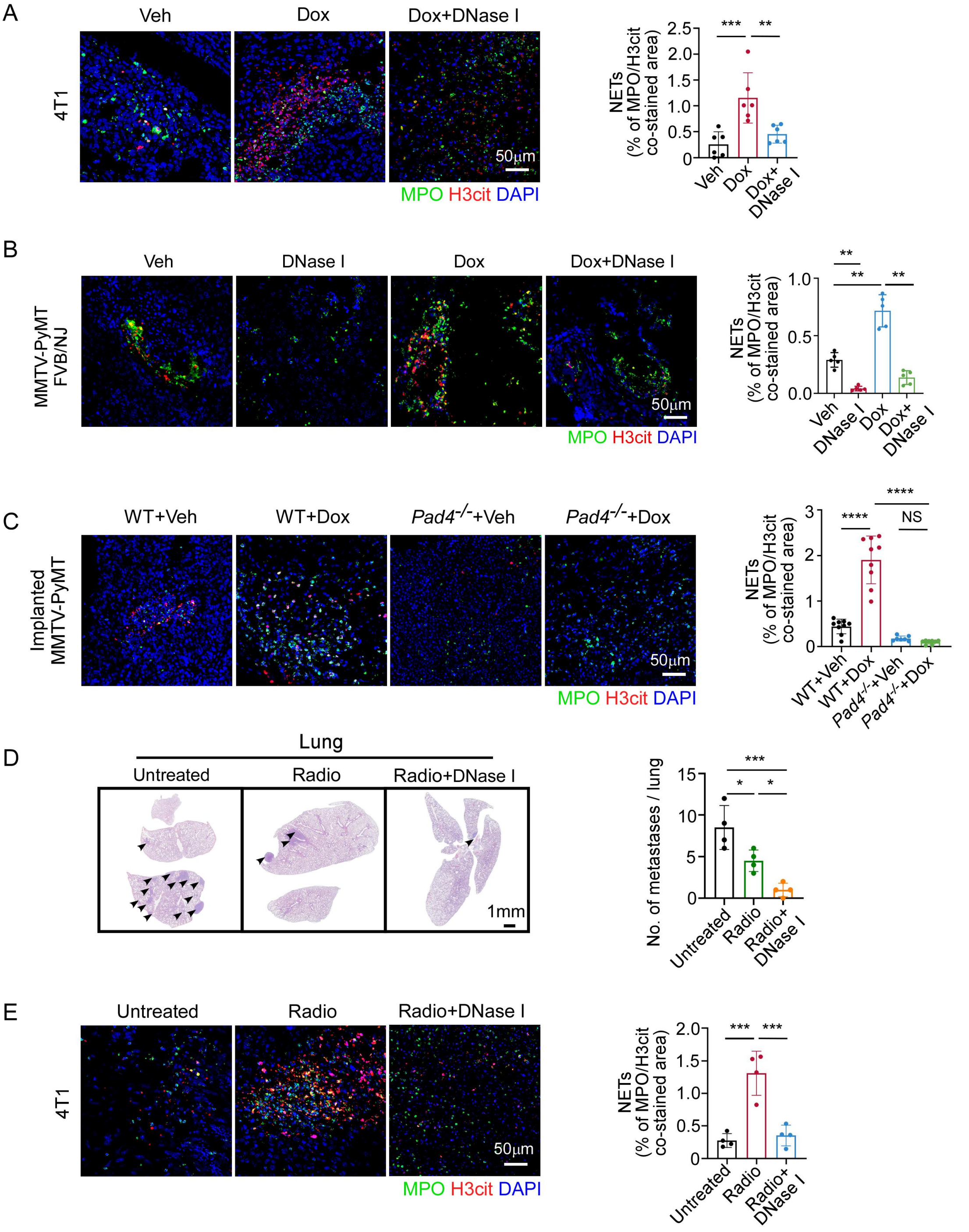

Supplemental Figure 4. Enhanced NET generation impairs the efficacy of chemotherapy and radiotherapy in breast cancer.

(**A**-**C**) Representative IF images staining for MPO and H3cit and quantification of NET expression in 4T1 tumors (n=6/group) (**A**), tumors from MMTV-PyMT FVB/NJ mice (n=5/group) (**B**) and implanted MMTV-PyMT tumors (C57BL/6J background, n=7-9/group) (**C**) with indicated treatments. Scale bars, 50 μm.

(**D**) Representative H&E images and quantification of lung metastases in 4T1 tumor-bearing mice which were untreated, treated with radiotherapy or radiotherapy+DNase I (day 28, n=4/group). Scale bar, 1 mm.

(**E**) Representative IF images staining for MPO and H3cit (left) and quantification of NET expression (right) in 4T1 tumors with indicated treatments (n=4/group). Scale bar, 50 μm.

Data are mean ± SD, significance was determined using one-way ANOVA with Tukey's test (**A**, **D**, **E**), Welch's ANOVA with Dunnett's T3 test (**B**), or two-way ANOVA with Tukey's test (**C**). ****$p < 0.0001$, ***$p < 0.001$, **$p < 0.01$, *$p < 0.05$.

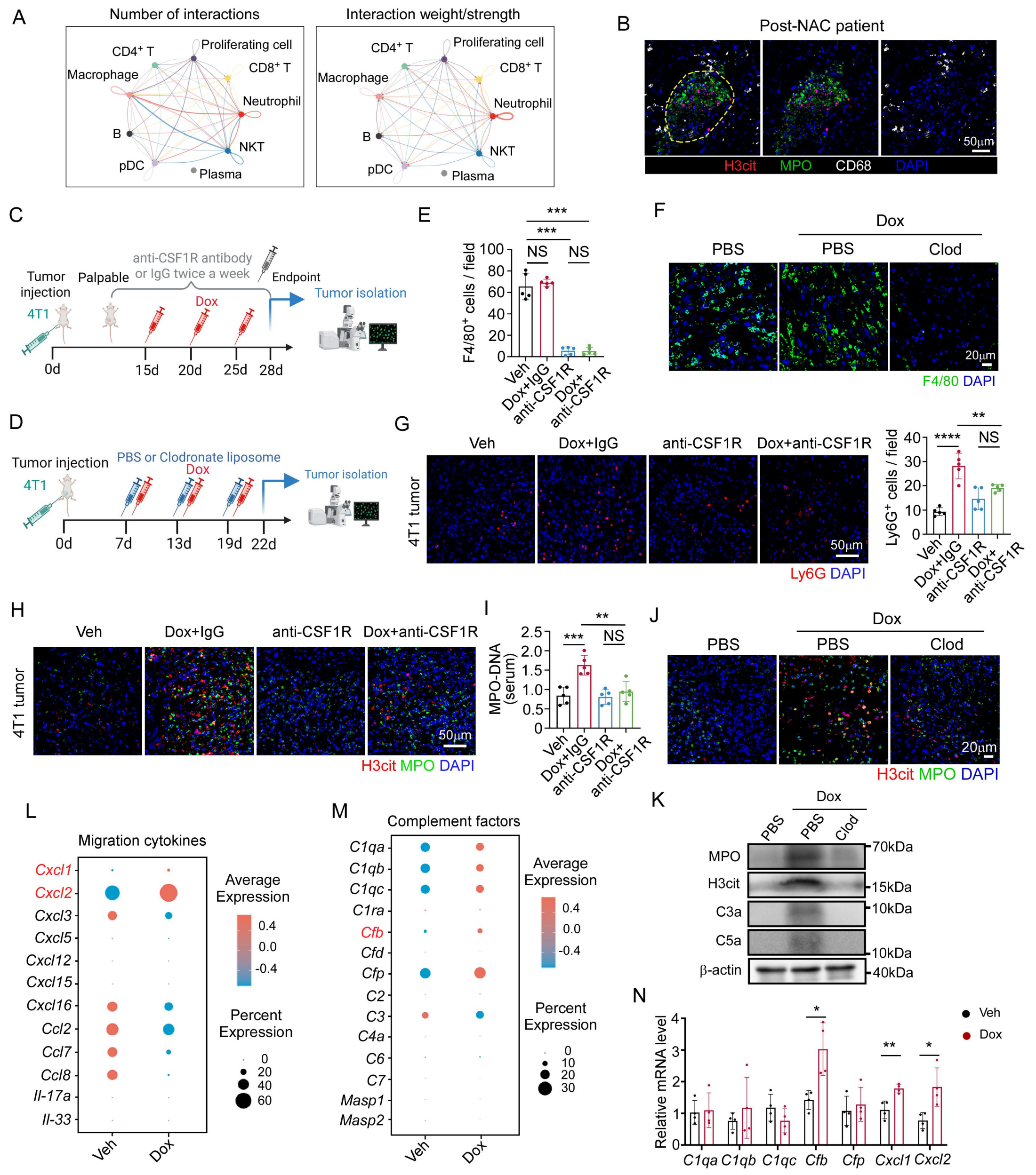

Supplemental Figure 5. Macrophages are indispensable in chemotherapy-induced neutrophil infiltration and NET generation.

(**A**) Circle plots showing the interaction number (left) and weight/strength (right) between indicated cell types in scRNA-seq of 4T1 tumors from Dox-treated mice.

(**B**) Representative IF images staining for MPO, H3cit and CD68 showing spatial distribution of $CD68^+$ macrophages and NETs in post-NAC tumors from breast cancer patients. Data represent 3 independent experiments. Scale bar, 50 μm.

(**C** and **D**) Schematic of 4T1 tumor-bearing mice with indicated treatments. Created with BioRender.com.

(**E**) Quantification of $F4/80^+$ cells per field in 4T1 tumors from indicated groups (n=5/group). Data were analyzed by Welch's ANOVA with Dunnett's T3 test.

(**F**) Representative IF images staining for F4/80 in 4T1 tumors (n=3/group). Data represent 3 independent experiments. Scale bar, 20 μm.

(**G**) Representative IF images showing Ly6G$^+$ neutrophils and quantification in 4T1 tumors from indicated groups (n=5/group). Scale bar, 50 μm. Data were analyzed by one-way ANOVA with Tukey's test.
(**H**) Representative IF images staining for MPO and H3cit in 4T1 tumors from indicated groups (n=5/group). Scale bar, 50 μm.
(**I**) Serum levels of MPO-DNA in mice from indicated groups (n=5/group). Data were analyzed by one-way ANOVA with Tukey's test.
(**J**) Representative IF images staining for MPO and H3cit in 4T1 tumors from indicated groups (n=3/group). Scale bar, 20 μm.
(**K**) Representative western blot showing MPO, H3cit, C3a and C5a expression levels in 4T1 tumors. Data represent 3 independent experiments.
(**L** and **M**) Bubble heatmaps showing the expression levels of migration cytokines (**L**) and complement factors (**M**) that are associated with neutrophil infiltration and NETosis in scRNA-seq of 4T1 tumors.
(**N**) qRT-PCR analysis revealing mRNA levels of *C1qa*, *C1qb*, *C1qc*, *Cfb*, *Cfp*, *Cxcl1* and *Cxcl2* in 4T1 tumor tissues treated with Dox or Veh (n=4/group). Data were analyzed by unpaired *t*-test.
Data are mean ± SD. **** $p < 0.0001$, *** $p < 0.001$, ** $p < 0.01$, * $p < 0.05$.

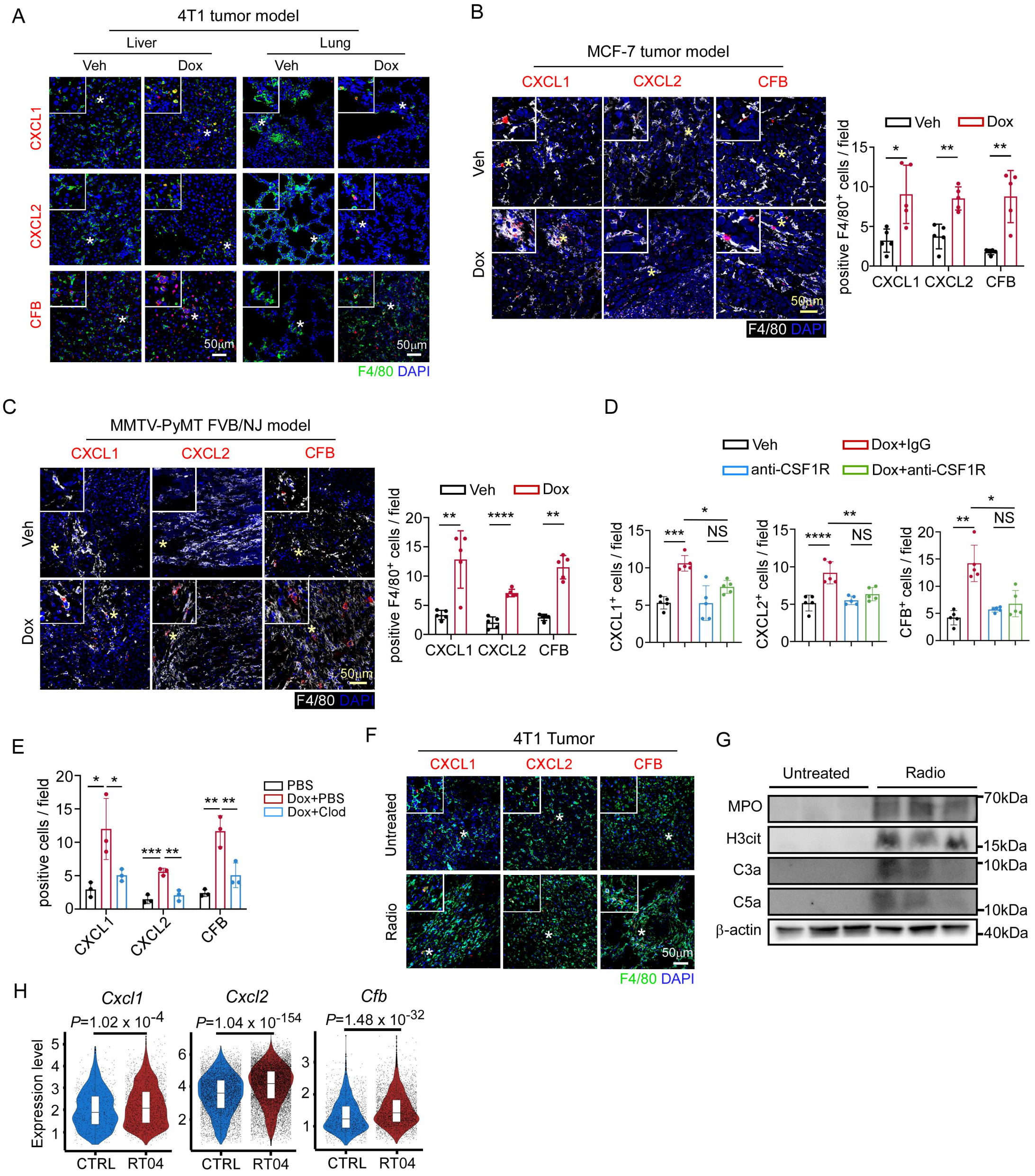

Supplemental Figure 6. Macrophage-derived CXCL1/2 and CFB are upregulated after chemotherapy and radiotherapy.

(**A**) Representative IF images staining for CXCL1/2, CFB and F4/80 in livers and lungs from 4T1-bearing mice treated with Dox or Veh (n=8/group). Scale bars, 50 µm.

(**B**) Representative IF images staining for CXCL1/2, CFB and F4/80 and quantification of CXCL1$^+$ F4/80$^+$, CXCL2$^+$ F4/80$^+$ or CFB$^+$ F4/80$^+$ cells in MCF-7 tumors from mice treated with Dox or Veh (n=5/group). Scale bar, 50 µm. For CXCL1/2, unpaired *t*-test. For CFB, Mann-Whitney test.

(**C**) Representative IF images staining for CXCL1/2, CFB and F4/80 and quantification of CXCL1$^+$ F4/80$^+$, CXCL2$^+$ F4/80$^+$ or CFB$^+$ F4/80$^+$ cells in tumors from MMTV-PyMT FVB/NJ mice with indicated treatments (n=5/group). Scale bar, 50 µm. For CXCL1 and CFB, Mann-Whitney test. For CXCL2, unpaired *t*-test.

(**D**) Quantification of CXCL1$^+$, CXCL2$^+$ and CFB$^+$ cells in 4T1 tumors from indicated

groups (n=5/group). For CXCL1/2, one-way ANOVA with Tukey's test. For CFB, Welch's ANOVA with Dunnett's T3 test.

(**E**) Quantification of $CXCL1^{+}$, $CXCL2^{+}$ and CFB^{+} cells in 4T1 tumors from indicated groups (n=3/group). Data were analyzed by one-way ANOVA with Tukey's test.

(**F**) Representative IF images staining for CXCL1/2, CFB and F4/80 in 4T1 tumors from untreated or radiotherapy group (n=6/group). Scale bar, 50 µm.

(**G**) Representative western blot showing expression levels of MPO, H3cit, C3a and C5a in 4T1 tumors from indicated groups. Data represent 3 independent experiments.

(**H**) Violin plots showing the expression levels of *Cxcl1/2* and *Cfb* in CTRL and RT04 groups from scRNA-seq of lung tumors. Each dot represents a single cell, the lower and upper bounds of boxes indicate the 25th and 75th percentiles, and the middle lines the median values. The whiskers represent the interquartile range. Significance was determined using Welch's *t*-test.

Data are mean ± SD. * in representative images in (**A**-**C**, **F**) indicates the areas that are magnified in the insets in the top-left corners. **** $p < 0.0001$, *** $p < 0.001$, ** $p < 0.01$, * $p < 0.05$.

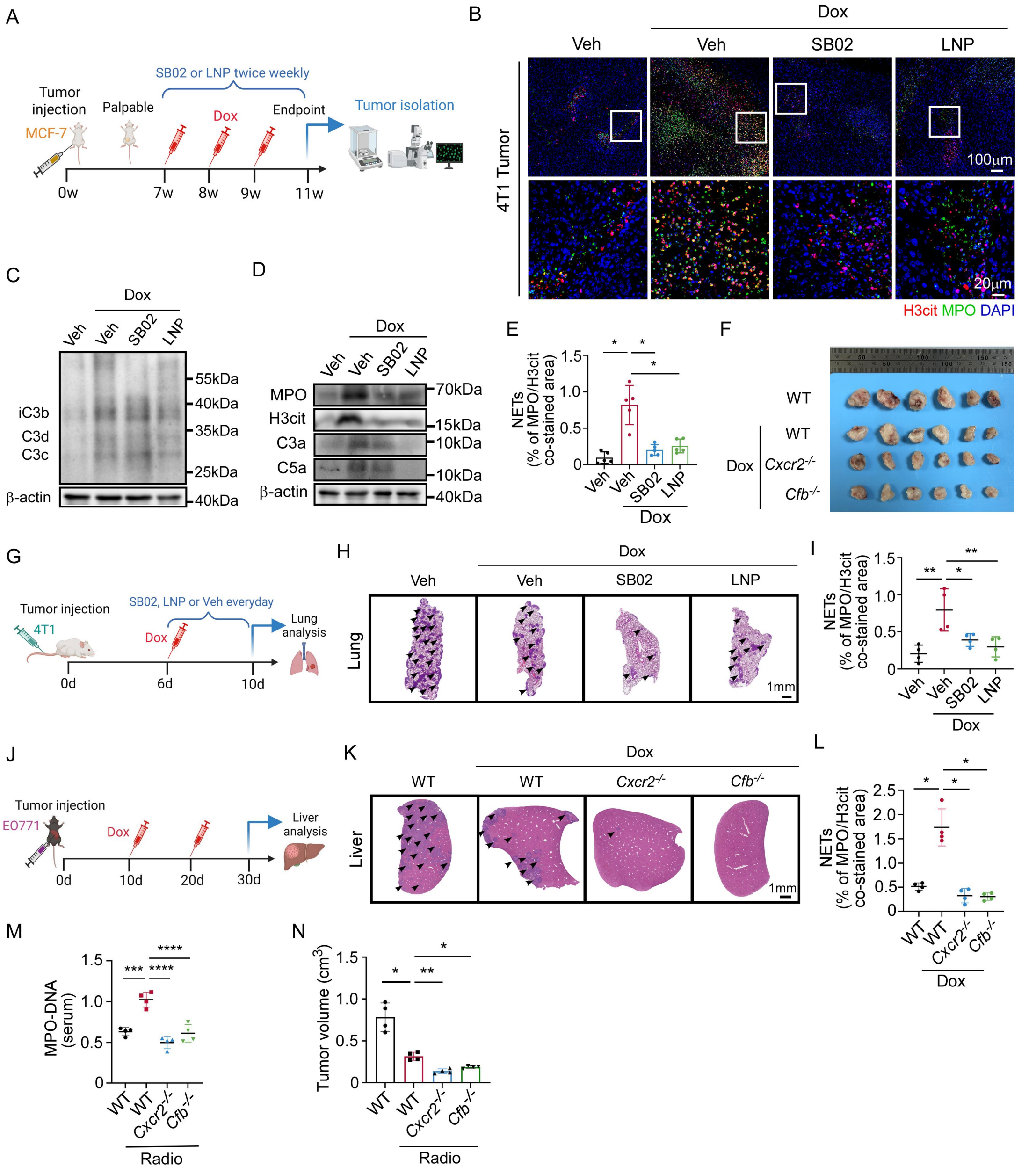

Supplemental Figure 7. Blockade of CXCR2 and CFB inhibits NET generation induced by chemotherapy and radiotherapy.

(**A**) Schematic of MCF-7 tumor-bearing mice with indicated treatments (n=5/group).

(**B**) Representative IF images staining for MPO and H3cit in 4T1 tumors with indicated treatments (n=6/group). The white boxes indicate the areas that are magnified in the next row. Scale bars, 100 μm and 20 μm.

(**C**) Representative western blot showing C3 cleavage products in 4T1 tumors with indicated treatments. Data represent 3 independent experiments.

(**D**) Representative western blot showing MPO, H3cit, C3a and C5a expression levels in 4T1 tumors with indicated treatments. Data represent 3 independent experiments.

(**E**) Quantification of NET expression in MCF-7 tumors from the indicated groups (n=5/group). Data were analyzed by Welch's ANOVA with Dunnett’s T3 test.

(**F**) Representative image of PY8119 tumor size from indicated groups (n=6/group).

(**G**-**I**) Lung metastasis model was conducted by intravenous injection of 4T1 cells in BALB/c mice (n=4/group). (**G**) Schematic of the indicated treatments. (**H**) Representative H&E images showing lung metastases, marked by black arrows. (**I**) Quantification of NET expression in metastatic lungs. Data were analyzed by one-way ANOVA with Tukey's test.
(**J**-**L**) Liver metastasis model was conducted by intrasplenic injection of EO771 tumor cells in C57BL/6 wildtype (WT), *Cxcr2*$^{-/-}$ and *Cfb*$^{-/-}$ mice (n=4/group). (**J**) The experimental schematic is shown. (**K**) Representative H&E images showing liver metastases, marked by black arrows. (**L**) Quantification of NET expression in metastatic livers. Data were analyzed by Welch's ANOVA with Dunnett's T3 test.
(**M** and **N**) WT, *Cxcr2*$^{-/-}$ and *Cfb*$^{-/-}$ mice were inoculated with EO771 tumor cells and treated with or without radiotherapy (n=4/group). Serum levels of MPO-DNA (**M**) and tumor volume (**N**) of the indicated groups are shown. Data were analyzed by one-way ANOVA with Tukey's test (**M**), or Welch's ANOVA with Dunnett's T3 test (**N**).
Data are mean ± SD. Schematics in (**A**, **G**, **J**) were created with BioRender.com. **** $p < 0.0001$, *** $p < 0.001$, ** $p < 0.01$, * $p < 0.05$.

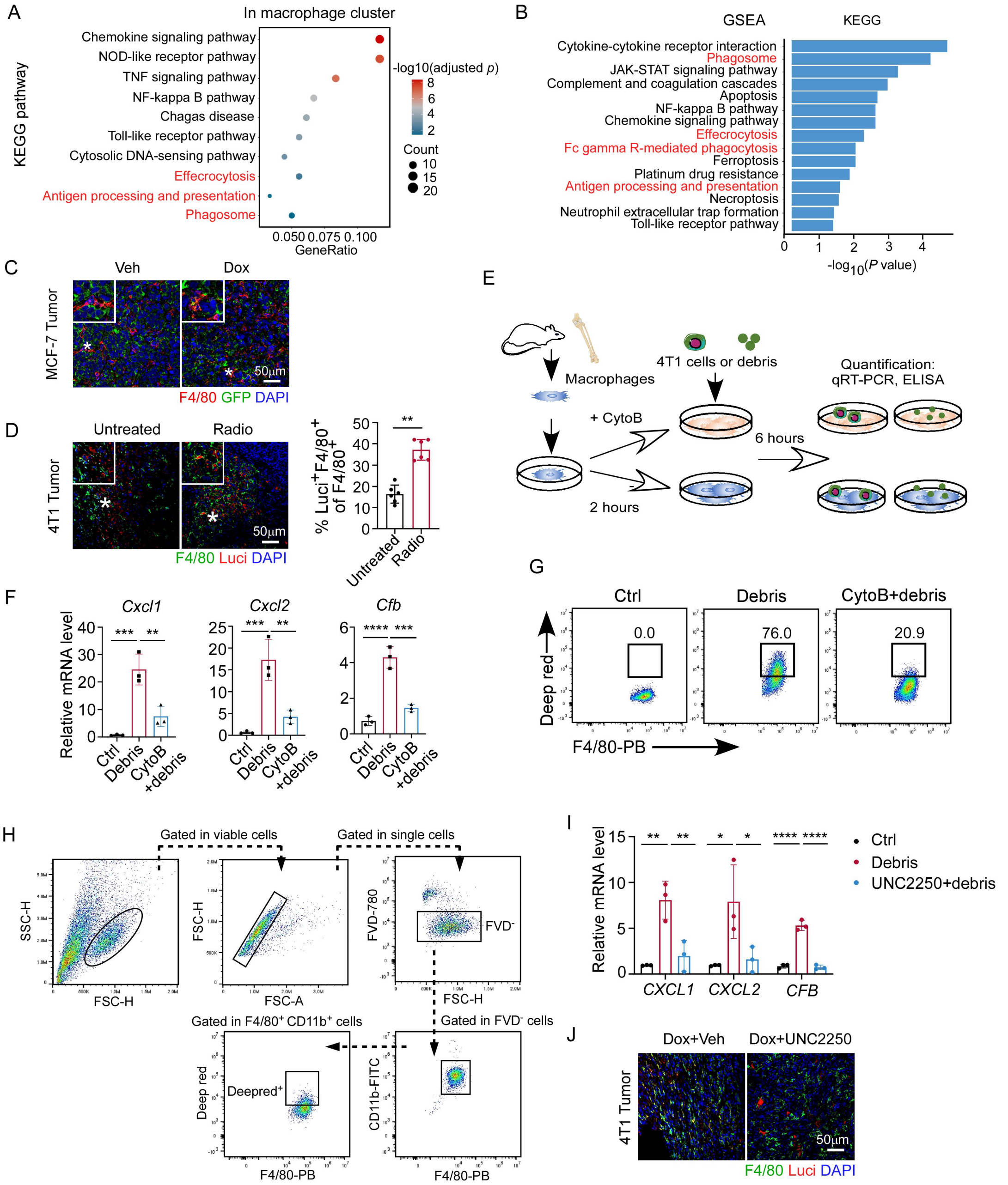

Supplemental Figure 8. Macrophages promote CXCL1/2 and CFB elevation and NETosis via phagocytosis of treatment-induced tumor debris.

(**A**) Bubble heatmap showing the KEGG pathway enrichment of the upregulated genes in macrophages from lung tumors treated by radiotherapy, compared to those from untreated lung tumors (hypergeometric test).

(**B**) GSEA revealing the enriched KEGG pathways in RNA-seq of breast tumors post-radiotherapy from the GSE59733 dataset (permutation test).

(**C**) Representative IF images staining for F4/80 and GFP in MCF-7 tumors from indicated groups (n=5/group). Scale bar, 50 μm.

(**D**) Representative IF images staining for F4/80 and Luci (left) and quantification (right) of the percentage of $Luci^+$ $F4/80^+$ cells in $F4/80^+$ cells in 4T1 tumors from indicated groups (n=6/group). Scale bar, 50 μm. Data were analyzed by Mann-Whitney test.

(**E**) Bone marrow-derived macrophages (BMDMs) were pretreated with or without cytochalasin B (CytoB), and co-cultured with intact 4T1 cells or debris in vitro, followed by qRT-PCR or ELISA experiments. The experimental schematic is shown.
(**F**) Transcriptional levels of *Cxcl1/2* and *Cfb* in BMDMs from indicated groups (n=3/group). Data were analyzed by one-way ANOVA with Tukey's test.
(**G**) Representative flow plots showing the phagocytosis of 4T1 cell debris (labeled by Deep red) by F4/80$^+$ CD11b$^+$ BMDMs in indicated groups.
(**H**) Flow cytometry showing the gating strategy of Deep red$^+$ macrophages in (**G**). Data represent 3 independent experiments in (**G** and **H**).
(**I**) Transcriptional levels of *CXCL1/2* and *CFB* in human macrophages from indicated groups (n=3/group). Data were analyzed by one-way ANOVA with Tukey's test.
(**J**) Representative IF images staining for F4/80 and Luci in 4T1 tumors from indicated groups (n=5/group). Scale bar, 50 µm.
Data are mean ± SD. The white * in images in (**C** and **D**) indicates the areas that are magnified in the insets in the top-left corners. ****$p < 0.0001$, *** $p < 0.001$, ** $p < 0.01$, * $p < 0.05$.

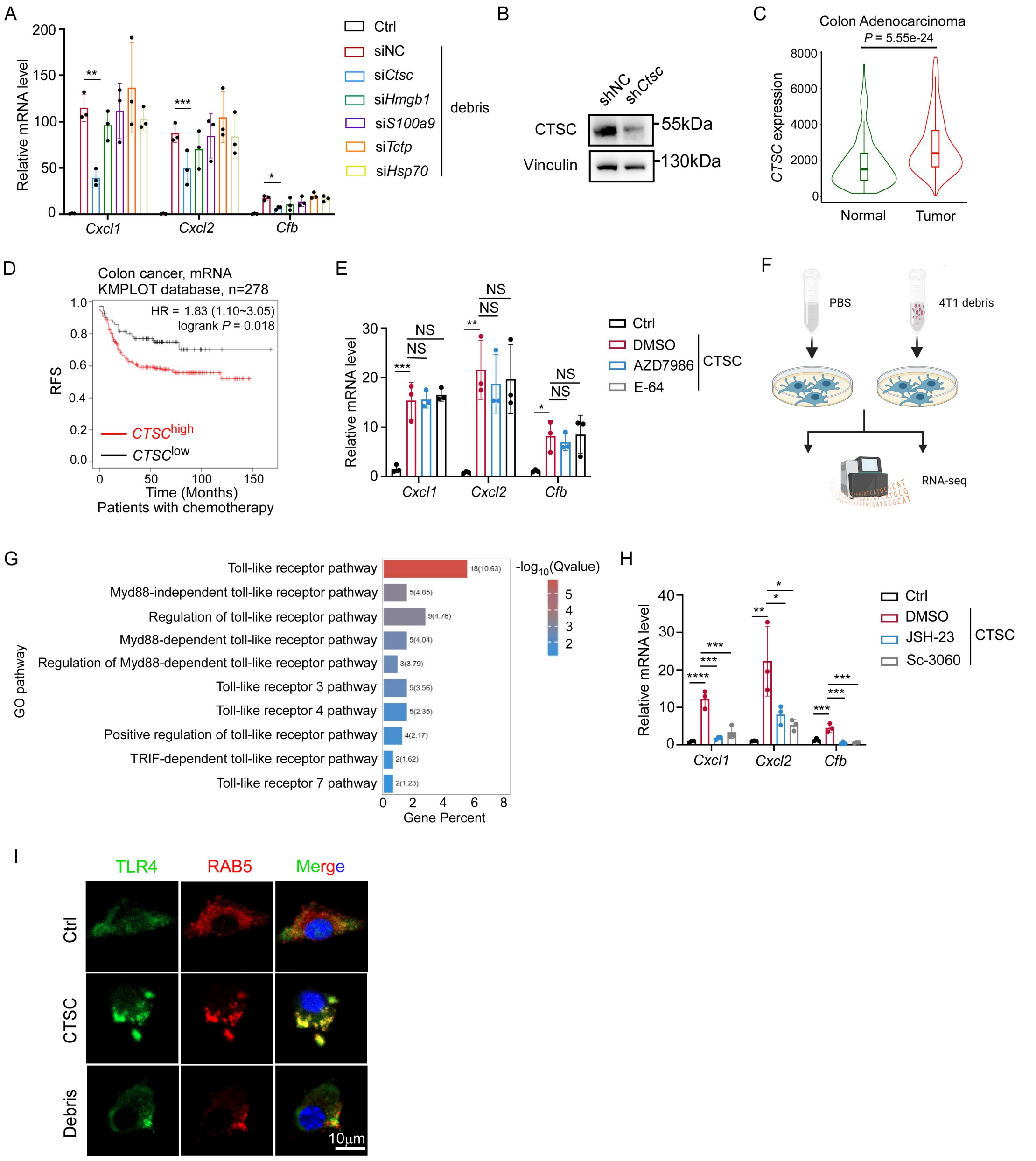

Supplemental Figure 9. The protein CTSC in tumor debris contributes to chemotherapy-induced NETosis.

(**A**) qRT-PCR for *Cxcl1/2* and *Cfb* mRNA expression in BMDMs with indicated treatments (n=3/group). Significance was determined using one-way ANOVA with Dunnett's test.

(**B**) Representative western blot showing *Ctsc* knockdown efficacy in 4T1 tumor cells. Data represent 3 independent experiments.

(**C**) Violin plot showing *CTSC* expression in colon adenocarcinoma and normal colon tissues from the TNMplot database (normal: n=315; tumor: n=469). Each dot represents a sample, the lower and upper bounds of boxes indicate the 25th and 75th percentiles, and the middle lines the median values. The whiskers represent the interquartile range. Significance was determined by Mann-Whitney test.

(**D**) Kaplan-Meier curves showing recurrence-free survival (RFS) in sub-stratified

patients with colon cancer receiving chemotherapy with high (n=207) and low (n=71) *CTSC* expression from the KMPLOT database. Significance was determined by log-rank test.

(**E**) qRT-PCR for *Cxcl1/2* and *Cfb* mRNA expression in BMDMs with indicated treatments (n=3/group). Data were analyzed by one-way ANOVA with Tukey's test.

(**F**) Experimental schematic: BMDMs were treated with 4T1 tumor debris or PBS control in vitro and RNA-seq was performed. Created with BioRender.com.

(**G**) GO pathway enrichment analysis of DEGs in 4T1 debris-treated macrophages in RNA-seq (hypergeometric test).

(**H**) qRT-PCR for *Cxcl1/2* and *Cfb* mRNA expression in BMDMs with indicated treatments (n=3/group). Data were analyzed by one-way ANOVA with Tukey's test.

(**I**) Representative images staining for TLR4 and RAB5 in BMDMs (n=5/group). Scale bar, 10 μm.

Data are mean ± SD. **** $p < 0.0001$, *** $p < 0.001$, ** $p < 0.01$, * $p < 0.05$.

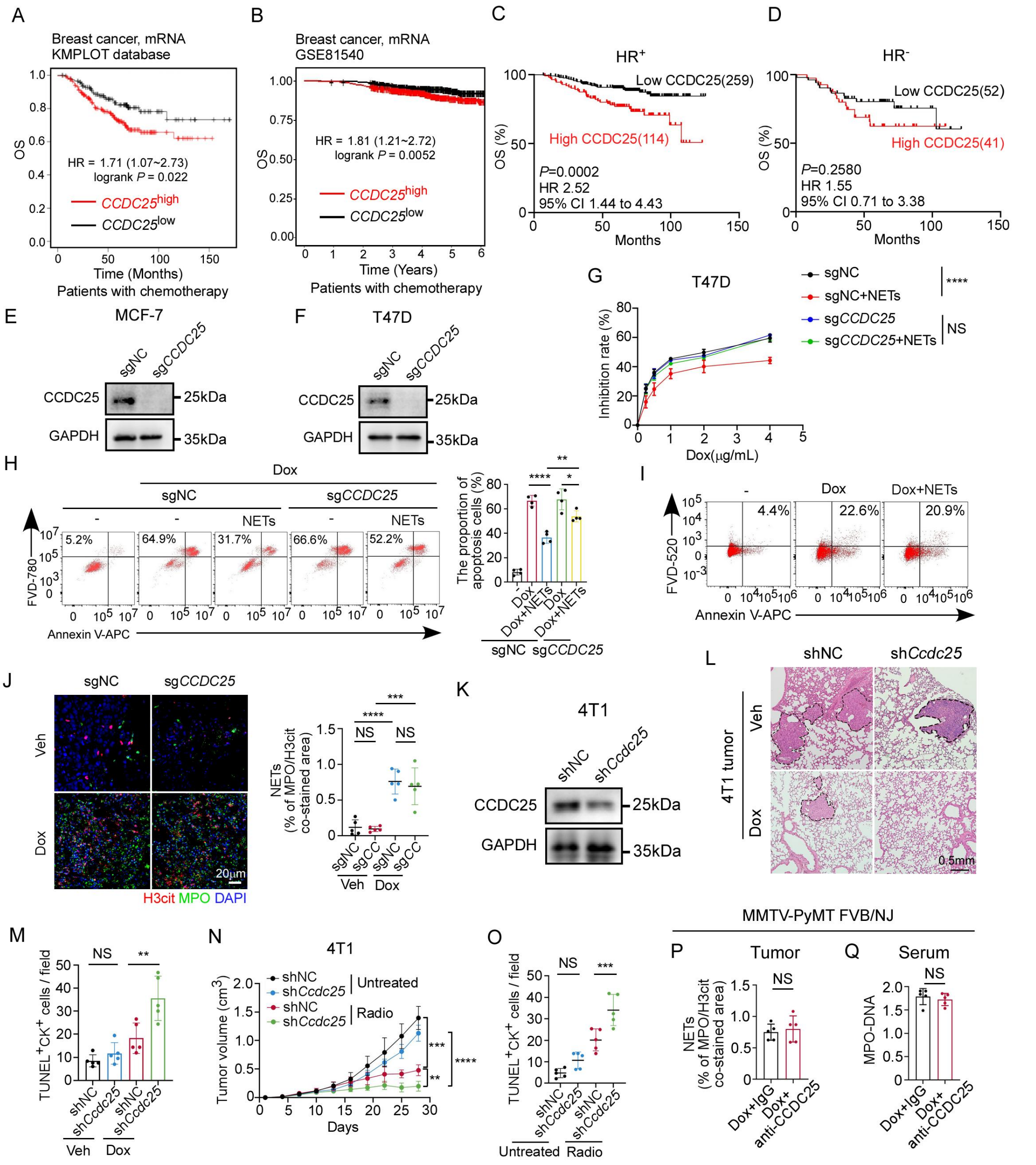

Supplemental Figure 10. NETs promote therapeutic resistance of breast cancer via CCDC25.

(**A** and **B**) Kaplan-Meier curves showing OS in sub-stratified patients with breast cancer received chemotherapy with high and low *CCDC25* expression from the KMPLOT (**A**) and GSE81540 (**B**) datasets. Significance was determined by log-rank test.

(**C** and **D**) Kaplan-Meier curves revealing OS for HR^+ (**C**) and HR^- (**D**) breast cancer patients with low (HR^+: n=259, HR^-: n=52) and high (HR^+: n=114, HR^-: n=41) tumoral CCDC25 expression. Significance was determined using log-rank test.

(**E** and **F**) Representative western blot showing *CCDC25* knockout efficacy in MCF-7 (**E**) and T47D (**F**) cells.

(**G**) The growth inhibition rates of Dox on sgNC or sg*CCDC25* T47D cells pre-treated with or without NETs (n=3/group). Data were analyzed by two-way ANOVA with Sidak's test. Data were compared at 4 μg/mL Dox.

(**H**) Quantification of the proportions of apoptotic T47D tumor cells in different groups by flow cytometry (n=4/group). Data were analyzed by one-way ANOVA with Tukey's test.
(**I**) The proportions of apoptotic MDA-MB-231 cells cultured alone (-), treated with Dox or Dox combined with NETs.
(**J**) Representative IF images and quantification of NET expression in sgNC or sg*CCDC25* MCF-7 tumors with indicated treatments (n=5/group). Scale bar, 20 μm. Data were analyzed by two-way ANOVA with Tukey's test.
(**K**) Representative western blot showing *Ccdc25* knockdown efficacy in 4T1 cells.
(**L**) Representative H&E images showing lung metastases in indicated groups of 4T1-bearing mice (n= 5/group). The black lines indicate the lung metastatic nodules. Scale bar, 0.5 mm.
(**M**) Statistical analysis of $TUNEL^+$ CK^+ cells in 4T1 tumors from indicated groups (n=5/group). Data were analyzed by two-way ANOVA with Sidak's test.
(**N** and **O**) Tumor growth curves (**N**) and statistical analysis of $TUNEL^+$ CK^+ cells (**O**) in shNC or sh*Ccdc25* 4T1 tumors untreated or treated with radiotherapy (n=5/group). Data were analyzed by two-way repeated measures ANOVA with Tukey's test and compared at day 28 (**N**), or two-way ANOVA with Sidak's test (**O**).
(**P** and **Q**) Statistical analysis of tumoral NET expression (**P**) and serum MPO-DNA levels (**Q**) in MMTV-PyMT FVB/NJ mice from indicated groups (n=5/group). For **P**, unpaired *t*-test. For **Q**, Mann-Whitney test.
Data are mean ± SD. Data represent 3 independent experiments in (**E**, **F**, **I**, **K**). **** $p < 0.0001$, *** $p < 0.001$, ** $p < 0.01$, * $p < 0.05$.

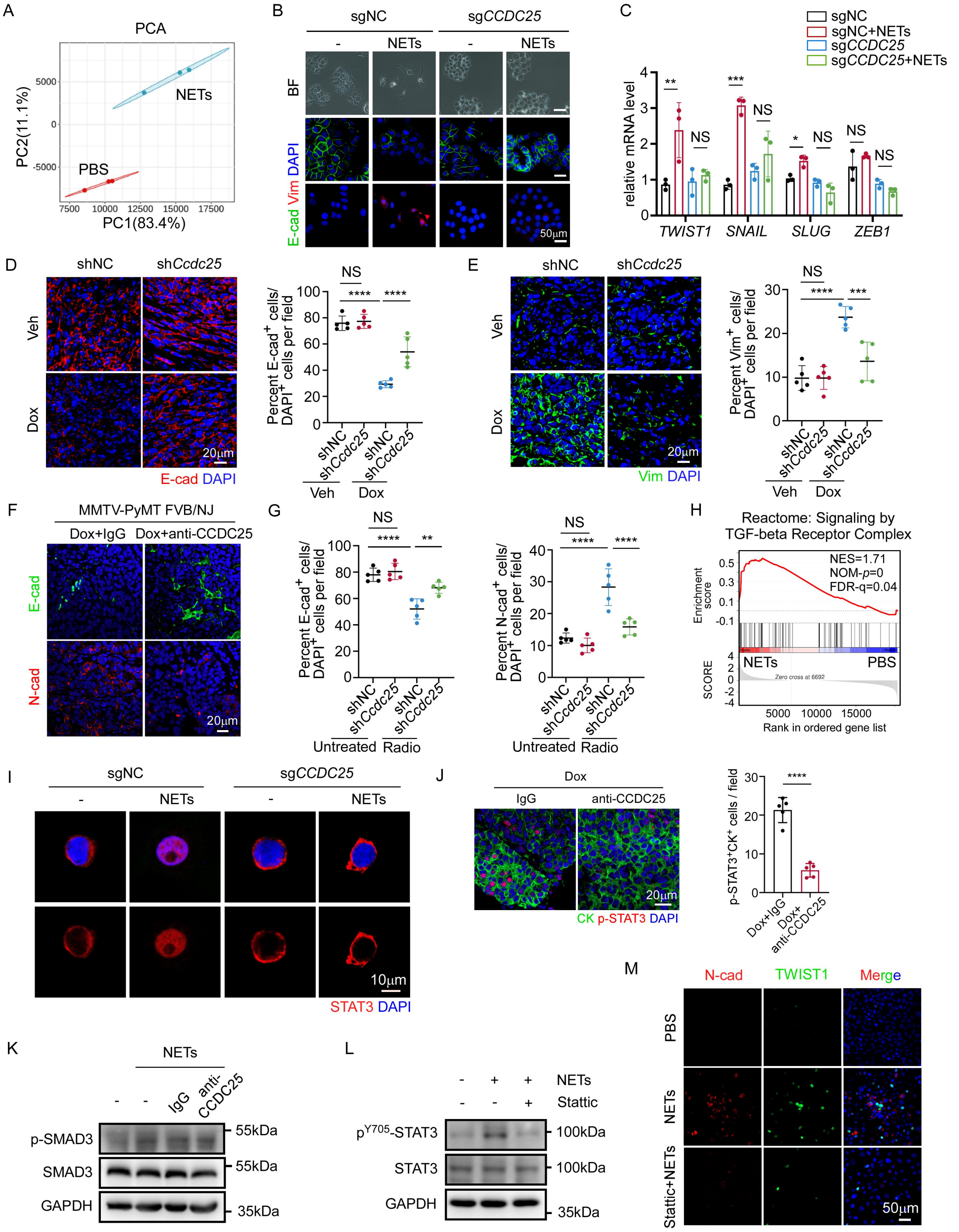

Supplemental Figure 11. NET-CCDC25 interaction promotes tumor cell chemoresistance via STAT3 activation and EMT.

(**A**) Principal component analysis (PCA) of RNA-seq of MCF-7 cells treated with NETs or PBS.

(**B**) Representative bright field (BF) images and IF images staining for E-cad and Vim in sgNC or sg*CCDC25* MCF-7 cells treated with (+) or without (-) NETs in vitro. Scale bars, 50 μm.

(**C**) qRT-PCR analysis for *TWIST1*, *SNAIL*, *SLUG* and *ZEB1* mRNA expression in sgNC or sg*CCDC25* MCF-7 cells treated with or without NETs (n=3/group).

(**D**) Representative IF images staining for E-cad and quantification of E-cad^{+} cell proportions (n=5/group) in shNC or sh*Ccdc25* 4T1 tumors treated with Dox or Veh. Scale bar, 20 μm.
(**E**) Representative IF images staining for Vim and quantification of Vim^{+} cell proportions (n=5/group) in shNC or sh*Ccdc25* 4T1 tumors treated with Dox or Veh. Scale bar, 20 μm.
(**F**) Representative IF images staining for E-cad and N-cad in tumors from MMTV-PyMT FVB/NJ mouse model with indicated treatments (n=5/group). Scale bar, 20 μm.
(**G**) Statistical analysis of proportions of E-cad^{+} (left) and N-cad^{+} cells (right) in shNC or sh*Ccdc25* 4T1 tumors untreated or treated with radiotherapy (n=5/group).
(**H**) GSEA showing that Reactome signaling by TGF-beta receptor complex is enriched in RNA-seq from NET-treated MCF-7 cells.
(**I**) Representative IF images staining for STAT3 in sgNC or sg*CCDC25* MCF-7 cells treated with or without NETs in vitro. Scale bar, 10 μm.
(**J**) Representative IF images staining for CK and p^{Y705}-STAT3 and quantification of p-STAT3^{+}CK^{+} cells in tumors from MMTV-PyMT FVB/NJ mice with indicated treatments (n=5/group). Scale bar, 20 μm.
(**K**) Representative western blot showing p-SMAD3 and SMAD3 expression levels in MCF-7 cells with indicated treatments.
(**L**) Representative western blot showing p^{Y705}-STAT3 and STAT3 expression levels in MCF-7 cells with indicated treatments.
(**M**) Representative IF images staining for N-cad and TWIST1 in MCF-7 cells from indicated groups. Scale bar, 50 μm.
Data are mean ± SD, significance was determined using two-way ANOVA with Sidak's test (**C**-**E**, **G**), permutation test (**H**), or unpaired *t*-test (**J**). Data represent 3 independent experiments in (**B**, **I**, **K**-**M**). **** $p < 0.0001$, *** $p < 0.001$, ** $p < 0.01$, * $p < 0.05$.

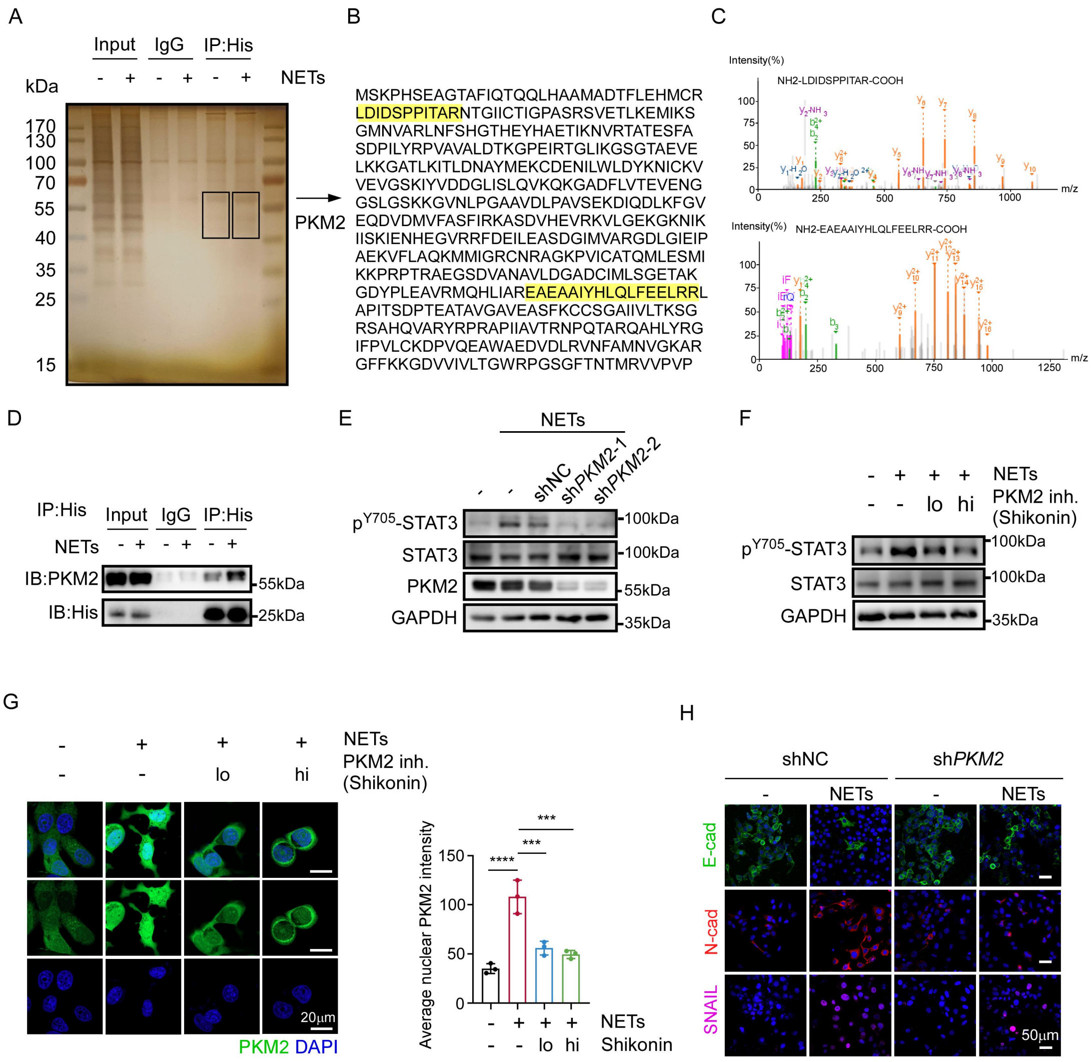

Supplemental Figure 12. PKM2 works downstream of NET-CCDC25 signaling to induce STAT3 phosphorylation and EMT.

(**A**) Cytosolic extracts from the MCF-7 cells that were transfected with His-tagged-CCDC25 were treated with (+) or without (−) NETs and immunoprecipitated using anti-His antibody. Bound proteins were eluted, separated by western blot and visualized by silver staining. The precipitated protein bands within a molecular weight range of 40-70 kDa were submitted for mass spectrometry (black boxes).

(**B**) The full amino-acid sequence of human PKM2. The sequences in yellow are the tryptic peptides identified by liquid chromatography-mass spectrometry.

(**C**) Mass spectrometry analysis of the protein bands highlighted in (**B**).

(**D**) Immunoblotting (IB) of PKM2 and His-CCDC25 in the lysates (input) or immunoprecipitates (IP; IgG or anti-His) of MCF-7 cells stimulated with (+) or without (−) NET treatment.

(**E**) The protein levels of p^{Y705}-STAT3, total STAT3 and PKM2 were examined in the lysates of MCF-7 cells, either untreated or transduced with NC or *PKM2* shRNAs and stimulated with or without NETs.

(**F**) Western blots for p^{Y705}-STAT3 and total STAT3 expression in MCF-7 cells that were untreated (-) or treated with (+) NETs, along with low (lo), high (hi) or no treatment of PKM2 inhibitor, Shikonin.
(**G**) (Left) Representative immunostaining images of PKM2 and DAPI of MCF-7 cells which were untreated (-) or treated with (+) NETs, along with low (lo), high (hi) or no treatment of Shikonin. Scale bars, 20 μm. (Right) Quantification of the average nuclear PKM2 intensity in indicated groups (n=3/group). Data are mean ± SD, significance was determined using one-way ANOVA with Tukey's test. **** $p < 0.0001$, *** $p < 0.001$.
(**H**) Representative IF images of E-cad, N-cad and SNAIL of MCF-7 cells transduced with NC or *PKM2* shRNAs and stimulated with (+) or without (-) NETs. Scale bars, 50 μm.
Data represent 3 independent experiments in (**D**-**H**).

Supplemental Tables

Supplemental Table 1

Univariate and multivariable Cox's regression analysis on our patient cohort

	**Univariate analysis**			
	**Cox regression-DFS**		**Cox regression-OS**	
	**HR (95% CI)**	***p*-value**	**HR (95% CI)**	***p*-value**
Age at diagnosis				
≤40	1.00 (reference)		1.00 (reference)	
41–59	0.75 (0.53-1.09)	0.122	1.11 (0.68-1.88)	0.688
≥60	0.82 (0.46-1.40)	0.484	1.47 (0.72-2.90)	0.278
HR status				
Negative	1.00 (reference)		1.00 (reference)	
Positive	0.63 (0.44-0.92)	0.015	0.51 (0.33-0.83)	0.005
Clinical stage				
I/II	1.00 (reference)		1.00 (reference)	
III	2.07 (1.49-2.89)	<0.001	2.45 (1.59-3.81)	<0.001
NET level (post-NAC)	3.50 (2.14-5.25)	<0.001	3.47 (1.99-5.40)	<0.001
	**Multivariable analysis**			
	**Cox regression-DFS**		**Cox regression-OS**	
	**HR (95% CI)**	***p*-value**	**HR (95% CI)**	***p*-value**
NET level (post-NAC)	3.41 (2.09-5.13)	<0.001	3.62 (2.06-5.71)	<0.001

HR, hazard ratio. CI, confidence interval.

Supplemental Table 2

The siRNA sequences

Gene name	siRNA	Sequence
Tlr4 siRNA1	**sense**	5’-CUUCUUCAACCAAGAACAU-3’
	**anti-sense**	5’-AUGUUCUUGGUUGAAGAAG-3’
Tlr4 siRNA2	**sense**	5’-CAAUUGACUUCAUUCAAGA-3’
	**anti-sense**	5’-UCUUGAAUGAAGUCAAUUG-3’
Myd88 siRNA1	**sense**	5’-GGAGAUGAUCCGGCAACUA-3’
	**anti-sense**	5’-UAGUUGCCGGAUCAUCUCC-3’
Myd88 siRNA2	**sense**	5’-CUAUAUGCGACUAUACCAA-3’
	**anti-sense**	5’-UUGGUAUAGUCGCAUAUAG-3’
Trif siRNA1	**sense**	5’-GGAUCGGUGCAGUUCAGAU -3’
	**anti-sense**	5’-AUCUGAACUGCACCGAUCC-3’
Trif siRNA2	**sense**	5’-GGGUUACCACACGAAAUUA-3’
	**anti-sense**	5’-UAAUUUCGUGUGGUAACCC-3’
S100a9 siRNA1	**sense**	5’-GCAAGAAGGAAUUCAGACA-3’
	**anti-sense**	5’-UGUCUGAAUUCCUUCUUGC-3’
S100a9 siRNA2	**sense**	5’-CAGAUGGAGCGCAGCAUAA-3’
	**anti-sense**	5’-UUAUGCUGCGCUCCAUCUG-3’
Hsp70 siRNA1	**sense**	5’-CCAAGGUGCAGGUGAACUA-3’
	**anti-sense**	5’-UAGUUCACCUGCACCUUGG-3’
Hsp70 siRNA2	**sense**	5’-GGCAUCGACUUCUACACAU-3’
	**anti-sense**	5’-AUGUGUAGAAGUCGAUGCC-3’
Tctp siRNA1	**sense**	5’-CAUGAACCAUCACUUACAA-3’
	**anti-sense**	5’-UUGUAAGUGAUGGUUCAUG-3’
Tctp siRNA2	**sense**	5’-GGCAAGAUGGUCAGUAGAA-3’
	**anti-sense**	5’-UUCUACUGACCAUCUUGCC-3’
Hmgb1 siRNA1	**sense**	5’-UGUUGUUCCACAUCUCUCC-3’
	**anti-sense**	5’-GGAGAGAUGUGGAACAACA-3’
Hmg*b1* siRNA2	**sense**	5’-UGUUACCCUACCACAAUGG-3’
	**anti-sense**	5’-CCAUUGUGGUAGGGUAACA-3’
Ctsc siRNA1	**sense**	5’-CACACAGCUAUCAGUUACU-3’
	**anti-sense**	5’-AGUAACUGAUAGCUGUGUG-3’
Ctsc siRNA2	**sense**	5’-GCAACUGCAUAUAAGGAAU-3’
	**anti-sense**	5’-AUUCCUUAUAUGCAGUUGC-3’
NC siRNA	**sense**	5’-UUCUCCGACAGUGUCACGU-3’
	**anti-sense**	5’-ACGUGACACUGUCGGAGAA-3’

Supplemental Table 3

The primer sequences

**Gene name**	**Primer sequence**
Cxcl1-F Mouse	5'-CTGGGATTCACCTCAAGAACATC-3'
Cxcl1-R Mouse	5'-CAGGGTCAAGGCAAGCCTC-3'
Cxcl2-F Mouse	5'-CCAACCACCAGGCTACAGG-3'
Cxcl2-R Mouse	5'-GCGTCACACTCAAGCTCTG-3'
Cfb-F Mouse	5'-TACCCCGTGCAGACTCGAA-3'
Cfb-R Mouse	5'-GTGGGCAGCGTATTGCTCT-3'
C1qa-F Mouse	5'- AAAGGCAATCCAGGCAATATCA-3'
C1qa-R Mouse	5'- TGGTTCTGGTATGGACTCTCC-3'
C1qb-F Mouse	5'-GAGGTCTGGACACACCTGTTA-3'
C1qb-R Mouse	5'-CTCCCCTTTAATCCCTGGAGT-3'
C1qc-F Mouse	5'-AGAAGCACCAGTCGGTATTCA-3'
C1qc-R Mouse	5'-TGCGATGTGTAGTAGACGAAGTA-3'
Cfp-F Mouse	5'-CCCTTTTGTGCTGGTGATGC-3'
Cfp-R Mouse	5'-CTCCCACTCCCCGTTTACAG-3'
Gapdh-F Mouse	5'- AGGTCGGTGTGAACGGATTTG-3'
Gapdh-R Mouse	5'- TGTAGACCATGTAGTTGAGGTCA-3'
TWIST1-F Human	5'-GTCCGCAGTCTTACGAGGAG-3'
TWIST1-R Human	5'-GCTTGAGGGTCTGAATCTTGCT-3'
SNAIL-F Human	5'-TCGGAAGCCTAACTACAGCGA-3'
SNAIL-R Human	5'-AGATGAGCATTGGCAGCGAG-3'
SLUG-F Human	5'- CGAACTGGACACACATACAGTG -3'
SLUG-R Human	5'-CTGAGGATCTCTGGTTGTGGT-3'
ZEB1-F Human	5'-TTACACCTTTGCATACAGAACCC-3'
ZEB1-R Human	5'-TTTACGATTACACCCAGACTGC-3'
CXCL1-F Human	5'-TTGTGAAGGCAGGGGAATGT-3'
CXCL1-R Human	5'-AAGCCCCTTTGTTCTAAGCCA-3'
CXCL2-F Human	5'-CGGCAGGGAAATGTATGTGTG-3'
CXCL2-R Human	5'-TCGAAACCTCTCTGCTCTAACA-3'
CFB-F Human	5'-CGTGTGTCCTTCTGGCTTCT-3'
CFB-R Human	5'-CGAAGTCGTGTGGTCTTGGA-3'
GAPDH-F Human	5'-AGGTCGGTGTGAACGGATTTG-3'
GAPDH-R Human	5'-TGTAGACCATGTAGTTGAGGTCA-3'

Supplemental reference

1. Yang L, et al. DNA of neutrophil extracellular traps promotes cancer metastasis via CCDC25. *Nature.* 2020;583(7814):133-8.
2. Nie M, et al. Cardiomyocyte-localized CCDC25 senses NET DNA to promote doxorubicin cardiotoxicity by activating autophagic flux. *Nat Cancer.* 2025;6(8):1400-1418.
3. Astuti Y, et al. Efferocytosis reprograms the tumor microenvironment to promote pancreatic cancer liver metastasis. *Nat Cancer.* 2024;5(5):774-790.
4. Lhuillier C, et al. Radiotherapy-exposed CD8+ and CD4+ neoantigens enhance tumor control. *J Clin Invest.* 2021;131(5):e138740.
5. Chang J, et al. Chemotherapy-generated cell debris stimulates colon carcinoma tumor growth via osteopontin. *FASEB J.* 2019;33(1):114-25.
6. Chen X, et al. Long noncoding RNA DIO3OS induces glycolytic-dominant metabolic reprogramming to promote aromatase inhibitor resistance in breast cancer. *Nat Commun.* 2022;13(1):7160.
7. Liu X, et al. CD16(+) fibroblasts foster a trastuzumab-refractory microenvironment that is reversed by VAV2 inhibition. *Cancer Cell.* 2022;40(11):1341-57 e13.
8. Sulciner ML, et al. Resolvins suppress tumor growth and enhance cancer therapy. *J Exp Med.* 2018;215(1):115-40.
9. Li Q, et al. Rac1 activates non-oxidative pentose phosphate pathway to induce chemoresistance of breast cancer. *Nat Commun.* 2020;11(1):1456.
10. Wang S, et al. The NET-DNA-CCDC25 inhibitor di-Pal-MTO suppresses tumor

progression and promotes the innate immune response. *Cell Mol Immunol.* 2025;22(6):628-44.
