# Supplementary material for: Tumor-derived neutrophil extracellular trap–associated DNA impairs treatment efficacy in breast cancer via CCDC25-dependent epithelial-mesenchymal transition
Source: J Clin Invest. 2026 Jan 2;136(1):e190557. doi: 10.1172/JCI190557 (PMC12721893; doi:10.1172/JCI190557)

Full unedited blot for Figure 7C

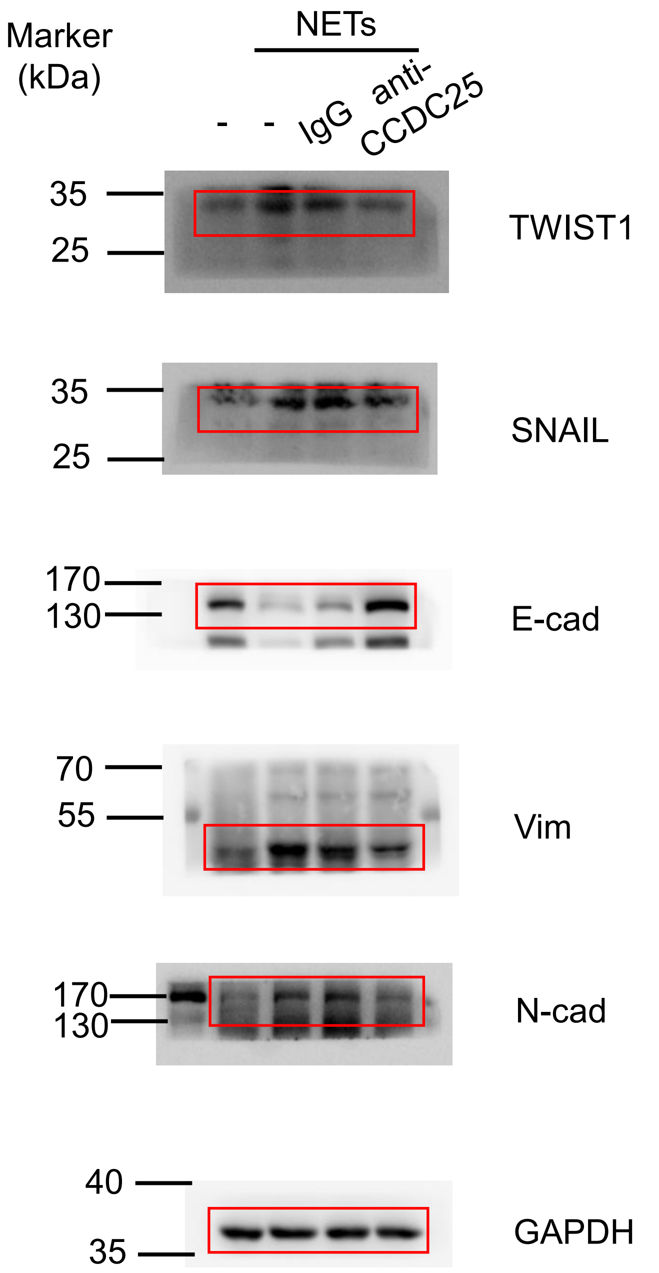

Full unedited blot for Figure 7H

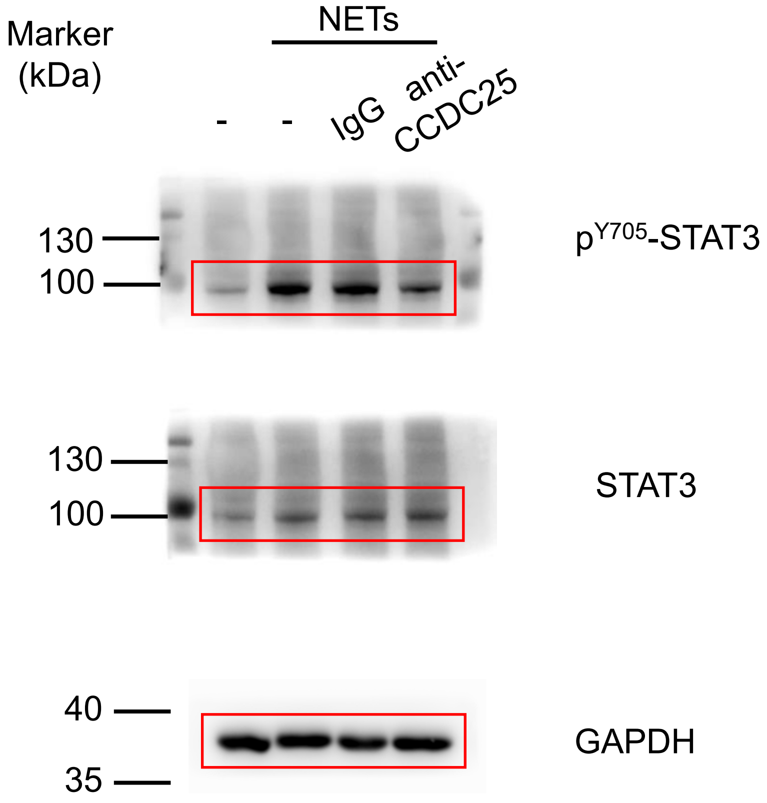

Full unedited blot for Figure 7K

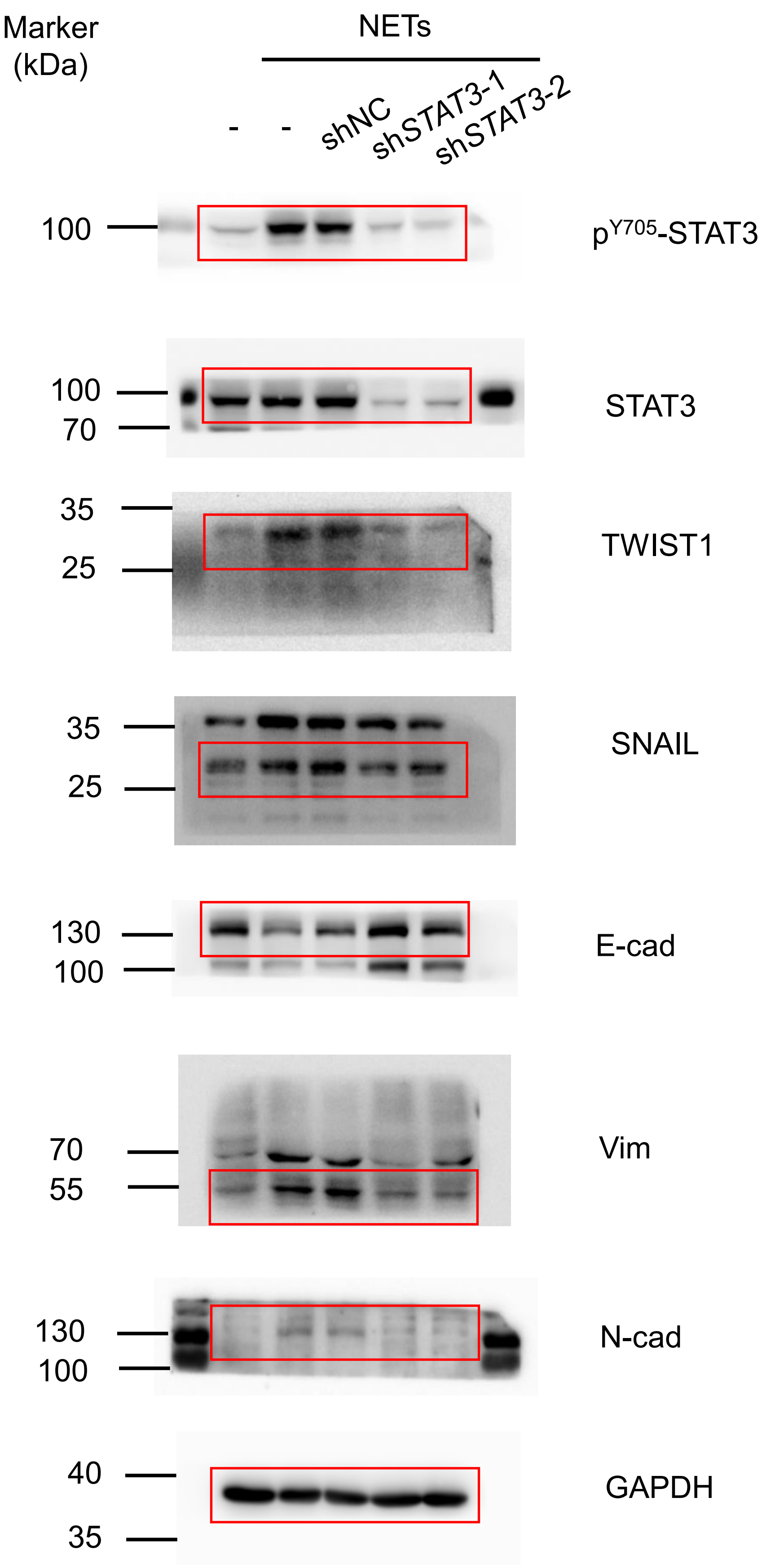

Full unedited blot for Supplemental Figure 5K

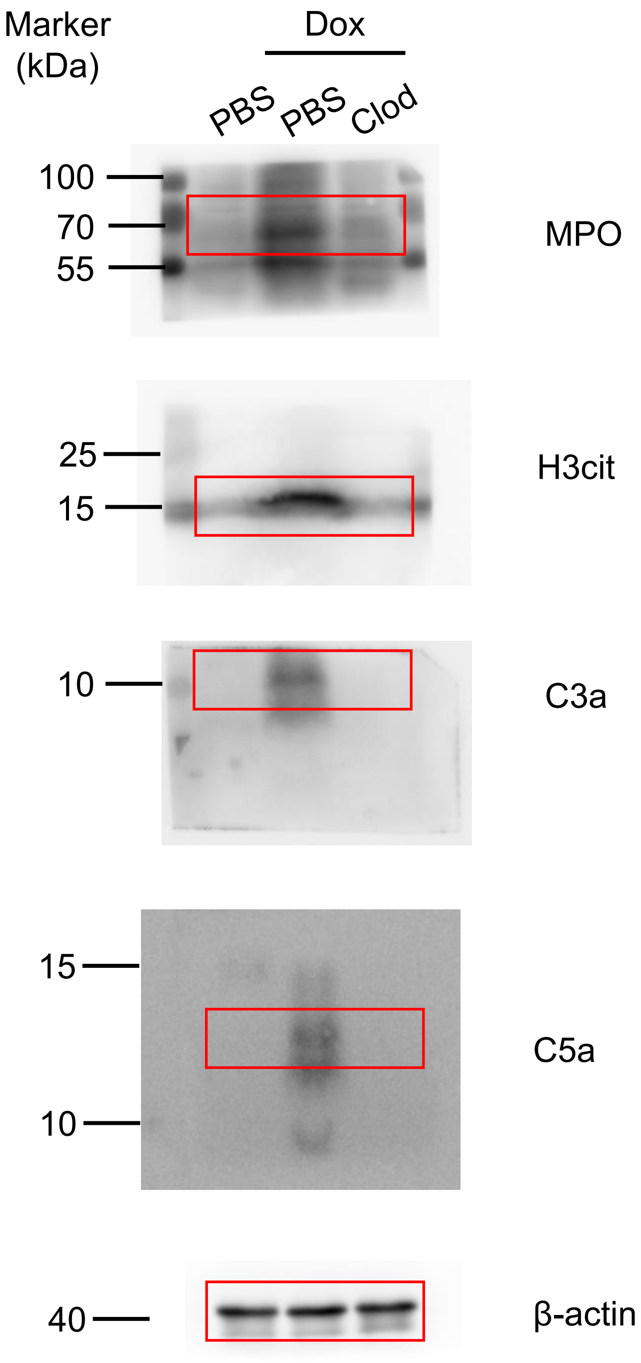

Full unedited blot for Supplemental Figure 6G

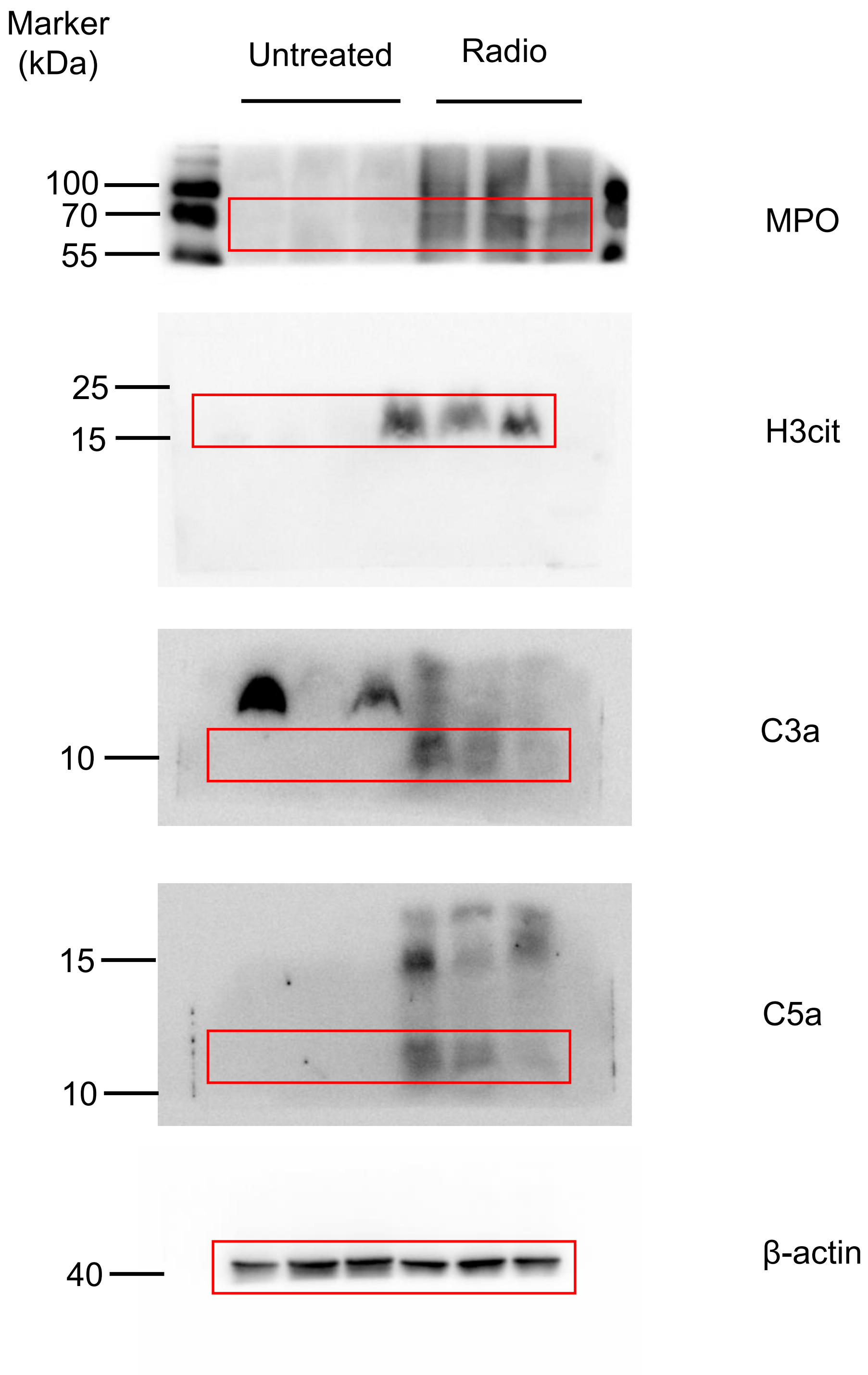

Full unedited blot for Supplemental Figure 7C

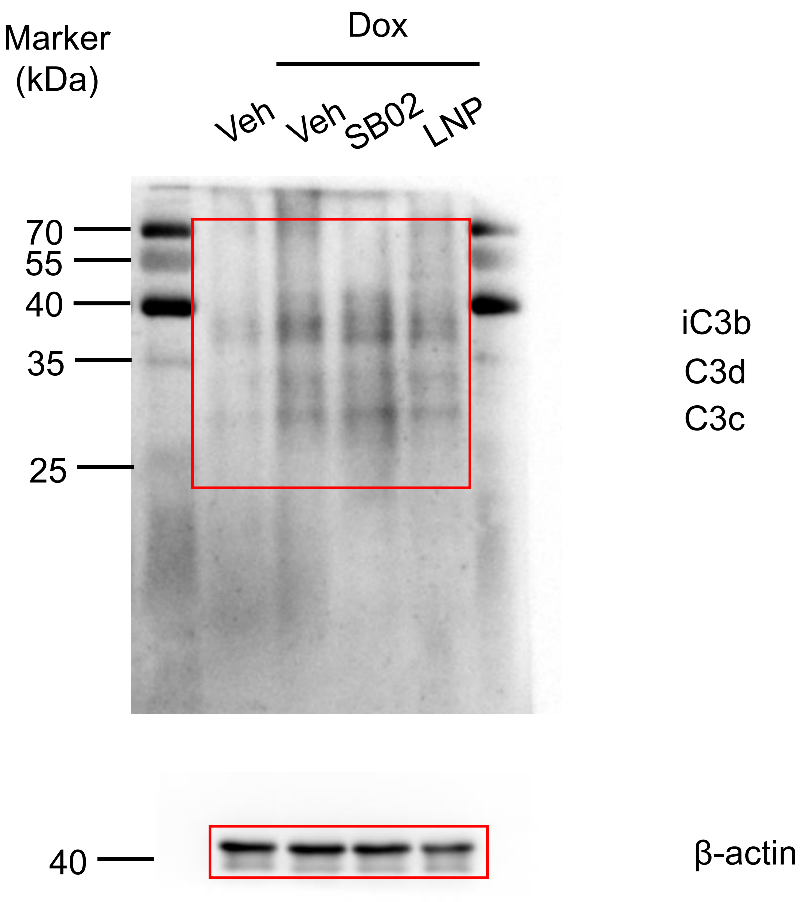

Full unedited blot for Supplemental Figure 7D

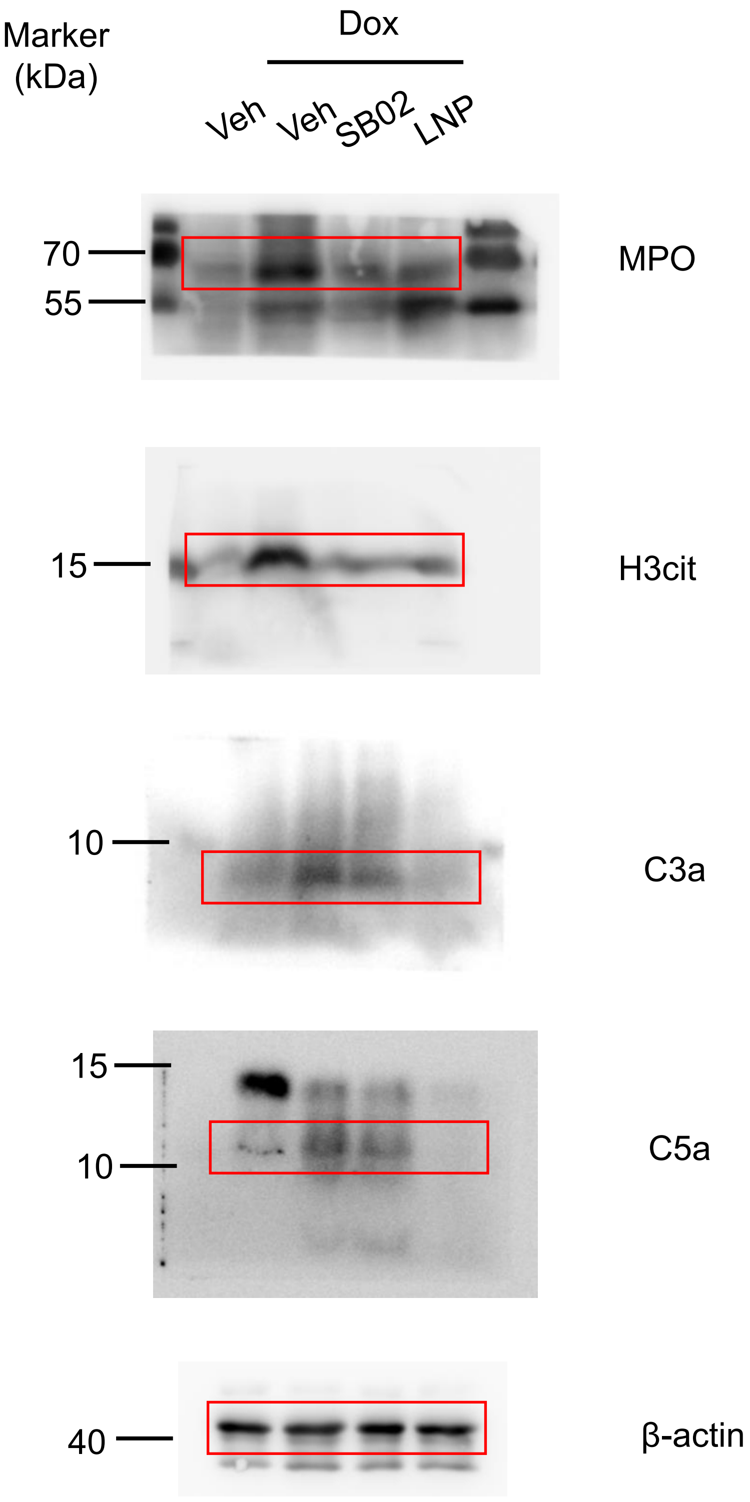

Full unedited blot for Supplemental Figure 9B

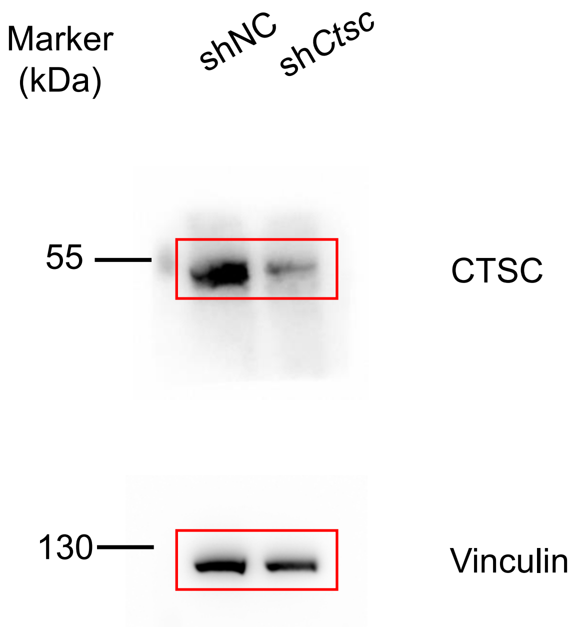

Full unedited blot for Supplemental Figure 10E

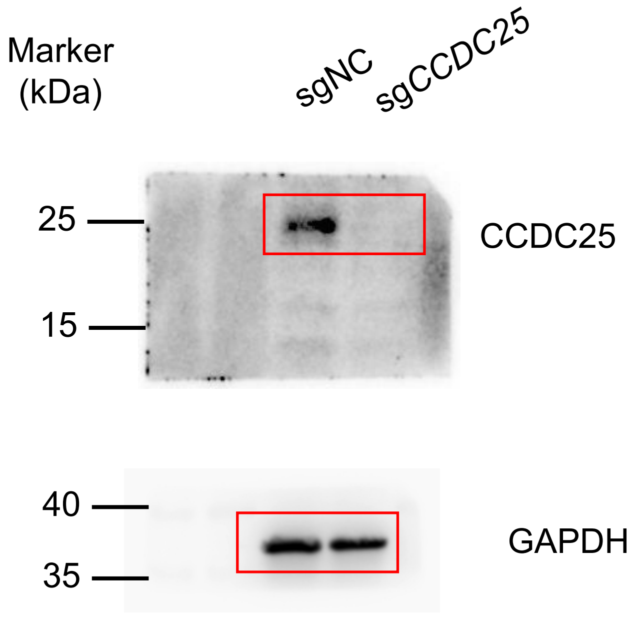

Full unedited blot for Supplemental Figure 10F

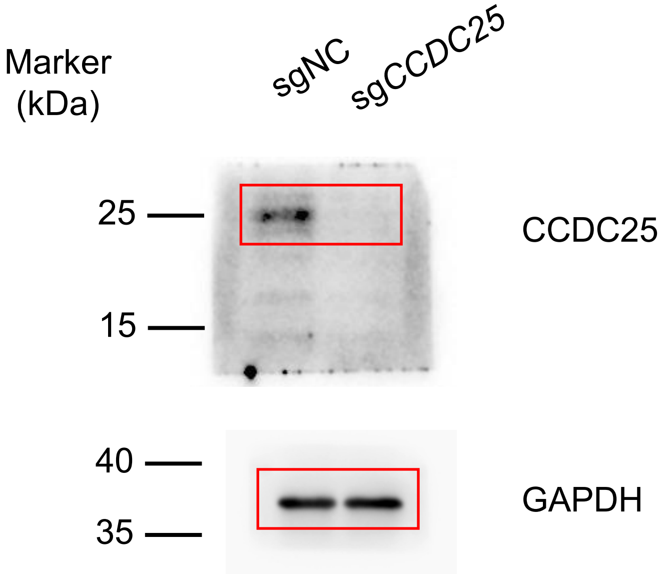

Full unedited blot for Supplemental Figure 10K

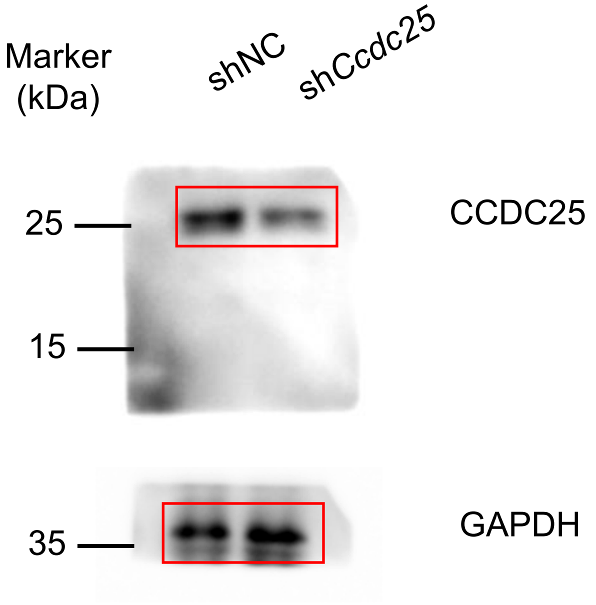

Full unedited blot for Supplemental Figure 11K

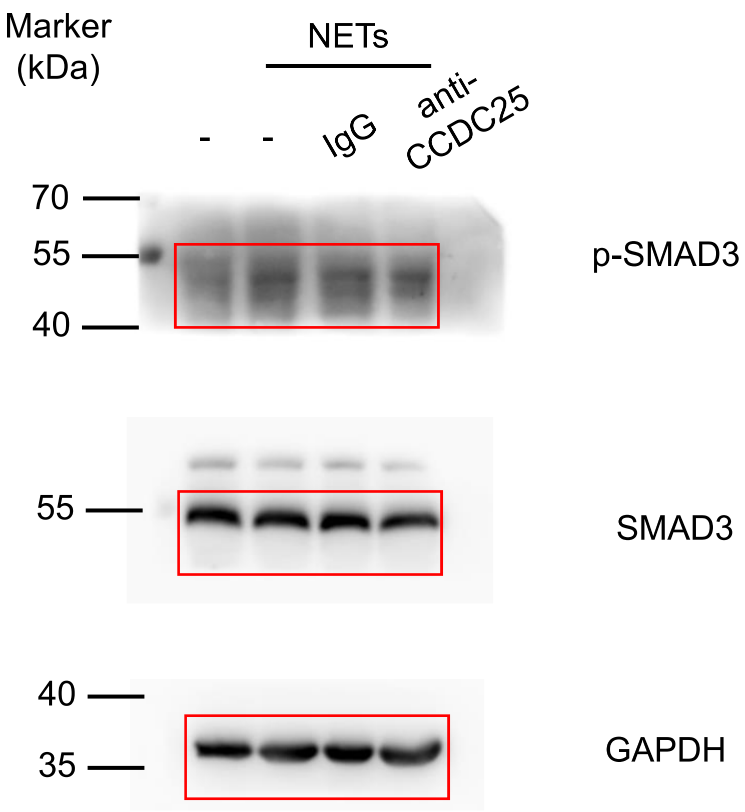

Full unedited blot for Supplemental Figure 11L

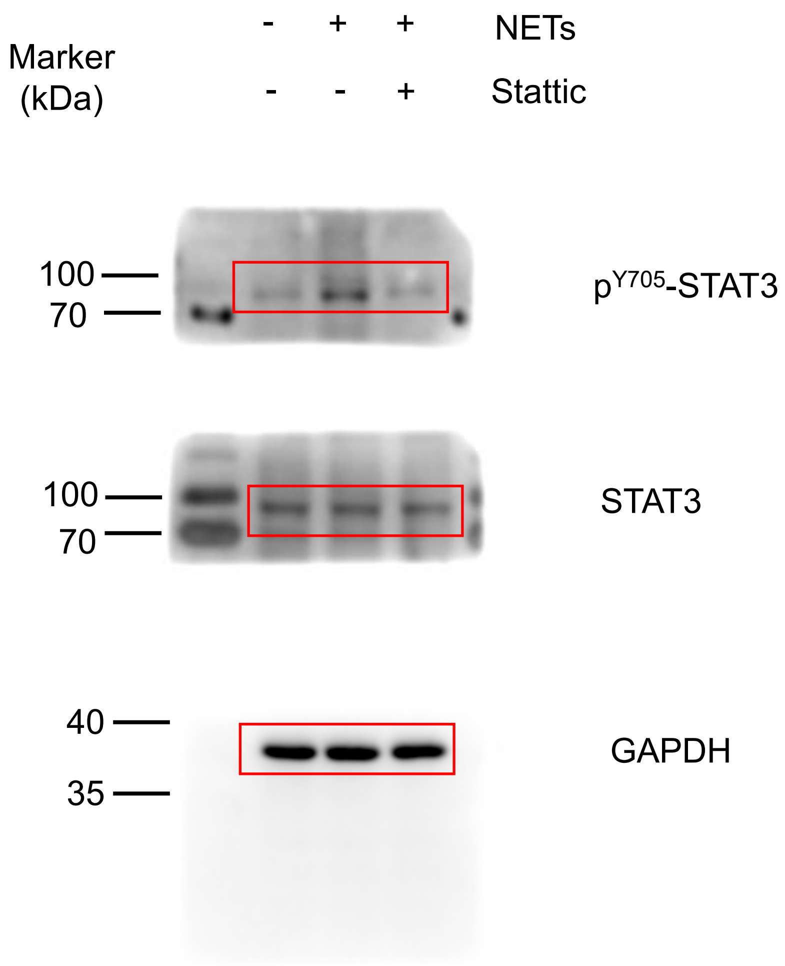

Full unedited blot for Supplemental Figure 12A

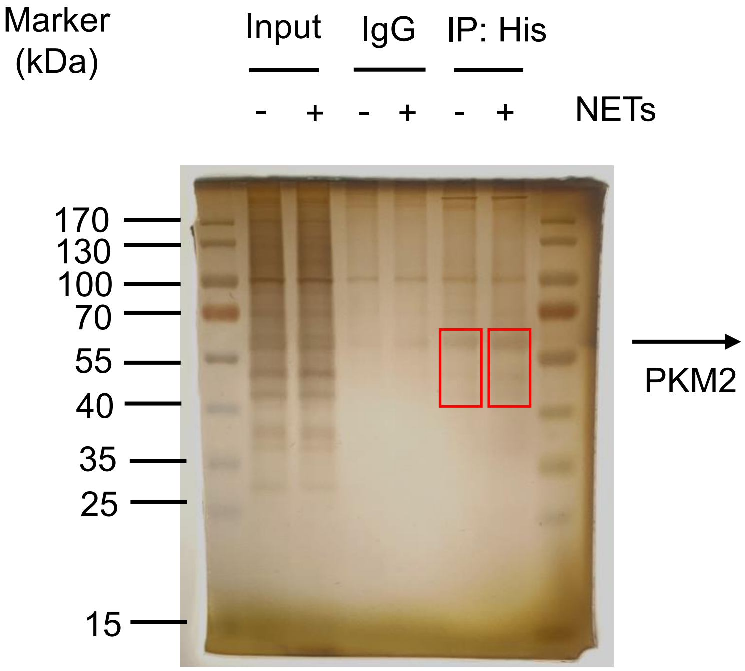

Full unedited blot for Supplemental Figure 12D

IP: His

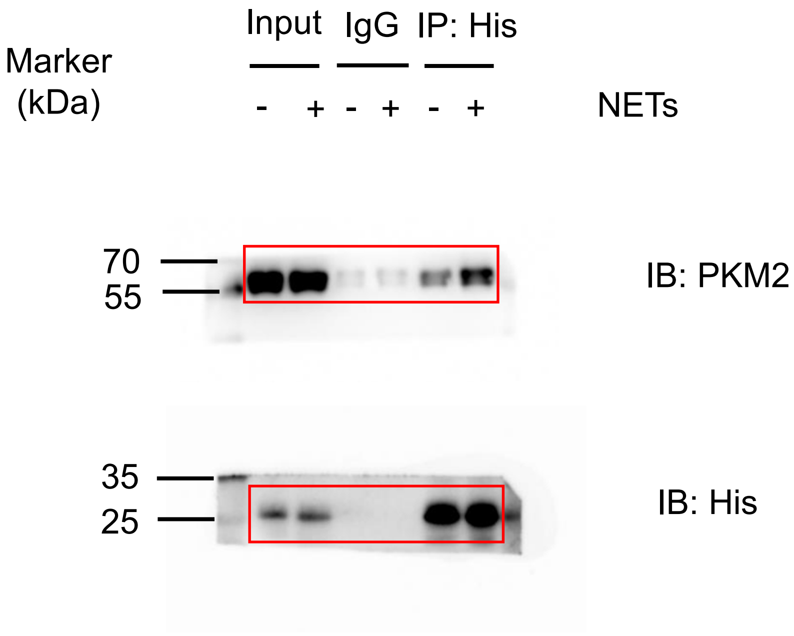

Full unedited blot for Supplemental Figure 12E

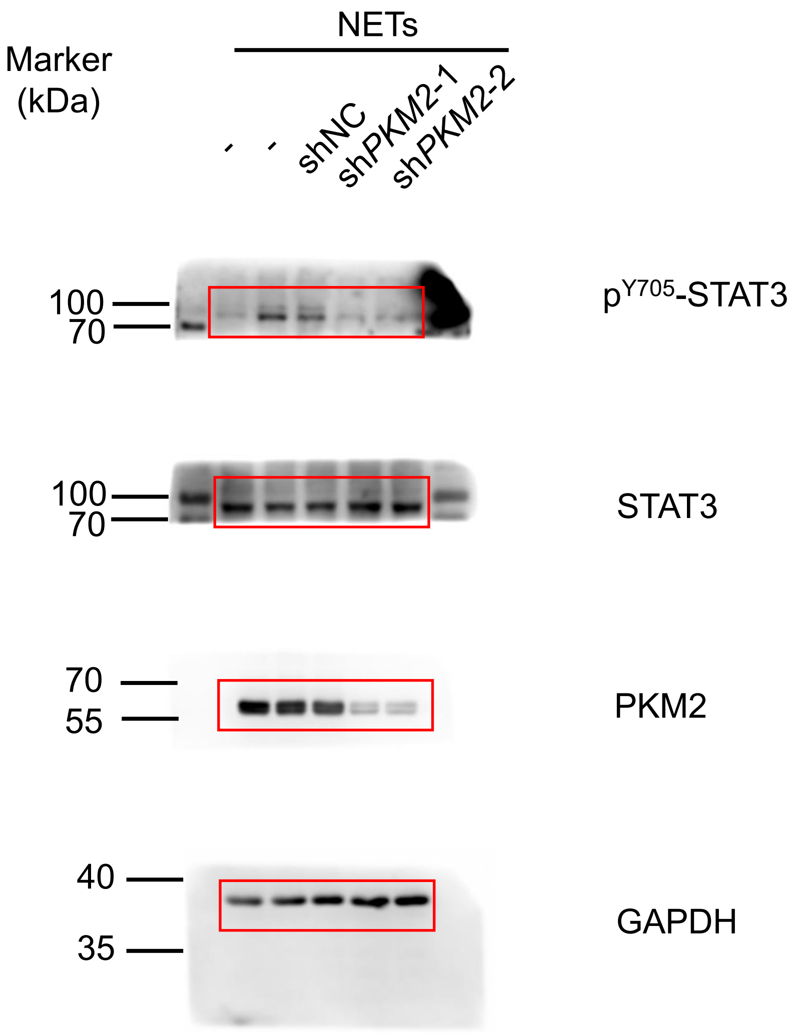

Full unedited blot for Supplemental Figure 12F

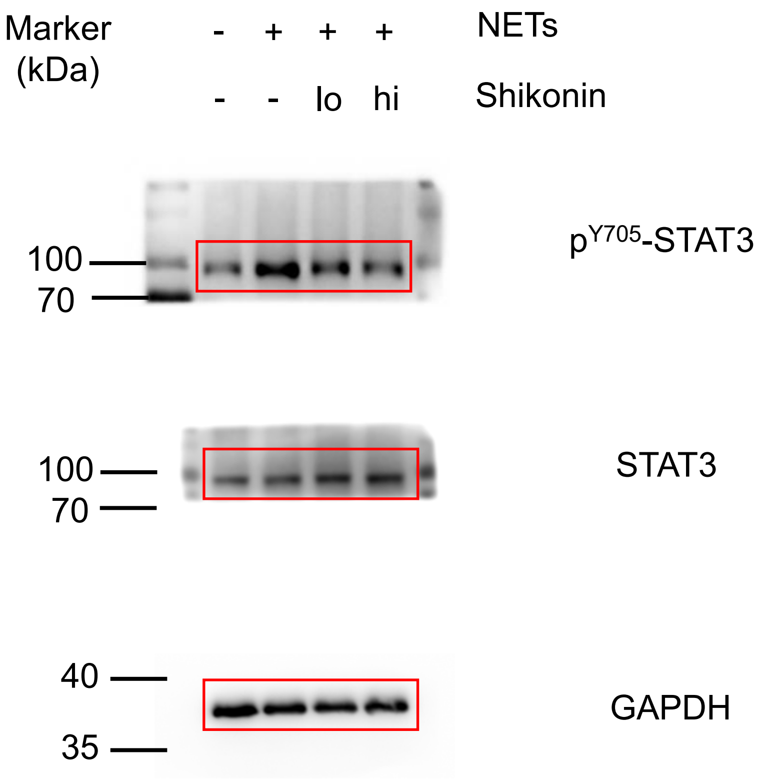

Supplement: Unedited blot and gel images [file jci-136-190557-s039.pdf]
